# Taxonomy‐based hierarchical analysis of natural mortality: polar and subpolar phocid seals

**DOI:** 10.1002/ece3.4522

**Published:** 2018-10-16

**Authors:** Irina S. Trukhanova, Paul B. Conn, Peter L. Boveng

**Affiliations:** ^1^ Polar Science Center Applied Physics Laboratory University of Washington Seattle Washington; ^2^ Marine Mammal Laboratory Alaska Fisheries Science Center NOAA National Marine Fisheries Service Seattle Washington

**Keywords:** Bayesian hierarchical models, natural mortality, phocid seals

## Abstract

Knowledge of life‐history parameters is frequently lacking in many species and populations, often because they are cryptic or logistically challenging to study, but also because life‐history parameters can be difficult to estimate with adequate precision. We suggest using hierarchical Bayesian analysis (HBA) to analyze variation in life‐history parameters among related species, with prior variance components representing shared taxonomy, phenotypic plasticity, and observation error. We develop such a framework to analyze U‐shaped natural mortality patterns typical of mammalian life history from a variety of sparse datasets. Using 39 datasets from seals in the family Phocidae, we analyzed 16 models with different formulations for natural morality, specifically the amount of taxonomic and data‐level variance components (subfamily, species, study, and dataset levels) included in mortality hazard parameters. The highest‐ranked model according to DIC included subfamily‐, species‐, and dataset‐level parameter variance components and resulted in typical U‐shaped hazard functions for the 11 seal species in the study. Species with little data had survival schedules shrunken to the mean. We suggest that evolutionary and population ecologists consider employing HBA to quantify variation in life‐history parameters. This approach can be useful for increasing the precision of estimates resulting from a collection of (often sparse) datasets, and for producing prior distributions for populations missing life‐history data.

## 1 INTRODUCTION

Knowledge of life‐history traits such as age of sexual maturity, fecundity, and survival are fundamental to the study of evolutionary ecology and to applied conservation. Evolutionary ecologists study how life‐history traits differ among species and higher taxonomic groups, and how constraints and trade‐offs among life‐history parameters shape evolution (Roff, 1992; Stearns, 1992). According to this theory, closely related species will often have similar life histories owing to their shared evolutionary history (Kindsvater, Mangel, Reynolds, & Dulvy, 2016). At the same time, life‐history parameters can vary among and between populations of the same species in response to differences in environmental capacity, resource availability, and intraspecific and conspecific interactions (e.g., Johnson & Zuniga‐Vega, 2009; Kindsvater et al., 2016), a phenomenon known as phenotypic plasticity (Stearns, 1992).

In conservation biology, life‐history parameters are crucial pieces of information when developing models used to inform management and conservation planning because they determine the rate at which a population can grow or decline and explain the nature of population persistence through time (Williams, Nichols, & Conroy, 2002). Life‐history parameters are also fundamental inputs to population models. Such models can be “retrospective”—for instance, those fitted to historical data to explain past population changes (as with integrated population models and fisheries models; for example, Besbeas, Freeman, Morgan, & Catchpole, 2002; Newman, Buckland, Lindley, Thomas, & Fernandez, 2006; Quinn & Deriso, 1999)—or “prospective,” as with models used to forecast population trend or viability (e.g., Beissinger & McCullough, 2002; Caswell, 2014).

Unfortunately, knowledge of life‐history parameters is frequently lacking in many species and populations, often because they are cryptic, elusive, or logistically challenging to study, but also because life‐history parameters can be difficult to estimate with adequate precision. For instance, the productivity of fish stocks (a product of fecundity and early stage survival) is a notoriously difficult parameter to estimate using data from a single population (Conn, Williams, & Shertzer, 2010). In such situations, it is sensible to leverage the strength of multiple datasets within a joint analysis to specify reasonable means and variances for imprecise or missing life‐history parameters. Joint analyses allow researchers to combine findings of related, but independent, studies and aggregate information in order to achieve higher precision than would be possible using data from a single dataset (Greenland & O'Rourke, 2008). However, considerable variation in estimates among studies can arise through differences in study design, biases due to methodological flaws and through random noise (Higgins, Thompson, & Spiegelhalter, 2009). Such differences are readily accommodated using Bayesian hierarchical models. The advantages of Bayesian analysis include full allowance for all sources of parameter uncertainty, the opportunity to “borrow strength” from multiple studies when estimating individual effects, and the ability to make predictions for future studies (Higgins et al., 2009). Bayesian methods also offer increased flexibility for performing more complex analyses (Rhodes et al., 2016), can incorporate missing or unbalanced data, and can be used to estimate prior probability distributions in cases where initial information is scarce or does not exist (Ogle, Barber, & Sartor, 2013; Ogle et al., 2014).

Hierarchical analysis has been used in fisheries, where critical life‐history information on individual species is often lacking or is poorly estimated (Myers & Mertz, 1998). It has been successfully employed to estimate a number of life‐history parameters or functions thereof, including fish stock productivity (steepness; Dorn, 2002; Forrest, McAllister, Dorn, Martell, & Stanley, 2010; Michielsens & McAllister, 2004; Shertzer & Conn, 2012), and natural mortality rates (Hewitt et al., 2007; Jensen, 1997; Pauly, 1980). In such analyses, investigators often relate life‐history parameters to more easily measured quantities such as size, age at maturity, or maximum age (Gislason, Daan, Rice, & Pope, 2010). In this study, we suggest using hierarchical Bayesian analysis (hereafter, HBA) to analyze variation in life‐history parameters. However, instead of morphological metrics, we propose using taxonomic relations among species to guide construction of alternative models and using model selection to identity an appropriate level of taxonomic resolution.

Although our basic modeling framework could be applied to multiple life‐history parameters, we concentrate here on quantifying natural morality. Natural mortality is crucial for population management, but is also difficult to estimate, particularly for exploited populations subject to both natural and harvest mortality (Pauly, 1980). It is also one of the most critical and important parameters that both shapes life histories through natural selection (Jørgensen & Holt, 2013). Being subject to considerable evolutionary constraint, we might expect it to be amenable to an analysis where variation in natural mortality curves is structured taxonomically.

Conducting a hierarchical analysis of natural mortality from different data types requires conducting inference on a common scale. We propose using the survival and hazard functions (Cox & Oakes, 1984) as a common currency for analyzing mortality data reported for different time periods or age ranges. In mammals, Caughley (1966) recognized three major life stages including a juvenile stage characterized by a relatively high mortality rate, an adult stage characterized by a relatively low mortality, and a senescent stage where mortality increases again. These three stages can be represented with a U‐shaped hazard rate curve.

This study is structured as follows. First, we introduce the survival and hazard functions mathematically, showing how these functions are related to age‐specific survival probabilities, and introduce our preferred hazard rate formulation based on a reduced additive Weibull (RAW) formulation (Choquet, Viallefont, Rouan, Gaanoun, & Gaillard, 2011; Xie & Lai, 1996). Next, we introduce a generic hierarchical model that can be used to simultaneously analyze data from multiple disparate sources, including (a) the age structure of harvests, (b) survival‐at‐age data (i.e., survival associated with a single age), and (c) survival estimates for age‐ranges (e.g., “adult” survival; ages 1–5 survival). We also address strategies for coping with estimates that do not have accompanying estimates of precision. Next, we describe how to incorporate phylogenetic structure into natural mortality parameters. We then use our approach to conduct an analysis of 39 datasets from 11 phocid seal species that inhabit polar or subpolar seas, areas of relevance to our research programs. Our analysis includes models structured hierarchically by subfamily, species, study, and dataset. High‐latitude phocid seals are often difficult to study given their wide range and natural history. In addition to providing insights into patterns of taxonomy‐based variation, HBA thus has potential to be extremely useful for providing prior distributions for population modeling and analysis of data‐poor populations. We provide an example of estimating such a prior for a population of ribbon seals *Histriophoca fasciata*.

## 2 MATERIALS AND METHODS

### 2.1 Survival models

Before describing a HBA model for natural mortality, it is useful first to describe a common currency for modeling. For instance, one might have access to two point estimates from the literature, one of which gives survival probability for a specific age, and another which gives cumulative survival over the first several years of life. How can both estimates be used as data points within the same analysis given the difference in scale?

To get around this dilemma, researchers in medical fields have long appealed to survival analysis using failure time distributions (e.g., Cox & Oakes, 1984). Specifically, let *T* be a non‐negative continuous random variable representing the age of an animal when it dies. This “waiting time distribution” can be characterized with probability density function *f*_*t*_ and cumulative distribution function (CDF)

$$F_{t}=\text{Pr}\left(T<t\right)$$

Then the complement of the CDF, the survival function

$$S_{t}=\text{Pr}\left(T\geq t\right)=1-F_{t}$$

is the probability of surviving until *t*. Note that *S*_*t*_ is the “survivorship schedule” familiar to ecologists as the life table quantity l(x) (see Gotelli, 2001, p. 53).

In many instances, it is more straightforward to work with the hazard function than the survival function or related probability distributions. In the case of mortality, the hazard function is the instantaneous death rate and can be defined as follows:

$$\lambda_{t}=f_{t}/S_{t}$$

The relationship between the hazard and survival functions can also be specified as follows:

$$S_{t}=\exp\left(-\int_{t}^{\infty }\lambda_{x}dx\right),$$

so that, for instance, a constant hazard *c* leads to a survivor function (survivorship schedule) of

$$S_{t}=\exp\left(-\text{ct}\right)$$

Under this framework, the conditional probability of surviving to age *t *+* *1 given that an animal has already survived to age *t* is simply

(1) $$\phi_{t}=\frac{S_{t+1}}{S_{t}}$$

Similar calculations can be made to model natural mortality over different age ranges; hazard and survival functions thus provide a unified basis for modeling survival data on different scales (Ergon, Borgan, Nater, & Vindenes, 2018).

There are a variety of hazard functions that have a U shape appropriate to mammalian (and other species) life histories with prior use in ecology, including Siler (1979) and various Weibull (Bebbington, Lai, & Zitikis, 2007a,b) functions. Choquet et al. (2011) studied the performance of different survival curve formulations, preferring a reduced additive Weibull (RAW) survival function (Xie & Lai, 1996) which has the added advantage of only requiring three parameters. This formulation is specified using survival and hazard functions given as follows:

(2) $$S_{t}=\exp\left(-{\left(\mathrm{at}\right)}^{b}-{\left(\mathrm{at}\right)}^{\frac{1}{b}}-\text{ct}\right)$$

$$\lambda_{t}=\text{ab}{\left(\text{at}\right)}^{b-1}+\frac{a}{b}{\left(\text{at}\right)}^{\frac{1}{b}-1}+c,$$

where *a *>* *0, *b *>* *1, *c *≥* *0 are parameters to be estimated. We use this formulation in all subsequent modelling efforts.

### 2.2 Hierarchical modelling framework

We describe a HBA modelling framework specific to analysis of natural mortality. As with all Bayesian analyses, this involves combining prior information with a likelihood (or product likelihood in our case) to generate a posterior distribution for the parameters of interest. Symbolically, we can write the posterior as

$$\text{Pr}\left(\theta |Y\right)\propto \text{Pr}\left(\theta \right)\prod_{d}\text{Pr}\left(Y_{d}|\theta \right),$$

where **θ** denotes the set of all parameters in the model, **Y** denotes all data, and the subscript *d* indexes these quantities by dataset. The form of the likelihoods Pr (Y_*d*_ | **θ**) depends on the type of data being analyzed; ultimately the goal is to probabilistically relate data or reported estimates to RAW survival functions employing parameters **θ**. We propose to include taxonomy‐based structure within the joint prior distribution, Pr(**θ**). We now describe these two modelling components in turn.

#### 2.2.1 Likelihoods of common data types

We describe likelihood functions for three major types of mortality‐related data encountered in the literature, namely: survival probability by age (often annual), survival probability by age range, and age structure of harvests (i.e., cohort or life table data). Since survival probabilities are naturally bounded by the interval (0, 1), we propose to model survival on the logit scale. Probit scale could be another possible choice in situations when convergence improvement is needed. We also make allowances for harvested populations by incorporating study‐specific prior distributions for harvest rates.

In harvested populations, a major challenge is that survival estimates reported in the literature often reflect total mortality and, as a result, natural mortality must be separated from human‐caused mortality to quantify the relative effects of harvest versus natural causes of animal death. To separate the two processes, it will often be necessary to have a point estimate or prior distribution for harvest rate. In analyses that include both harvested and non‐harvested populations, one can presumably get away with an imprecise prior, although this means that non‐harvested populations will contribute more to inferences about natural mortality.

Any approach for simultaneously modelling natural and harvest mortality must make assumptions about the temporal distribution of mortality. For instance, if natural and harvest mortality operate at constant rates throughout a year, one might use a formulation for total survival based on the Baranov catch equation, which assumes constant, overlapping hazards for both processes (Miller & Andersen, 2008). In contrast, if harvest operates as a “pulse,” one might use a model where harvest mortality operates before, in between, or after natural mortality. In likelihoods described below, we use the latter specification, as it is most relevant to our phocid seal example. We also make harvest rate age and time invariant, although this constraint can certainly be revisited in specific applications.

##### Age‐specific survival

Let

${\tilde{\phi }}_{t,d}$ denote the annual survival probability of age class *t* from dataset *d* as reported in the literature. We model these data using a logit‐normal distribution, such that

$$\text{logit}\left({\tilde{\phi }}_{t,d}\right)∼\text{Normal}\left(\text{logit}\left(\phi_{t,d}\right),\tau_{t,d}^{-1}\right),$$

where **τ**_t,d_ is a precision value representing observation error and ϕ_*t,d*_ is a function of natural and harvest mortality. Specifically, we set

(3) $$\phi_{t,d}=\left(1-h_{d}\right)/S_{t+1,d},$$

where *h*_*d*_ is the harvest rate associated with dataset *d* and *S*_*t,d*_ is the survival function value for age *t* and dataset *d* (see the next section for more information on how these are formulated for specific datasets). In all applications in this study, we use a diffuse prior distribution on harvest, where

$$h_{d}∼\text{Uniform}\left(0,2{\tilde{h}}_{d}\right),$$

and ${\tilde{h}}_{d}$ is a rough estimate based on information from the literature (see Supporting information Appendix S1 for a description of how such rough estimates can be calculated). Note that ${\tilde{h}}_{d}$ can be set to an arbitrarily small value for unharvested populations.

We have encountered several ways in which authors report precision in peer‐reviewed literature and scientific reports. In a best case scenario, standard errors are reported along with estimates, and these can be used directly to set precision as

(4) $$\tau_{t,d}={\tilde{\phi }}_{t,d}^{2}{\left(1-{\tilde{\phi }}_{t,d}\right)}^{2}{\left[\hat{\mathrm{SE}}\left({\tilde{\phi }}_{t,d}\right)\right]}^{-2}.$$

This formulation uses the delta method to approximate the precision of estimates on the logit scale. In other cases, one may not be so fortunate; for instance, sometimes researchers report sample size (but no standard error), a confidence interval instead of a standard error, or simply just an estimate with no indication of sample size or precision. We have developed procedures for producing τ_*t,d*_ values for each of these data types. In particular, we use properties of the binomial distribution to calculate precision where possible. In the absence of any information about likely standard error, we used a conservative procedure that assigned relatively imprecise values; see Supporting information Appendix S2 for more information on these procedures.

##### Survival for age‐ranges

Another way survival data are reported in the literature is over age ranges. For instance, an author might report a “subadult” survival rate that pertains to ages 1–4, or a single adult survival probability that applies to ages 5+. We still desire to use such data in analysis, even though our survival and hazard functions are age specific.

To incorporate these data, we suggest matching the reported estimate to a weighted average of survival probabilities, where the weight is proportional to the expected number of individuals alive in each age class. For instance, let *t*_1_ be the beginning of the age range and *t*_2_ be the last age represented. Note that if the final age is not specified (e.g., ages 5+), a “large” age can be used for *t*_2_, such that the probability of living past *t*_2_ is negligible. In this case, we suggest modeling reported survival as

$\text{logit}\left({\tilde{\phi }}_{[t_{1},t_{2}],d}\right)∼\text{Normal}\left(\text{logit}\left(\phi_{[t_{1},t_{2}],d}\right),\tau_{[t_{1},t_{2}],d}^{-1}\right),$ where

$\phi_{[t_{1},t_{2}],d}=\sum_{t=t_{1}}^{t_{2}}\phi_{t,d}\pi_{t,d},$ and

(5) $$\pi_{t,d}=\frac{\prod_{i=t_{1}}^{t}\left(\phi_{i-1,d}\right)}{\sum_{j=t_{1}}^{t_{2}}\prod_{i=t_{1}}^{j}\left(\phi_{i-1,d}\right)}.$$

Note that we use the same definition of survival probability as the previous section (i.e., Equation (3)). If a standard error for the age range estimate is provided, this can be applied as in Equation (4) to produce a value for τ[t_1_,t_2_],d. Otherwise, alternative procedures will be needed (see Supporting information Appendix S2).

##### Age structure of harvests

For harvested fish and wildlife populations, there are often data on the age structure of harvests. Such data represent a number of processes that are difficult to fully disentangle, including recruitment, natural mortality, harvest mortality, and reporting rates. However, when used in conjunction with other data sources, catch‐age data provide the backbone of most modern fisheries stock assessment models (Quinn & Deriso, 1999) and many wildlife monitoring programs (Skalski, Ryding, & Millspaugh, 2010).

We propose to use age‐structure data in a slightly less nuanced manner, as historical records often do not include the auxiliary information that would be necessary to estimate time‐ or age‐specific sampling probabilities (although incorporating these into our modeling framework would be highly desirable if extra data were available). Let *C*_*t,c,d*_ represent the number of animals from dataset *d* and cohort *c* that are aged *a* when harvested (a cohort denotes a group of animals all born in the same year). Then, assuming harvest rates are approximately equal for all ages and time periods, we model

$$\left[C_{1,c,d},C_{2,c,d},\cdots ,C_{T,c,d}\right]∼\text{multinomial}\left(\sum_{t}C_{t,c,d};\pi_{1,c,d},\pi_{2,c,d},\cdots ,\pi_{T,c,d}\right),$$

where the multinomial cell probabilities, π_*t,c,d*_, can be calculated as in Equation [Link]. See the Discussion for possible extensions allowing age‐ or time‐specific harvest rates.

#### 2.2.2 Taxonomy‐based structure of natural mortality

So far we have described likelihoods for common data types that are written in terms of dataset‐specific survival functions. We will now describe how to impart greater structure on these functions, thereby allowing information to be shared across studies. Initially, we thought to impart variation in survival by imposing hierarchical structure on the *a*,* b*, and *c* parameters of Equation (2). However, such models were often unstable (I. Trukhanova, *unpublished data*), resulting in multimodal solutions and MCMC convergence problems. Instead, we report on application of a proportional hazards modeling framework (Cox & Oakes, 1984, section 5.3), whereby hazard rates are multiplied by dataset‐specific adjustment terms. Specifically, the proportional hazards model can be written as follows:

$$\lambda_{t,d}=\psi_{d}\lambda_{t}.$$

**Figure 1:**
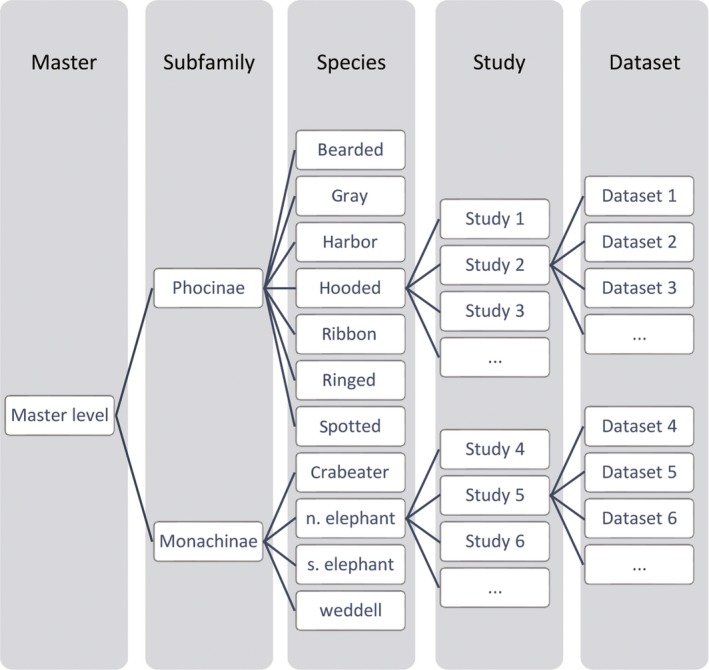
Five‐level hierarchical structure of the models used to estimate survival and hazard rate for phocid seals

In our applications, λ_*t*_ is as in Equation (2), and the adjustment term ψ_*d*_ is a function of covariates. To impart taxonomic structure, we specify a log‐Gaussian hierarchical model for ψ_*d*_, writing it as

(6) $$\log\left(\psi_{d}\right)=\sum_{k\in K_{d}}\in_{k},$$

where $K_{d}\equiv \left\{\text{sub}-\text{family, species, study, dataset}\right\}.$ For a visual depiction of a possible model structure, see Figure 1. Importantly, we can include variance components for all desired taxonomic levels, as well as levels particular to a given population, study, or dataset. This ability can help discriminate between taxonomic effects, phenotypic plasticity (e.g., effects associated with different populations), and sampling artifacts (e.g., effects associated with different datasets).

We specify a Gaussian structure for each of the components in Equation [Link], and conjugate gamma priors for the precision of Gaussian errors to complete our hierarchical specification. Specifically,

$$\in_{k}∼\text{Normal}\left(0,\tau_{k}^{-1}\right),and$$

$$\tau_{k}∼\text{Gamma}\left(\alpha_{k},\beta_{k}\right).$$

Note that values for τ_*k*_, α_*k*_, and β_*k*_ need to be specified by the analyst. In the example below, we set τ_*k*_ = 0.1, α_*k*_ = 1.0, and β_*k*_ = 0.1, which are weakly informative so as to promote shrinkage towards the mean, but flexible enough to allow priors to be overwhelmed when there are sufficient data.

One consequence of using a nonlinear transformation is that survival at higher levels of the model hierarchy (e.g., survival at the species level when a model also includes dataset‐level effects) will be biased if transformed directly to the real scale. In such situations it is common to employ a lognormal bias correction (e.g., Dorn, 2002). For instance, if our model includes species and dataset effects, and we are interested in predicting survival at the species level on the real scale, we need to calculate it using

$$\psi_{\mathrm{species}}=\exp\left(\in_{\mathrm{species}}+0.5\tau_{\mathrm{dataset}}^{-1}\right),$$

as the additional variation associated the dataset level effect will increase the mean response.

### 2.3 Example: phocid seals

To demonstrate our approach, we conducted a HBA of phocid (family Phocidae) seal natural mortality. Phocid seals include two subfamilies, Monachinae and Phocinae, and are physiologically and ecologically adapted to aquatic environments. Many phocids use sea ice for at least some parts of their life cycle (e.g., for resting, giving birth, nursing, or molting). These high‐latitude phocid seals are long‐lived cryptic species inhabiting remote regions that are difficult to access, especially for those with ice‐associated breeding strategies. For such species, direct estimates of abundance and population trends are difficult to obtain but are of conservation interest due to vulnerability of these species to external threats such as climate change and overexploitation.

An indirect way to estimate population growth is from life‐history data. For instance, estimates of age of sexual maturity, fecundity, survival, and maximum life length can be compiled in a Leslie matrix (Leslie, 1945) or similar model, which can then be used to estimate the rate of population increase or decrease. Researchers can also use sensitivity and elasticity analyses to examine consequences of hypothesized changes in vital rates on population trajectories, for instance as a function of different climate or management scenarios (e.g., Hunter et al., 2010).

One of the main obstacles for conducting such analyses for high‐latitude seals is the lack of direct data on natural mortality rates. So far, only a few species have been amenable to survival estimation, usually from mark–recapture studies of individuals recognizable from tags, unique scars, or other markings. However, these studies are rare because they require a high degree of philopatry in order to have requisite detection probabilities and to ensure estimates of mortality do not also include permanent emigration. There are also numerous historical datasets that documented the age structure of commercial and subsistence harvests that might be used to inform natural mortality rates. However, these historical datasets are difficult to interpret individually, as one cannot readily separate harvest and natural mortality. As such, phocids represent a well‐defined case study for use of HBA, as the data available are quite heterogeneous and borrowing strength from some datasets can help extract information from others (the “Robin Hood effect”; Punt, Smith, & Smith, 2011). Although our analysis focuses on phocid seals, our models should be applicable to survival of other large mammal species.

#### 2.3.1 Data and modelling framework

**Figure 2:**
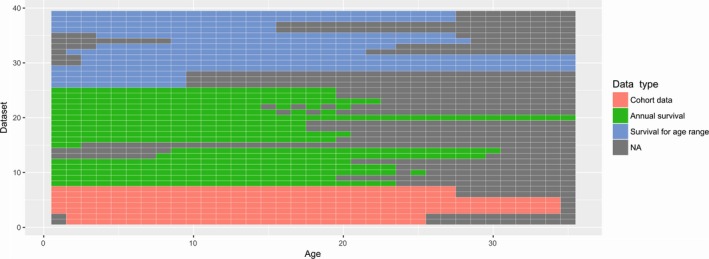
Structure of datasets used in meta‐analysis reflecting age‐ and data type‐specific distribution of missing values

We conducted a HBA of 39 datasets representing 11 high‐latitude seal species from both sub‐families (Weddell *Leptonychotes weddellii*, crabeater *Lobodon carcinophaga*, southern elephant *Mirounga leonina*, northern elephant *Mirounga angustirostris*, ringed *Phoca hispida*, ribbon, bearded *Erignathus barbatus*, spotted *Phoca largha*, hooded *Cystophora cristata*, gray *Halichoerus grypus*, and harbor *Phoca vitulina* seals) for which the data were found in the published sources (Supporting information Appendix S1: Table S2). These data were gathered from 25 different studies; however, when sexes were differentiated or data were collected in different time periods we treated data from each sex and/or time period as a separate dataset. The modeled data included three different types of inputs (age‐specific estimates, estimates of age ranges, and cohort data), and considerable heterogeneity in covered ages (Figure 2). We fitted a total of 16 hierarchical models to these data, using likelihoods and prior distributions as previously described. The models differed based on the specification of natural mortality: specifically the amount of taxonomy‐based and data‐level variance components included in the proportional hazard model. In particular, we included all combinations of the following variance components: subfamily, species, study, and dataset. We then used deviance information criterion (DIC; Spiegelhalter, Best, Carlin, & Van Der Linde, 2002) to compare support for alternative models.

#### 2.3.2 Parameter estimation

We used JAGS ver. 4.2.0 (Plummer, 2003) to simulate posterior samples from our Bayesian hierarchical model via Markov chain Monte Carlo (MCMC). For each model, we simulated three Markov chains; the length of the chain, number of burn‐in iterations, and thinning rate were set so as to achieve reasonable convergence diagnostics while ensuring at least 1,000 draws from the posterior distribution (Table 1). Convergence was assessed visually and by verifying that the Gelman‐Rubin statistics ($\hat{R}$) were less than 1.1, as recommended by Brooks, Gelman, Jones, and Meng (2011). We checked $\hat{R}$ for all other parameters to make sure they were within the 1.1 limit. Given that the DIC has problems counting parameters in missing data models (Celeux, Forbes, Robert, & Titterington, 2006), we also examined parameter counts (pD) to ensure that they were reasonable (Table 1). Data manipulation and model coding was performed in the R programming environment (R Core Team 2017), using the R2jags package (Yu‐Sung & Yajima, 2015) to pass data between R and JAGS.

**Table 1:** Details on model fitting and DIC‐based model selection

Model	Survival parameterization	pD	DIC
3	~master + subfamily + species + dataset	58.6	3767.1
7	~master + subfamily + dataset	60.7	3768.9
13	~master + study + dataset	60.2	3770
9	~master + species + study + dataset	61.6	3770.7
1	~master + subfamily + species + study + dataset	61.9	3770.9
5	~master + subfamily + study + dataset	61.6	3770.9
11	~master + species + dataset	64.5	3774
15	~master + dataset	67.6	3776.6
10	~master + species + study	56	4252.3
2	~master + subfamily + species + study	56.8	4253.9
14	~master + study	58	4254.5
6	~master + subfamily + study	61.7	4258.8
4	~master + subfamily + species	41.7	4802.8
12	~master + species	47.7	4809.8
8	~master + subfamily	40.5	6682.5
16	~master	34.4	11729.9

Models are ordered by decreasing DIC. The models were fit with niter = 15,000 (the total number of iterations of each MCMC chain); burn‐in = 5,000 (the number of iterations discarded before convergence to the stationary distribution); and thin = 10 (the number of MCMC iterations conducted for every such value that was saved, that is, a value of 100 indicates that 1 out of every 100 iterations was saved). pD is the effective number of parameters as output by JAGS and used in DIC computations.

#### 2.3.3 Estimating a prior distribution for a ribbon seal population

One of the advantages of a HBA is the ease to which one can compute a prior distribution of natural mortality. Such a distribution could be used in demographic modeling exercises of a population for which survival data are unavailable, or to help anchor mortality when conducting survival estimation with a sparse dataset. We construct such a prior for a ribbon seal population in the Sea of Okhotsk, Russia, where aerial surveys have recently been conducted (Chernook et al., 2014) but no recent mortality data are available. The population was heavily exploited during the 20th century but currently there is only a small‐scale subsistence harvest (Fedoseev, 2000). Hence, we used a 0.001 harvest rate as an input for our models. Using the highest ranked DIC model, we constructed a prior by simply including an extra ribbon seal dataset in the JAGS analysis, but setting all survival data to “NA.”

## 3 Results

In total, we fit 16 different models to phocid survival data, including all possible combinations of subfamily, species, study, and dataset level variance components (Table 1). All models appeared to converge to their stationary distributions. The highest‐ranked model according to DIC included subfamily, species and dataset parameter variance components (M3; see Table 1). Several other models including dataset, species, and/or study‐level variance components had DIC scores close to that of the top model. However, a dataset effect was present in all eight highest ranked models. Estimates of the number of effective parameters for DIC computations (pD; Table 1) generally increased as the number of variance components in the model increased, suggesting that conducting model selection with DIC scores was likely reasonable in this instance.

**Figure 3:**
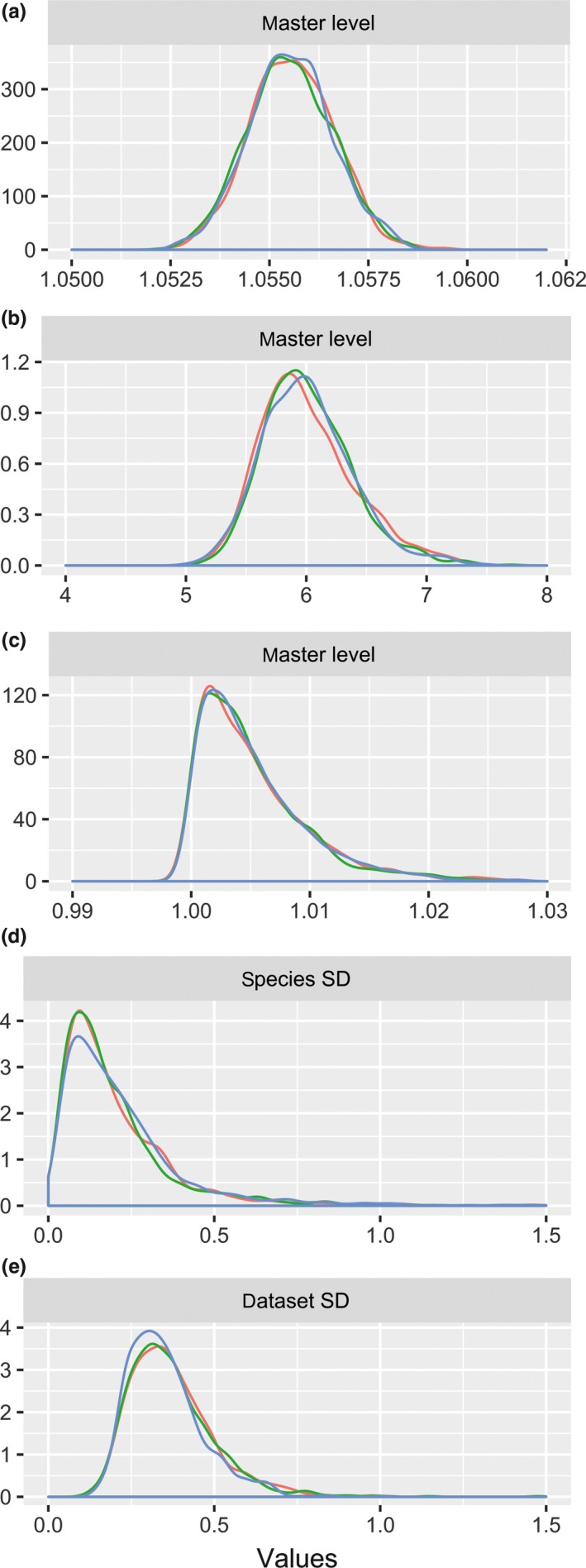
Posterior density distributions (three MCMC chains) for *a, b,* and *c* parameters and their associated variances on dataset and species levels (all back‐transformed to real scale)

**Figure 4:**
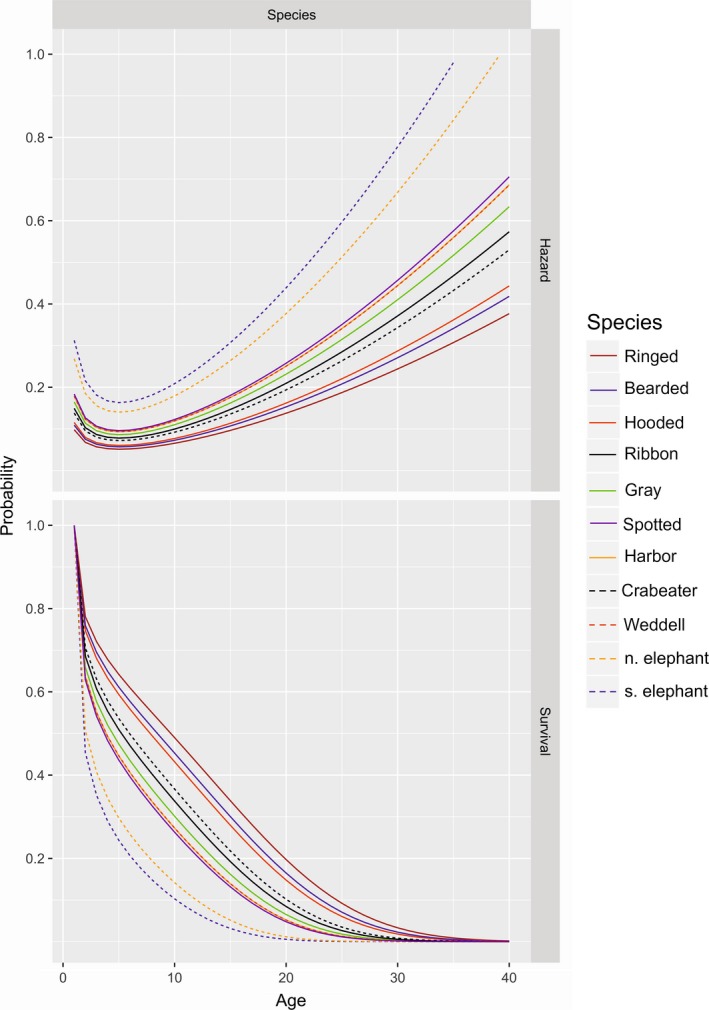
Hazard and survival functions for 11 phocid seal species as estimated from the best‐fitting model, M3

Using the highest ranked DIC model (M3), posterior means and 95% credible intervals for master‐level *a*,* b*, and *c* parameters were as follows: *a *=* *0.05402(0.0539, 0.0541), *b *=* *2.6067(2.601, 2.613), and *c *=* *0.0057(0.0053, 0.0061). Posterior means and standard errors for all parameters from M3 are provided in Supporting information Appendix S3. The expected variation in hazard and survival functions increases as one progresses to the species and dataset levels (Figure 3). Our analysis indicated substantial variation in species‐specific hazard rates and survival functions (Figure 4, Supporting information Appendix S1), with southern elephant seals having the lowest survival rates, while ringed seals had the highest.

Survival functions exhibited a similar pattern for all 11 phocid seal species considered in the analysis, which is to be expected given the proportional hazards specification. Yearling survival among all species was in the range of 45%–78%. Southern elephant and northern elephant seals were characterized by the highest natural mortality levels and the earliest senescence, with the cumulative survival of only 10.3%–14.2% to age 10 and dropping below 1% by the age of 19 and 21, respectively (Figure 4). On the other extreme, ringed seals were characterized by the highest survival, with 49% surviving to age 10 and 1% surviving to age 28. Similarly, the hazard functions suggest that senescence begins at a relatively early age for southern and northern elephant seals, where hazard rates start increasing rapidly from ages 12–15. Bearded and ringed seals’ hazard functions had the lowest rate of increase compared with other species, suggesting that senescence may not be as pronounced in these species. However, the precision of hazard curves is poor at older ages owing to decreased sample size in older ages; thus, any conclusions about the age of senescence should be made with caution.

**Figure 5:**
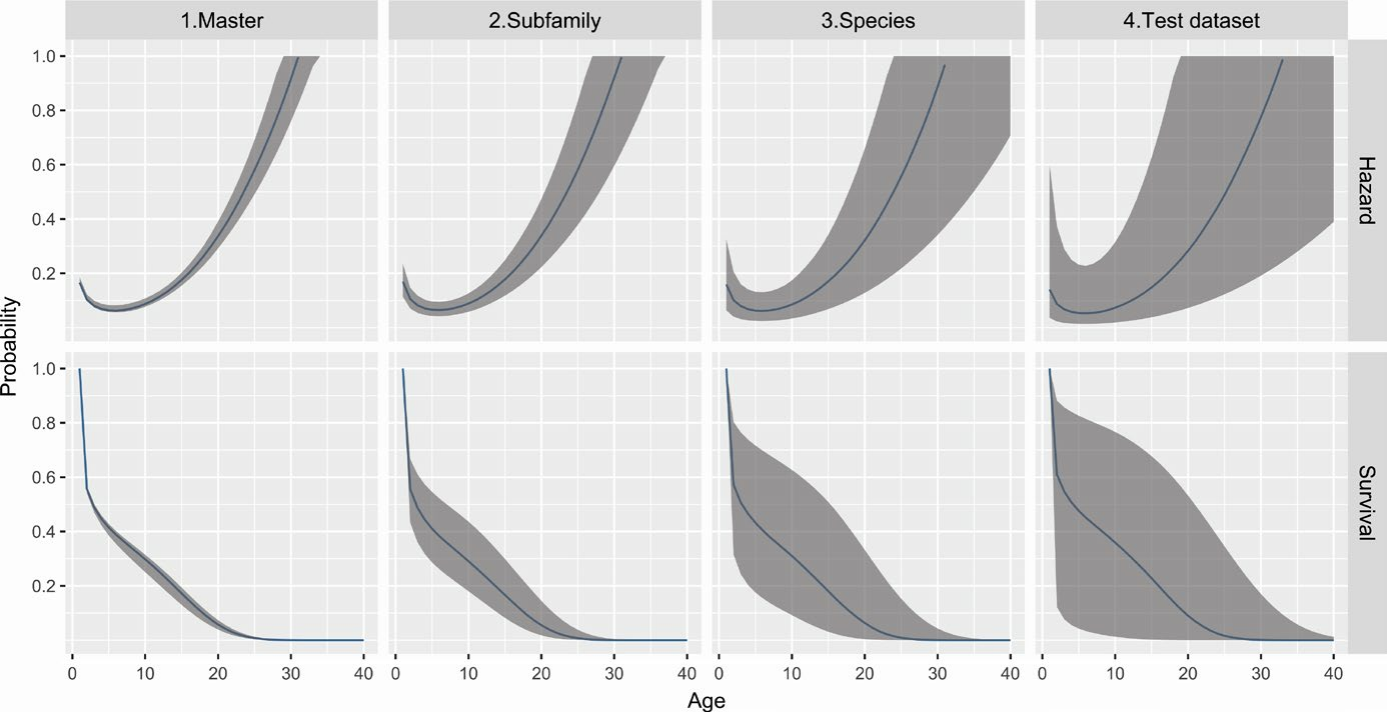
Hazard and survival functions (blue lines) and 95% credible intervals (shaded areas) using master‐, subfamily‐, species‐ (ribbon seal), and dataset (ribbon seal dataset)‐level estimates from model M3 fitted to 39 study datasets. The prior distribution for contemporary natural mortality of ribbon seals in the Sea of Okhotsk corresponds to the “test dataset” level (right panels) and includes a dataset‐specific variance component. As such it is less precise than the mean estimates for ribbon seals (third‐column panels) or for the mean survival of all seal species (left panels)

In addition to species‐specific estimates, we also estimated a natural mortality prior for a population of ribbon seals not subject to commercial harvesting. In particular, the prior distribution included simulation of a variance component at the “dataset” level, substantially decreasing precision from the mean natural mortality estimate for ribbon seals (Figure 5).

## 4 DISCUSSION

Life‐history parameters like natural mortality are of considerable interest to evolutionary and applied ecologists, but are often subject to considerable imprecision at the population level. In this study, we described a HBA framework for analyzing life‐history data gathered from multiple, independent studies. In particular, we showed how taxonomy‐based relationships can be embodied within alternative models for the relationship of life‐history parameters among datasets, doing so in a framework that also allows one to account for variance components attributable to phenotypic plasticity and observation error. This framework is useful for studying taxonomic variation in life‐history traits in that it allows one to infer general patterns from multiple, disparate datasets. However, one can also infer specific patterns from the general: for instance, one can borrow strength from other studies to estimate age‐specific survival for sparse datasets (the “Robin Hood effect”), and to construct prior distributions for populations where data are completely lacking (as in the ribbon seal example). Importantly, conducting inference in a hierarchical framework using survival and hazard functions (sensu Cox & Oakes, 1984) allowed us to combine data from multiple, disparate sources, and to estimate age‐specific survival functions even for those datasets where survival information was missing for a large proportion of age classes (Figures 2 and 5). We were also able to incorporate imprecise prior distributions for harvest rates to help separate natural and harvest mortality in hunted populations.

Our seal example illustrated how a parametric (in our case, RAW) model for natural mortality hazard rates could be used to induce the U‐shaped mortality curves that are expected of mammal populations (Caughley, 1966). In order to test whether this three‐parameter model was flexible enough to describe the shape of survival and hazard curves for different species (and particularly the effect of individual senescence), we also fitted an alternative, 5‐parameter Siler model to the same set of data and found that it produced very similar results. Thus, our results were robust to choice of survival model. This similarity of results, together with increased convergence issues with the Siler model, suggest that the RAW formulation may be a good starting place for future analyses.

Estimated variation in the parameters of the RAW model at different taxonomic and data‐related levels translated into different survivorship schedules among species and populations. Species with few mortality data had survivorship schedules that were shrunken towards the ensemble mean, while datasets with few data had survivorship schedules that were shrunken towards species‐specific means.

Our analysis indicated that variation in seal natural mortality could best be explained by species and dataset‐level effects. Although subfamily appeared in several competitive models, it clearly is not as useful a predictor as species was. This is perhaps not surprising, given the large amount of variation among species in morphology and natural history. Still, the subfamily variation was noteworthy because the four monachine species fell at or below the middle of the rank order for survival (Figure 4), suggesting a potential taxonomic basis for life‐history variation that may warrant further investigation. The dataset‐level variance component was present in most top models, indicating how important it was to control for population‐level and dataset‐specific factors. This variance component implicitly includes phenotypic plasticity as well as differences in methodology and observation error.

Our analysis produced some interesting relationships among species. In particular, it appeared that southern elephant seal had the lowest survival and earliest age of senescence (mortality rates started increasing around age 12–15). This is perhaps not very surprising, given the highly polygynous mating strategy and the tendency of adult males to fight for reproductive access. In the present analysis, we have treated males and females as different datasets; however, it would be interesting to separately estimate sex‐specific morality in future work given differences in male and female morphology and exposure to injuries.

Several enhancements to our modeling procedure would likely help to increase the precision of our mortality estimates, but would likely require richer datasets than we had access to in this paper. For instance, it would be useful to include greater realism in harvest modeling by using age‐ or time‐specific harvest rates. However, estimation of such rates would likely require large‐scale mark–recapture–recovery data or enough data to fit an integrated population model. Another useful improvement would be to account for permanent emigration. For instance, the models we have used assume that reported survival estimates represent true survival instead of apparent survival (true survival adjusted for permanent emigration), an assumption that is likely violated to some degree. For instance, some of the estimates in this paper came from mark–recapture studies, and it is well known that Cormack–Jolly–Seber mark–recapture models cannot differentiate mortality from permanent emigration. To reliably separate the two processes, additional information is needed such as hunter recoveries outside the study area (Burnham, 1993) or information about the spatial distribution of captures (Schaub & Royle, 2014). Permanent emigration might also be dealt with using an informative prior distribution if such data are missing.

Our approach in this study was to use a proportional hazards modeling framework to model changes in survivorship as a function of taxonomic and dataset‐level random effects. However, this approach is somewhat limiting, in that the overall shape of the hazard function is constrained to be similar among datasets. In our experience, such models were considerably more stable than ones that seek to model variation at the level of the RAW parameters (i.e., *a*,* b*, and *c*), but they can result in lack‐of‐fit when examining individual datasets. However, as one reviewer noted, the overall shape of the survival curve (e.g., Figure 4) differs from that reported for several well studied populations of Weddell seals and southern elephant seals, where there have been no documented senescent increases in mortality. When sufficient data are available to model senescence for a particular population or species, it may thus be preferable to base inference on a restricted set of data to prevent estimation of senescent effects that are artifacts from other species or datasets. Future research will be necessary to develop formulations that permit greater flexibility in the shape of hazard functions among datasets that are numerically tractable.

Although there is clearly room for improvements to our modeling framework in specific applications, we are optimistic that our general approach will be useful for other taxonomic groups and other life‐history parameters. For instance, a useful next step for seals would be to conduct similarly structured analyses for age‐at‐maturity, inter‐birth intervals, and number of offspring per adult female. Such analyses would be interesting in their own right, but would also help inform estimation of recruitment rates for population modeling exercises.

## 5 CONCLUSION

HBA provides a flexible framework for incorporating taxonomic structure into estimates of life‐history parameters while accounting for phenotypic plasticity and sampling artifacts. We have shown its utility in estimating age‐specific natural mortality from a collection of datasets, many of which were quite sparse, and for producing prior distributions for populations missing natural mortality data entirely. We suggest that evolutionary ecologists and conservation modelers consider application of such methods in their own research; leveraging the combined power of multiple datasets will generally lead to greater precision than considering each dataset individually.

## AUTHORS CONTRIBUTIONS

All authors conceived of the study; PBC formulated likelihoods and prior distributions; IST conducted the analysis; all authors drafted the manuscript.

## DATA ACCESSIBILITY

Code, data, and documentation to run the phocid seal analysis are available from the Dryad Digital Repository: https://doi.org/10.5061/dryad.qt6535q

## Supporting information


** **

** **
