# Supplementary material for: Taxonomy‐based hierarchical analysis of natural mortality: polar and subpolar phocid seals
Source: Ecol Evol. 2018 Oct 16;8(21):10530–41. doi: 10.1002/ece3.4522 (PMC6238133; doi:10.1002/ece3.4522)
Supplement: Supplementary file 1 [file ECE3-8-10530-s001.pdf]

Taxonomy-based hierarchical analysis of natural mortality: polar and sub-polar phocid seals.

Irina S. Trukhanova[1,2,]*, Paul B. Conn[2], and Peter L. Boveng[2]

[1]Polar Science Center, Applied Physics Laboratory, University of Washington, 1013 NE 40th St., Seattle, WA 98105 USA; [2]Marine Mammal Laboratory, Alaska Fisheries Science Center, NOAA National Marine Fisheries Service, 7600 Sand Point Way NE, Seattle, WA 98115 USA

∗irina_trukhanova@yahoo.com

Appendix S1: Supplementary tables and figures for hierarchical analysis of polar and sub-polar phocid seal natural mortality

Table S1. Complete list of datasets and studies used for analysis.

Dataset N	Study N	Subfamily	Species	Population	Sex	Sampling period	Type of data	Author
1	9	Phocinae	hooded	Newfoundland-Labrador	f	1964-1972	Cohort	Oritsland 1975
2	9	Phocinae	hooded	Newfoundland-Labrador	f	1964-1972	Cohort	Oritsland 1975
3	10	Phocinae	hooded	West Ice	f	1961-1965	Cohort	Jacobsen 1984
4	11	Phocinae	hooded	West Ice	f	1973-1977	Cohort	Jacobsen 1984
5	12	Phocinae	hooded	West Ice	f	1972-1978	Cohort	Jacobsen 1984
6	17	Phocinae	bearded	Bering Sea	f	1975-1983	Cohort	Burns, Frost 1983
7	17	Phocinae	bearded	Bering Sea	m	1975-1983	Cohort	Burns, Frost 1983
8	4	Phocinae	grey	Baltic Sea	f	2000-2004	Survival + total sample size	Kauhala 2012
9	4	Phocinae	grey	Baltic Sea	m	2000-2004	Survival + total sample size	Kauhala 2012
10	5	Phocinae	grey	Baltic Sea	f	2005-2009	Survival + total sample size	Kauhala 2012
11	5	Phocinae	grey	Baltic Sea	m	2005-2009	Survival + total sample size; Survival for	Kauhala 2012

© 2018 The Authors. Journal compilation © 2018 British Ecological Society, Ecology&Evolution

							age range + total sample size	
12	15	Phocinae	ribbon	Bering Sea	b	1985	Survival + sample size	Fedoseev 2000
13	18	Phocinae	ringed	Bothnian Bay	m	1973-1979	Survival + sample size	Helle et al. 1980
14	18	Phocinae	ringed	Bothnian Bay	f	1973-1979	Survival + sample size	Helle et al. 1980
15	20	Monachinae	weddell	Erebus Bay	f	1999	Survival + SE;	Hadley 2006
16	22	Monachinae	s_elephant	Macquarie Island	f	1961-1965	Survival + SE	Hindell 1991
17	22	Monachinae	s_elephant	Macquarie Island	m	1961-1965	Survival + SE	Hindell 1991
18	24	Monachinae	weddell	McMurdo Sound	f	1983-1998	Survival + SE; Survival for age range + SE	Cameron 2001
19	24	Monachinae	weddell	McMurdo Sound	m	1983-1998	Survival + SE; Survival for age range + SE	Cameron 2001
20	25	Monachinae	crabeater	Antarctic Peninsula	b	1964-1990	Survival + sample size; Survival for age range + sample size	Boveng 1993
21	14	Monachinae	n_elephant	California	f	1985–1987	Survival + CI	Condit et al. 2014
22	14	Monachinae	n_elephant	California	m	1985–1987	Survival + CI	Condit et al. 2014
23	16	Phocinae	spotted	Bering Sea	b	1967-1968	Survival; Survival for age range	Fedoseev 2000
24	13	Monachinae	s_elephant	South Georgia	m	1960s	Survival	Laws 1984
25	13	Monachinae	s_elephant	South Georgia	f	1960s	Survival	Laws 1984
26	1	Phocinae	grey	Sable Island	b	1985	Survival for age range + SE	Schwartz 2000
27	2	Phocinae	grey	Sable Island	b	1986	Survival for age range + SE	Schwartz 2000
28	3	Phocinae	grey	Sable Island	b	1987	Survival for age range + SE	Schwartz 2000

© 2018 The Authors. Journal compilation © 2018 British Ecological Society, Ecology&Evolution

29	6	Phocinae	harbor	Alaska	b	2000-2007	Survival for age range + SE	Hastings 2012
30	7	Phocinae	harbor	NE Scotland	f	2006-2011	Survival for age range + CI	Cordes 2014
31	7	Phocinae	harbor	NE Scotland	m	2006-2011	Survival for age range + CI	Cordes 2014
32	8	Phocinae	hooded	Newfoundland-Labrador	f	1964-1972	Survival for age range + CI	Oritsland 1975
33	8	Phocinae	hooded	Newfoundland-Labrador	m	1964-1972	Survival for age range + CI	Oritsland 1975
34	19	Phocinae	bearded	Svalbard-Barents	b	1968-1970	Survival for age range + sample size	Benjaminsen 1973
35	20	Monachinae	weddell	Erebus Bay	f	1999	Survival for age range + SE	Hadley 2006
36	21	Monachinae	weddell	McMurdo Sound	f	1966-1968	Survival for age range + SE	Stirling 1971
37	21	Monachinae	weddell	McMurdo Sound	m	1966-1968	Survival for age range + SE	Stirling 1971
38	23	Monachinae	weddell	McMurdoSound	f	1970-1982	Survival for age range + SE	Testa 1986
39	23	Monachinae	weddell	McMurdoSound	m	1970-1982	Survival for age range + SE	Testa 1986

© 2018 The Authors. Journal compilation © 2018 British Ecological Society, Ecology&Evolution

Table S2. Phocid seal harvest volumes and mean population abundances reported in the literature and used to estimate tentative harvest induced mortality rates for model priors.

Study	Max reported harvest rate (at the time of study)	Mean population estimate(at the time of study)	Proportion harvested	Comment	Sources
1	4533	394000	0.0115	Treated as one popul in Eastern Canada and US	Bowen 2016, DFO 2011
2	4533	394000	0.0115	Treated as one popul in Eastern Canada and US	Bowen 2016, DFO 2011
3	4533	394000	0.0115	Treated as one popul in Eastern Canada and US	Bowen 2016, DFO 2011
4	3300	46500	0.0710	Baltic Sea subpopulation	Härkönen 2016
5	3300	46500	0.0710	Baltic Sea subpopulation	Härkönen 2016
6	1197	375000	0.0032		Wolfe et al 2013
7	120	120000	0.0010	Used 0.001 as a value for 'small' number harvested	Harvey, Bowen 2016
8	23819	450000	0.0529	Data for 1970s	Kovacs 2016
9	23819	450000	0.0529	Data for 1970s	Kovacs 2016
10	23819	450000	0.0529		Stenson 1993
11	23819	450000	0.0529		Stenson 1993
12	23819	450000	0.0529		Stenson 1993
13	0	325000	0.0001	Population number - N of mature individuals. No harvest.	Hofmeyr 2015
14	0	224500	0.0001		Hückstädt 2015a
15	4750	95000	0.0500	Number not reported - used conservative 5% harvest rate.	Fedoseev 2000
16	4800	139000	0.0345	Number not reported - used conservative 5% harvest rate.	Fedoseev 2000
17	4000	87000	0.0460		Fedoseev 2000
18	NA	25000	0.0500		Helle et al 1980, Härkönen 2015
19	4000	87000	0.0460		Fedoseev 2000
20	633	633000	0.0010	Was not harvested commertially - 0.001 values used for 1960-1970s	Hückstädt 2015 b
21	633	633000	0.0010	Was not harvested commertially - 0.001 values used for 1960-1970s	Hückstädt 2015 b
22	0	325000	0.0001	Population number - N of mature individuals. No harvest.	Hofmeyr 2015
23	633	633000	0.0010	Was not harvested commertially - 0.001 values used for 1960-1970s	Hückstädt 2015 b
24	633	633000	0.0010	Was not harvested commertially - 0.001 values used for 1960-1970s	Hückstädt 2015 b
25	0	7000000	0.0001	No harvest.	Erickson et al 1971

© 2018 The Authors. Journal compilation © 2018 British Ecological Society, Ecology&Evolution

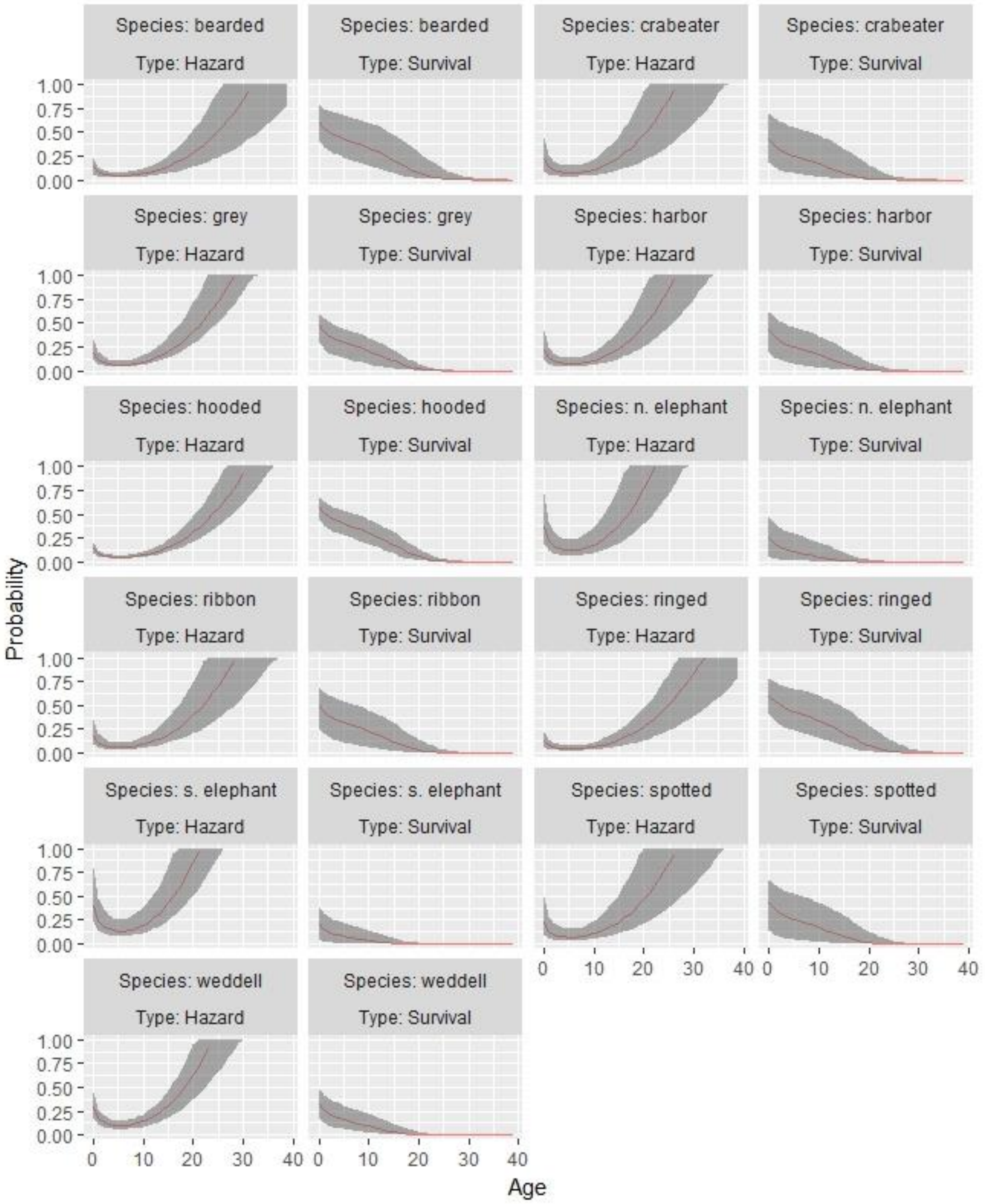

Fig. S1. Hazard and survival functions (red lines) and 95% credible intervals (shaded areas) as estimated using species level *a*, *b*, and *c* parameters obtained from fitting model M3 to 39 study datasets.

© 2018 The Authors. Journal compilation © 2018 British Ecological Society, Ecology&Evolution

REFERENCES

Benjaminsen, T. (1973). Age determination and the growth and age distribution from cementum growth layers of bearded seals at Svalbard. *FiskDir. Skr. Ser. HaaUnders. 16,* 159-170.

Boveng, P.L. (1993). *Variability in a crabeater seal population and the marine ecosystem near the Antarctic peninsula*. PhD thesis.

Bowen, D. (2016). *Halichoerus grypus*. The IUCN Red List of Threatened Species 2016: e.T9660A45226042. URL: http://dx.doi.org/10.2305/IUCN.UK.2016-1.RLTS.T9660A45226042.en. [accessed on 09 February 2017].

Burns, J.J. and Frost, K.J. (1979). *The Natural History and Ecology of the Bearded Seal*, *Erignathus barbatus.* Alaska department of Fish and Game. Final report.

Cameron, M. (2001). Dynamics of a Weddell seal (*Leptonychotes weddellii*) population in McMurdo Sound, Antarctica. *Journal of Chemical Information and Modeling*, *53*(9), 107. http://doi.org/10.1017/CBO9781107415324.004

Condit, R., Reiter, J., Morris, P. A., Berger, R., Allen, S. G., and Le Boeuf, B.J. (2014). Lifetime survival rates and senescence in northern elephant seals. *Marine Mammal Science*, *30*(1), 122–138. http://doi.org/10.1111/mms.12025

Cordes, L.S., & Thompson, P.M. (2014). Mark-recapture modeling accounting for state uncertainty provides concurrent estimates of survival and fecundity in a protected harbor seal population. *Marine Mammal Science*, *30*(2), 691–705. http://doi.org/10.1111/mms.12070

Erickson, A.W., Siniff, D.B., Cline, D.R. and Hofman, R.J. (1971). Distributional ecology of Antarctic seals. In: G. Deacon (ed.). Symposium on Antarctic Ice and Water Masses, *55-76*. Sci. Comm. Antarct Res., Cambridge, UK.

Fedoseev, G.A. (2000). *Population biology of ice-associated forms of seals and their role in the northern Pacific ecosystems.* Center for Russian Environmental Policy, Russian Marine Mammal Council; Moscow, Russia.

Hadley, G. L., Rotella, J. J., Garrott, R. A., and Nichols, J. D. (2006). Variation in probability of first reproduction of Weddell seals. *Journal of Animal Ecology*, *75*(5), 1058–1070. http://doi.org/10.1111/j.1365-2656.2006.01118.x

© 2018 The Authors. Journal compilation © 2018 British Ecological Society, Ecology&Evolution

Härkönen, T. (2015). *Pusa hispida ssp. botnica*. The IUCN Red List of Threatened Species 2015: e.T41673A66991604. http://dx.doi.org/10.2305/IUCN.UK.2015-4.RLTS.T41673A66991604.en. [accessed on 09 February 2017].

Härkönen, T. (2016). *Halichoerus grypus* (Baltic Sea subpopulation). The IUCN Red List of Threatened Species 2016: e.T74491261A74491289. URL: http://dx.doi.org/10.2305/IUCN.UK.2016-1.RLTS.T74491261A74491289.en. [accessed on 09 February 2017].

Harvey, J. and Bowen, D. (2016). *Phoca vitulina ssp. concolor*. The IUCN Red List of Threatened Species 2016: e.T17021A66991505. URL: http://dx.doi.org/10.2305/IUCN.UK.2016-1.RLTS.T17021A66991505.en. [accessed on 09 February 2017].

Hastings, K.K., Small, R.J. and Pendleton, G.W. (2012). Sex and age-specific survival of harbor seals (*Phoca vitulina*) from Tugidak Island, Alaska. *Journal of Mammalogy*, *93*(5), 1368–1379. http://doi.org/10.1644/11-MAMM-A-291.1

Helle, E. (1980). Age structure and sex ratio of the ringed seal *Phoca (Pusa) hispida* Schreber population in the Bothnian Bay, northern Baltic Sea. *Sonderdruck Aus Zeitschrift Für Säugetierkunde, 45*(310-317).

Hindell, M.A. (1991). Some Life-History Parameters of a Declining Population of Southern Elephant Seals, *Mirounga leonina*. *The Journal of Animal Ecology*, *60*(1), 119–134. http://doi.org/10.2307/5449

Hofmeyr, G.J.G. (2015). *Mirounga leonina*. The IUCN Red List of Threatened Species 2015: e.T13583A45227247. http://dx.doi.org/10.2305/IUCN.UK.2015-4.RLTS.T13583A45227247.en. [accessed on 09 February 2017].

Hückstädt, L. (2015a). *Mirounga angustirostris*. The IUCN Red List of Threatened Species 2015: e.T13581A45227116. URL: http://dx.doi.org/10.2305/IUCN.UK.2015-2.RLTS.T13581A45227116.en. [accessed on 09 February 2017].

Hückstädt, L. (2015b). *Leptonychotes weddellii*. The IUCN Red List of Threatened Species 2015: e.T11696A45226713. http://dx.doi.org/10.2305/IUCN.UK.2015-4.RLTS.T11696A45226713.en. [accessed on 09 February 2017].

Jacobsen, N.O. (1984). Estimates of pup production, age at first parturition and natural mortality for hooded seals in the west ice. *FiskDir. Skr. HavUnders.*, *17*, 483–498.

Kauhala, K., Ahola, M., and Kunnasranta, M. (2012). Demographic structure and mortality rate of a Baltic grey seal population at different stages of population change, judged on the basis of the

© 2018 The Authors. Journal compilation © 2018 British Ecological Society, Ecology&Evolution

hunting bag in Finland. *Annales Zoologici Fennici*, *2450* (November), 287–305. http://doi.org/10.5735/086.049.0502

Kovacs, K.M. (2016). *Cystophora cristata*. The IUCN Red List of Threatened Species 2016: e.T6204A45225150. URL: http://dx.doi.org/10.2305/IUCN.UK.2016-1.RLTS.T6204A45225150.en. [accessed on 09 February 2017].

Laws, R. M. (1984). *Antarctic Ecology*. *Seals*. Academic Press, London.

Oritsland, T., and Benjaminsen, T. (1975). Sex ration, Age composition and Mortality of Hooded Seals at Newfoundland. *ICNAF Research Bullitin*, *11*, 137–143.

Schwarz, C.J., and Stobo, W.T. (2000). Estimation of juvenile survival, adult survival, and age-specific pupping probabilities for the female grey seal (*Halichoerus gryprus*) on Sable Island from capture-recapture data. *Canadian Journal of Fisheries and Aquatic Sciences*, *57*(1994), 247–253. http://doi.org/10.1139/cjfas-57-1-247

Stenson, G.B. (1993). *The status of pinnipeds in the Newfoundland region*. NAFO SCR Doc. 93/34.

Stirling, I. 1971. Population dynamics of the Weddell seal (Leptonychotes weddelli) in McMurdo Sound, Antarctica, 1966-68. Pp. 141-161 in Antarctic Pinnipedia (W.H. Burt, ed.), Antarctic Research Series 18: 141-l61. American Geophysical Union, Washington, D. C.

Testa, J.W. (1986). Long term patterns in life history charactertstics and population dynamics of Weddell seals (*Leptonychotes weddelli*) in McMurdo Sound, Antarctica.

Wolfe, R.J., Bryant, J., Hutchinson-Scurbrough, L., Kookesh, M., Sill, L.A. (2013). *The Subsistence Harvest of Harbor Seals and Sea Lions in Southeast Alaska in 2012.* Technical paper N 383, Alaska Department of Fish and Game.

© 2018 The Authors. Journal compilation © 2018 British Ecological Society, Ecology&Evolution
