# Supplementary material for: Taxonomy‐based hierarchical analysis of natural mortality: polar and subpolar phocid seals
Source: Ecol Evol. 2018 Oct 16;8(21):10530–41. doi: 10.1002/ece3.4522 (PMC6238133; doi:10.1002/ece3.4522)
Supplement: Supplementary file 2 [file ECE3-8-10530-s002.pdf]

Taxonomy-based hierarchical analysis of natural mortality: polar and sub-polar phocid seals

Irina S. Trukhanova[1,2,*], Paul B. Conn[2], and Peter L. Boveng[2]

[1] Polar Science Center, Applied Physics Laboartory, University of Washington, 1013 NE 40th St., Seattle, WA 98105 USA; [2] Marine Mammal Laboratory, Alaska Fisheries Science Center, NOAA National Marine Fisheries Service, 7600 Sand Point Way NE, Seattle, WA 98115 USA

[*]irina_trukhanova@yahoo.com

Appendix S2: Methods for computing standard error of survival rates reported in literature when none are reported

Here we provide details on methods we used to come up with logit-scale precision of survival estimates when none were reported in the literature. These methods differ depending on the type of additional information provided (e.g., confidence interval, sample size). When no information on precision is provided, our goal is to come up with procedures that will allow us to use all survival information possible, while imposing a degree of conservatism by downweighting estimates that do not have accompanying estimates of precision. Our (perhaps naive) rationale is that authors that report precision are more likely to have used modern, reliable methods than those that do not.

Method 1: Confidence interval reported

If a confidence interval $(L_{t,d}, U_{t,d})$ is reported along with an age-specific estimate, $\tilde{S}_{t,d}$, we use the following procedure to calculate $\tau_{t,d}$:

1. Transform the lower and upper interval values ($L_{t,d}$ and $U_{t,d}$, respectively) to the logit scale:

$$\begin{aligned}\mathcal{L} &= \log(L_{t,d}(1-L_{t,d})^{-1}) \text{ and} \\ \mathcal{U} &= \log(U_{t,d}(1-U_{t,d})^{-1})\end{aligned}$$

2. If the survival estimate is also missing, calculate the logit-scale survival probability as $\text{logit}(\tilde{\phi}_{t,d}) = (\mathcal{U}+\mathcal{L})/2$.

3. Calculate precision (inverse variance) on the logit scale as

$$\tau_{t,d} = \left(\frac{2z}{\mathcal{U}+\mathcal{L}}\right)^2,$$

where z is the critical value of the standard normal distribution associated with the stated confidence level (e.g. $z = 1.96$ for a 95% interval).

Method 2: Sample size reported for each estimate

Sometimes survival probability will be reported for a given age along with a sample size (call this $n_{t,d}$), but with no other measure of precision. In these cases, we simply assumed that the number of individuals surviving an interval was binomially distributed, and used the binomial variance formula to produce

$$\tau_{t,d} = n_{t,d}(\tilde{\phi}_{t,d}(1-\tilde{\phi}_{t,d}))^{-1}.$$

This approximation is likely reasonable if survival/death can be determined without error, but may need revisiting if there are observation errors.

© 2018 The Authors. Journal compilation © 2018 British Ecological Society, *Ecology & Evolution*

Method 3: Sample size for entire study reported

When sample size is reported for an entire study (e.g. $n_d = 200$ animals), but survival estimates are provided for each age separately, we suggest using reported survival estimates to get an expected sample size for each age group, and then applying methods from the previous section. Specifically,

1. Calculate the expected proportion $\pi_{t,d}$ of each age group using eqn 4 from the main text (substituting $\tilde{\phi}_{t,d}$ for $\phi_{t,d}$),
2. Set $n_{t,d} = n_d \pi_{t,d}$, and
3. Calculate $\tau_{t,d}$ as in the previous section

Method 4: No measure of precision reported

In this case, we adopt a procedure based on the relationship between age and precision that are found in other studies. The procedure is conservative in that we set precision to a relatively imprecise level relative to what is observed in other datasets. Specifically, our procedure was as follows:

1. Determine coefficient of variation (CV) of survival estimates from other studies that have precision estimates from one of the methods above, i.e.

$$\hat{CV}(\tilde{\phi}_{t,d}) = \tilde{\phi}_{t,d}^{-1} \tau_{t,d}^{-0.5}$$

2. Use a generalized additive model (GAM; Wood 2006) to relate $\hat{CV}_{t,d}$ to t. The assumption is that precision will usually decrease in older age classes. For instance, we used the `mgcv` package (Wood 2011) in the R statisitical environment to fit such models to phocid seal data.
3. Use the fitted GAM to predict $CV_{t,d}$ as a function of t. Set $\hat{CV}_{t,d}$ equal to the upper 90th percentile of the prediction interval. The specific percentile is subjective, but our choice provides a reasonable degree of conservatism.
4. Set $\tau_{t,d} = (CV_{t,d} \tilde{\phi}_{t,d})^{-2}$

References

Wood, S.N. (2006) *Generalized additive models*. Chapman & Hall/CRC, Boca Raton, Florida.
Wood, S.N. (2011) Fast stable restricted maximum likelihood and marginal likelihood estimation of semiparametric generalized linear models. *Journal of the Royal Statistical Society: Series B (Statistical Methodology)*, **73**(1), 3–36.

© 2018 The Authors. Journal compilation © 2018 British Ecological Society, *Ecology & Evolution*
