# Supplementary material for: Taxonomy‐based hierarchical analysis of natural mortality: polar and subpolar phocid seals
Source: Ecol Evol. 2018 Oct 16;8(21):10530–41. doi: 10.1002/ece3.4522 (PMC6238133; doi:10.1002/ece3.4522)
Supplement: Supplementary file 3 [file ECE3-8-10530-s003.pdf]

Taxonomy-based hierarchical analysis of natural mortality: polar and sub-polar phocid seals.

Irina S. Trukhanova[1,2,*], Paul B. Conn[2], and Peter L. Boveng[2]

[1]Polar Science Center, Applied Physics Laboratory, University of Washington, 1013 NE 40th St., Seattle, WA 98105 USA; [2]Marine Mammal Laboratory, Alaska Fisheries Science Center, NOAA National Marine Fisheries Service, 7600 Sand Point Way NE, Seattle, WA 98115 USA

∗irina_trukhanova@yahoo.com

Appendix S3: Model M3 output

Table S1. Posterior means, standard deviations, and 95% credible intervals for all parameters from model M3.

	Mean	SD	%2.5	%97.5
Pi[1,1]	0.14802	0.00323	0.14162	0.15465
Pi[2,1]	0.17089	0.00755	0.15445	0.18450
Pi[3,1]	0.15954	0.00308	0.15352	0.16556
Pi[4,1]	0.15391	0.00346	0.14710	0.16081
Pi[5,1]	0.15503	0.00471	0.14587	0.16436
Pi[6,1]	0.08497	0.00530	0.07477	0.09528
Pi[7,1]	0.11296	0.00736	0.09860	0.12743
Pi[1,2]	0.12502	0.00215	0.12071	0.12934
Pi[2,2]	0.13880	0.00491	0.12744	0.14726
Pi[3,2]	0.13247	0.00195	0.12867	0.13622
Pi[4,2]	0.12889	0.00224	0.12446	0.13329
Pi[5,2]	0.12957	0.00304	0.12360	0.13545
Pi[6,2]	0.07782	0.00424	0.06954	0.08604
Pi[7,2]	0.09981	0.00557	0.08877	0.11050
Pi[1,3]	0.10713	0.00142	0.10429	0.10993
Pi[2,3]	0.11579	0.00297	0.10877	0.12074
Pi[3,3]	0.11197	0.00123	0.10957	0.11432
Pi[4,3]	0.10967	0.00144	0.10681	0.11247
Pi[5,3]	0.11010	0.00195	0.10619	0.11385
Pi[6,3]	0.07249	0.00346	0.06560	0.07907
Pi[7,3]	0.08933	0.00414	0.08104	0.09716
Pi[1,4]	0.09232	0.00089	0.09055	0.09406
Pi[2,4]	0.09761	0.00151	0.09407	0.10004
Pi[3,4]	0.09529	0.00071	0.09385	0.09665
Pi[4,4]	0.09390	0.00087	0.09212	0.09559
Pi[5,4]	0.09417	0.00118	0.09178	0.09636
Pi[6,4]	0.06798	0.00283	0.06223	0.07321
Pi[7,4]	0.08036	0.00297	0.07428	0.08585
Pi[1,5]	0.07973	0.00050	0.07870	0.08067
Pi[2,5]	0.08262	0.00047	0.08154	0.08336
Pi[3,5]	0.08131	0.00035	0.08057	0.08197
Pi[4,5]	0.08058	0.00046	0.07963	0.08146
Pi[5,5]	0.08073	0.00062	0.07944	0.08186
Pi[6,5]	0.06390	0.00231	0.05907	0.06811
Pi[7,5]	0.07243	0.00206	0.06823	0.07615

© 2018 The Authors. Journal compilation © 2018 British Ecological Society, Ecology&Evolution

Pi[1,6]	0.06887	0.00027	0.06831	0.06940
Pi[2,6]	0.06996	0.00052	0.06915	0.07119
Pi[3,6]	0.06939	0.00016	0.06908	0.06971
Pi[4,6]	0.06916	0.00020	0.06875	0.06957
Pi[5,6]	0.06923	0.00025	0.06872	0.06969
Pi[6,6]	0.06008	0.00189	0.05605	0.06350
Pi[7,6]	0.06530	0.00142	0.06226	0.06784
Pi[1,7]	0.05942	0.00026	0.05902	0.06007
Pi[2,7]	0.05912	0.00104	0.05746	0.06173
Pi[3,7]	0.05914	0.00024	0.05867	0.05962
Pi[4,7]	0.05930	0.00022	0.05890	0.05978
Pi[5,7]	0.05930	0.00027	0.05879	0.05991
Pi[6,7]	0.05642	0.00155	0.05308	0.05915
Pi[7,7]	0.05883	0.00111	0.05661	0.06094
Pi[1,8]	0.05117	0.00036	0.05055	0.05197
Pi[2,8]	0.04979	0.00139	0.04755	0.05326
Pi[3,8]	0.05029	0.00036	0.04957	0.05101
Pi[4,8]	0.05072	0.00036	0.05003	0.05147
Pi[5,8]	0.05067	0.00046	0.04972	0.05161
Pi[6,8]	0.05287	0.00130	0.05002	0.05506
Pi[7,8]	0.05290	0.00110	0.05107	0.05544
Pi[1,9]	0.04394	0.00046	0.04310	0.04497
Pi[2,9]	0.04172	0.00159	0.03912	0.04566
Pi[3,9]	0.04261	0.00046	0.04171	0.04350
Pi[4,9]	0.04326	0.00047	0.04235	0.04421
Pi[5,9]	0.04317	0.00062	0.04190	0.04436
Pi[6,9]	0.04938	0.00113	0.04688	0.05109
Pi[7,9]	0.04746	0.00124	0.04574	0.05047
Pi[1,10]	0.03761	0.00053	0.03661	0.03875
Pi[2,10]	0.03474	0.00166	0.03201	0.03875
Pi[3,10]	0.03596	0.00052	0.03496	0.03698
Pi[4,10]	0.03676	0.00055	0.03569	0.03786
Pi[5,10]	0.03664	0.00074	0.03515	0.03805
Pi[6,10]	0.04594	0.00103	0.04356	0.04741
Pi[7,10]	0.04246	0.00139	0.04042	0.04581
Pi[1,11]	0.03207	0.00057	0.03097	0.03325
Pi[2,11]	0.02873	0.00165	0.02600	0.03270
Pi[3,11]	0.03021	0.00055	0.02913	0.03128
Pi[4,11]	0.03111	0.00060	0.02994	0.03229
Pi[5,11]	0.03096	0.00081	0.02936	0.03251
Pi[6,11]	0.04256	0.00099	0.04020	0.04391
Pi[7,11]	0.03786	0.00151	0.03546	0.04136
Pi[1,12]	0.02722	0.00059	0.02608	0.02841
Pi[2,12]	0.02358	0.00156	0.02095	0.02731
Pi[3,12]	0.02524	0.00056	0.02416	0.02634
Pi[4,12]	0.02620	0.00062	0.02498	0.02742
Pi[5,12]	0.02603	0.00084	0.02438	0.02766
Pi[6,12]	0.03923	0.00100	0.03689	0.04068
Pi[7,12]	0.03364	0.00159	0.03097	0.03720
Pi[1,13]	0.02301	0.00059	0.02184	0.02418
Pi[2,13]	0.01918	0.00144	0.01674	0.02256
Pi[3,13]	0.02097	0.00056	0.01990	0.02206
Pi[4,13]	0.02196	0.00062	0.02071	0.02318
Pi[5,13]	0.02178	0.00085	0.02012	0.02343
Pi[6,13]	0.03597	0.00105	0.03364	0.03763
Pi[7,13]	0.02976	0.00162	0.02691	0.03333
Pi[1,14]	0.01934	0.00058	0.01819	0.02051
Pi[2,14]	0.01546	0.00129	0.01327	0.01838
Pi[3,14]	0.01732	0.00054	0.01628	0.01836
Pi[4,14]	0.01830	0.00061	0.01708	0.01950
Pi[5,14]	0.01811	0.00083	0.01649	0.01974
Pi[6,14]	0.03278	0.00111	0.03037	0.03471
Pi[7,14]	0.02621	0.00162	0.02330	0.02971
Pi[1,15]	0.01617	0.00056	0.01506	0.01728
Pi[2,15]	0.01233	0.00112	0.01037	0.01486
Pi[3,15]	0.01421	0.00051	0.01322	0.01520
Pi[4,15]	0.01516	0.00059	0.01400	0.01632
Pi[5,15]	0.01497	0.00080	0.01343	0.01654
Pi[6,15]	0.02969	0.00118	0.02720	0.03188
Pi[7,15]	0.02298	0.00160	0.02005	0.02639
Pi[1,16]	0.01345	0.00054	0.01237	0.01452
Pi[2,16]	0.00974	0.00096	0.00803	0.01184

© 2018 The Authors. Journal compilation © 2018 British Ecological Society, Ecology&Evolution

Pi[3,16]	0.01157	0.00047	0.01067	0.01250
Pi[4,16]	0.01248	0.00055	0.01138	0.01359
Pi[5,16]	0.01229	0.00076	0.01084	0.01379
Pi[6,16]	0.02672	0.00126	0.02414	0.02913
Pi[7,16]	0.02005	0.00155	0.01717	0.02329
Pi[1,17]	0.01111	0.00050	0.01010	0.01212
Pi[2,17]	0.00760	0.00081	0.00616	0.00933
Pi[3,17]	0.00936	0.00043	0.00853	0.01021
Pi[4,17]	0.01020	0.00052	0.00919	0.01125
Pi[5,17]	0.01003	0.00070	0.00869	0.01143
Pi[6,17]	0.02387	0.00133	0.02128	0.02646
Pi[7,17]	0.01740	0.00150	0.01458	0.02043
Pi[1,18]	0.00913	0.00047	0.00819	0.01006
Pi[2,18]	0.00587	0.00067	0.00466	0.00727
Pi[3,18]	0.00751	0.00039	0.00676	0.00827
Pi[4,18]	0.00829	0.00047	0.00736	0.00924
Pi[5,18]	0.00812	0.00064	0.00690	0.00941
Pi[6,18]	0.02118	0.00139	0.01852	0.02391
Pi[7,18]	0.01501	0.00145	0.01229	0.01791
Pi[1,19]	0.00745	0.00043	0.00659	0.00831
Pi[2,19]	0.00447	0.00055	0.00348	0.00560
Pi[3,19]	0.00597	0.00034	0.00532	0.00666
Pi[4,19]	0.00668	0.00043	0.00585	0.00754
Pi[5,19]	0.00652	0.00058	0.00543	0.00772
Pi[6,19]	0.01865	0.00144	0.01590	0.02149
Pi[7,19]	0.01289	0.00139	0.01027	0.01567
Pi[1,20]	0.00603	0.00040	0.00527	0.00683
Pi[2,20]	0.00337	0.00044	0.00257	0.00428
Pi[3,20]	0.00471	0.00030	0.00414	0.00532
Pi[4,20]	0.00535	0.00038	0.00461	0.00611
Pi[5,20]	0.00520	0.00052	0.00424	0.00628
Pi[6,20]	0.01629	0.00147	0.01348	0.01925
Pi[7,20]	0.01100	0.00133	0.00852	0.01367
Pi[1,21]	0.00486	0.00036	0.00416	0.00559
Pi[2,21]	0.00250	0.00036	0.00186	0.00324
Pi[3,21]	0.00369	0.00026	0.00319	0.00422

Pi[4,21]	0.00424	0.00034	0.00360	0.00493
Pi[5,21]	0.00412	0.00045	0.00327	0.00506
Pi[6,21]	0.01412	0.00148	0.01133	0.01715
Pi[7,21]	0.00933	0.00127	0.00698	0.01188
Pi[1,22]	0.00388	0.00032	0.00326	0.00453
Pi[2,22]	0.00183	0.00028	0.00132	0.00241
Pi[3,22]	0.00286	0.00022	0.00244	0.00331
Pi[4,22]	0.00334	0.00029	0.00278	0.00394
Pi[5,22]	0.00323	0.00040	0.00250	0.00405
Pi[6,22]	0.01214	0.00147	0.00941	0.01521
Pi[7,22]	0.00787	0.00120	0.00565	0.01032
Pi[1,23]	0.00307	0.00028	0.00254	0.00365
Pi[2,23]	0.00133	0.00022	0.00093	0.00178
Pi[3,23]	0.00220	0.00019	0.00184	0.00258
Pi[4,23]	0.00261	0.00025	0.00213	0.00314
Pi[5,23]	0.00251	0.00034	0.00190	0.00322
Pi[6,23]	0.01035	0.00145	0.00769	0.01341
Pi[7,23]	0.00659	0.00114	0.00451	0.00898
Pi[1,24]	0.00242	0.00025	0.00195	0.00294
Pi[2,24]	0.00095	0.00017	0.00064	0.00130
Pi[3,24]	0.00167	0.00016	0.00137	0.00200
Pi[4,24]	0.00202	0.00022	0.00162	0.00248
Pi[5,24]	0.00194	0.00029	0.00142	0.00255
Pi[6,24]	0.00875	0.00140	0.00624	0.01172
Pi[7,24]	0.00549	0.00107	0.00358	0.00773
Pi[1,25]	0.00189	0.00022	0.00148	0.00234
Pi[2,25]	0.00066	0.00013	0.00044	0.00095
Pi[3,25]	0.00126	0.00013	0.00102	0.00153
Pi[4,25]	0.00155	0.00018	0.00121	0.00194
Pi[5,25]	0.00148	0.00024	0.00106	0.00200
Pi[6,25]	0.00733	0.00134	0.00501	0.01022
Pi[7,25]	0.00455	0.00099	0.00279	0.00669
Pi[1,26]	0.00146	0.00018	0.00112	0.00185
Pi[2,26]	0.00046	0.00010	0.00029	0.00068
Pi[3,26]	0.00094	0.00011	0.00074	0.00116
Pi[4,26]	0.00118	0.00015	0.00090	0.00151

© 2018 The Authors. Journal compilation © 2018 British Ecological Society, Ecology&Evolution

Pi[5,26]	0.00112	0.00020	0.00077	0.00155	Pi[6,31]	0.00210	0.00079	0.00097	0.00399
Pi[6,26]	0.00609	0.00126	0.00394	0.00885	Pi[7,31]	0.00129	0.00054	0.00047	0.00251
Pi[7,26]	0.00374	0.00092	0.00214	0.00574	Pi[1,32]	0.00026	0.00006	0.00016	0.00039
Pi[1,27]	0.00112	0.00016	0.00083	0.00145	Pi[2,32]	0.00004	0.00001	0.00002	0.00007
Pi[2,27]	0.00031	0.00008	0.00019	0.00048	Pi[3,32]	0.00013	0.00002	0.00009	0.00018
Pi[3,27]	0.00069	0.00009	0.00053	0.00087	Pi[4,32]	0.00019	0.00004	0.00012	0.00028
Pi[4,27]	0.00089	0.00013	0.00066	0.00116	Pi[5,32]	0.00017	0.00005	0.00009	0.00029
Pi[5,27]	0.00084	0.00016	0.00056	0.00120	Pi[6,32]	0.00165	0.00069	0.00070	0.00336
Pi[6,27]	0.00501	0.00118	0.00306	0.00758	Pi[7,32]	0.00102	0.00047	0.00034	0.00212
Pi[7,27]	0.00306	0.00084	0.00164	0.00490	Pi[1,33]	0.00019	0.00005	0.00011	0.00029
Pi[1,28]	0.00085	0.00013	0.00062	0.00113	Pi[2,33]	0.00002	0.00001	0.00001	0.00005
Pi[2,28]	0.00021	0.00006	0.00012	0.00034	Pi[3,33]	0.00009	0.00002	0.00006	0.00013
Pi[3,28]	0.00051	0.00007	0.00038	0.00065	Pi[4,33]	0.00013	0.00003	0.00008	0.00020
Pi[4,28]	0.00066	0.00010	0.00048	0.00089	Pi[5,33]	0.00012	0.00004	0.00006	0.00021
Pi[5,28]	0.00062	0.00013	0.00040	0.00092	Pi[6,33]	0.00129	0.00060	0.00050	0.00279
Pi[6,28]	0.00409	0.00108	0.00235	0.00651	Pi[7,33]	0.00081	0.00041	0.00023	0.00178
Pi[7,28]	0.00249	0.00076	0.00123	0.00418	Pi[1,34]	0.00014	0.00004	0.00008	0.00022
Pi[1,29]	0.00064	0.00011	0.00045	0.00088	Pi[2,34]	0.00001	0.00001	0.00000	0.00003
Pi[2,29]	0.00014	0.00004	0.00007	0.00023	Pi[3,34]	0.00006	0.00001	0.00004	0.00009
Pi[3,29]	0.00037	0.00005	0.00027	0.00048	Pi[4,34]	0.00009	0.00002	0.00005	0.00015
Pi[4,29]	0.00049	0.00008	0.00034	0.00067	Pi[5,34]	0.00008	0.00003	0.00004	0.00015
Pi[5,29]	0.00046	0.00011	0.00028	0.00070	Pi[6,34]	0.00100	0.00052	0.00035	0.00230
Pi[6,29]	0.00330	0.00099	0.00177	0.00555	Pi[7,34]	0.00063	0.00035	0.00016	0.00147
Pi[7,29]	0.00201	0.00068	0.00090	0.00354	Pi[1,35]	0.00010	0.00003	0.00005	0.00016
Pi[1,30]	0.00048	0.00009	0.00032	0.00068	Pi[2,35]	0.00001	0.00000	0.00000	0.00002
Pi[2,30]	0.00009	0.00003	0.00005	0.00016	Pi[3,35]	0.00004	0.00001	0.00002	0.00006
Pi[3,30]	0.00026	0.00004	0.00019	0.00035	Pi[4,35]	0.00006	0.00002	0.00004	0.00011
Pi[4,30]	0.00036	0.00007	0.00024	0.00051	Pi[5,35]	0.00006	0.00002	0.00003	0.00011
Pi[5,30]	0.00033	0.00008	0.00020	0.00052	Pi[6,35]	0.00076	0.00044	0.00024	0.00188
Pi[6,30]	0.00265	0.00089	0.00132	0.00472	Pi[7,35]	0.00049	0.00030	0.00011	0.00122
Pi[7,30]	0.00161	0.00061	0.00065	0.00298	Pi[1,36]	0.00007	0.00002	0.00003	0.00012
Pi[1,31]	0.00036	0.00007	0.00023	0.00052	Pi[2,36]	0.00000	0.00000	0.00000	0.00001
Pi[2,31]	0.00006	0.00002	0.00003	0.00011	Pi[3,36]	0.00003	0.00001	0.00002	0.00004
Pi[3,31]	0.00018	0.00003	0.00013	0.00025	Pi[4,36]	0.00004	0.00001	0.00002	0.00008
Pi[4,31]	0.00026	0.00005	0.00017	0.00038	Pi[5,36]	0.00004	0.00002	0.00002	0.00008
Pi[5,31]	0.00024	0.00007	0.00013	0.00039	Pi[6,36]	0.00058	0.00037	0.00016	0.00152

© 2018 The Authors. Journal compilation © 2018 British Ecological Society, Ecology&Evolution

Pi[7,36]	0.00038	0.00025	0.00007	0.00102	S_combo[25,2]	0.69922	0.02526	0.64990	0.74675
S_combo[8,1]	0.79802	0.00876	0.78064	0.81486	S_combo[8,3]	0.94485	0.00123	0.94237	0.94736
S_combo[9,1]	0.72973	0.04066	0.65346	0.80941	S_combo[9,3]	0.89419	0.00415	0.88597	0.90223
S_combo[10,1]	0.75297	0.01650	0.72313	0.78699	S_combo[10,3]	0.92187	0.00164	0.91867	0.92501
S_combo[11,1]	0.51971	0.01688	0.48602	0.55257	S_combo[11,3]	0.84914	0.00336	0.84240	0.85595
S_combo[12,1]	0.58954	0.02984	0.52572	0.64313	S_combo[12,3]	0.81756	0.00709	0.80315	0.83101
S_combo[13,1]	0.77846	0.05524	0.65508	0.87250	S_combo[13,3]	0.90861	0.02196	0.86227	0.94669
S_combo[14,1]	0.82136	0.04433	0.72149	0.89577	S_combo[14,3]	0.93010	0.02090	0.88164	0.96332
S_combo[15,1]	0.67179	0.04875	0.56966	0.75884	S_combo[15,3]	0.90492	0.01619	0.86975	0.93291
S_combo[16,1]	0.34375	0.01836	0.30741	0.37935	S_combo[16,3]	0.76708	0.00572	0.75529	0.77789
S_combo[17,1]	0.41856	0.01836	0.38265	0.45398	S_combo[17,3]	0.80555	0.00490	0.79587	0.81492
S_combo[18,1]	0.70144	0.03121	0.64010	0.75902	S_combo[18,3]	0.91497	0.00951	0.89537	0.93217
S_combo[19,1]	0.67912	0.03408	0.60711	0.74179	S_combo[19,3]	0.90762	0.01071	0.88465	0.92671
S_combo[20,1]	0.82975	0.00869	0.81243	0.84677	S_combo[20,3]	0.95467	0.00233	0.95020	0.95923
S_combo[21,1]	0.51474	0.02355	0.46637	0.55982	S_combo[21,3]	0.84795	0.00742	0.83320	0.86206
S_combo[22,1]	0.23846	0.02221	0.19617	0.28270	S_combo[22,3]	0.70018	0.01227	0.67551	0.72350
S_combo[23,1]	0.41162	0.06318	0.29042	0.53561	S_combo[23,3]	0.78231	0.03020	0.72186	0.83940
S_combo[24,1]	0.27392	0.04120	0.19576	0.35748	S_combo[24,3]	0.72365	0.02622	0.66879	0.77214
S_combo[25,1]	0.33098	0.03921	0.25794	0.40954	S_combo[25,3]	0.75909	0.02097	0.71794	0.79842
S_combo[8,2]	0.92926	0.00184	0.92553	0.93301	S_combo[8,4]	0.95099	0.00106	0.94886	0.95304
S_combo[9,2]	0.87635	0.00658	0.86315	0.88915	S_combo[9,4]	0.90123	0.00438	0.89249	0.90930
S_combo[10,2]	0.90365	0.00264	0.89856	0.90881	S_combo[10,4]	0.92905	0.00172	0.92562	0.93232
S_combo[11,2]	0.80902	0.00485	0.79922	0.81847	S_combo[11,4]	0.86528	0.00293	0.85938	0.87103
S_combo[12,2]	0.79157	0.00938	0.77278	0.80930	S_combo[12,4]	0.82790	0.00644	0.81488	0.84014
S_combo[13,2]	0.89472	0.02363	0.84314	0.93592	S_combo[13,4]	0.91408	0.02163	0.86832	0.95135
S_combo[14,2]	0.91869	0.02189	0.86859	0.95453	S_combo[14,4]	0.93458	0.02071	0.88608	0.96680
S_combo[15,2]	0.87863	0.02042	0.83423	0.91398	S_combo[15,4]	0.91536	0.01450	0.88366	0.94032
S_combo[16,2]	0.70869	0.00762	0.69330	0.72312	S_combo[16,4]	0.79107	0.00515	0.78029	0.80066
S_combo[17,2]	0.75518	0.00665	0.74217	0.76779	S_combo[17,4]	0.82603	0.00436	0.81731	0.83454
S_combo[18,2]	0.89128	0.01214	0.86657	0.91350	S_combo[18,4]	0.92435	0.00846	0.90697	0.93976
S_combo[19,2]	0.88200	0.01361	0.85314	0.90608	S_combo[19,4]	0.91779	0.00955	0.89711	0.93468
S_combo[20,2]	0.94156	0.00295	0.93587	0.94727	S_combo[20,4]	0.95982	0.00211	0.95566	0.96397
S_combo[21,2]	0.80721	0.00959	0.78800	0.82550	S_combo[21,4]	0.86435	0.00657	0.85128	0.87710
S_combo[22,2]	0.62951	0.01494	0.59965	0.65878	S_combo[22,4]	0.72977	0.01121	0.70794	0.75117
S_combo[23,2]	0.73387	0.03603	0.65935	0.80043	S_combo[23,4]	0.80204	0.02788	0.74642	0.85528
S_combo[24,2]	0.65720	0.03095	0.59340	0.71472	S_combo[24,4]	0.75131	0.02409	0.70046	0.79561

© 2018 The Authors. Journal compilation © 2018 British Ecological Society, Ecology&Evolution

S_combo[25,4]	0.78376	0.01912	0.74589	0.81971
S_combo[8,5]	0.95329	0.00101	0.95125	0.95526
S_combo[9,5]	0.90387	0.00465	0.89471	0.91245
S_combo[10,5]	0.93175	0.00180	0.92818	0.93522
S_combo[11,5]	0.87139	0.00285	0.86572	0.87703
S_combo[12,5]	0.83180	0.00625	0.81904	0.84393
S_combo[13,5]	0.91613	0.02156	0.87067	0.95320
S_combo[14,5]	0.93626	0.02067	0.88787	0.96816
S_combo[15,5]	0.91929	0.01387	0.88894	0.94334
S_combo[16,5]	0.80023	0.00507	0.78991	0.80974
S_combo[17,5]	0.83382	0.00425	0.82513	0.84204
S_combo[18,5]	0.92789	0.00806	0.91133	0.94255
S_combo[19,5]	0.92162	0.00910	0.90202	0.93777
S_combo[20,5]	0.96175	0.00204	0.95774	0.96577
S_combo[21,5]	0.87056	0.00626	0.85777	0.88272
S_combo[22,5]	0.74115	0.01087	0.71996	0.76257
S_combo[23,5]	0.80954	0.02703	0.75585	0.86127
S_combo[24,5]	0.76192	0.02324	0.71267	0.80431
S_combo[25,5]	0.79319	0.01840	0.75624	0.82777
S_combo[8,6]	0.95348	0.00102	0.95146	0.95546
S_combo[9,6]	0.90408	0.00467	0.89485	0.91273
S_combo[10,6]	0.93195	0.00178	0.92851	0.93535
S_combo[11,6]	0.87188	0.00291	0.86612	0.87757
S_combo[12,6]	0.83211	0.00624	0.81937	0.84441
S_combo[13,6]	0.91630	0.02155	0.87086	0.95331
S_combo[14,6]	0.93639	0.02067	0.88809	0.96820
S_combo[15,6]	0.91961	0.01385	0.88919	0.94365
S_combo[16,6]	0.80097	0.00520	0.79043	0.81076
S_combo[17,6]	0.83445	0.00434	0.82561	0.84278
S_combo[18,6]	0.92817	0.00801	0.91172	0.94261
S_combo[19,6]	0.92193	0.00906	0.90245	0.93800
S_combo[20,6]	0.96190	0.00205	0.95789	0.96592
S_combo[21,6]	0.87106	0.00622	0.85850	0.88306
S_combo[22,6]	0.74207	0.01092	0.72054	0.76352
S_combo[23,6]	0.81015	0.02697	0.75680	0.86186
S_combo[24,6]	0.76278	0.02314	0.71353	0.80520
S_combo[25,6]	0.79395	0.01834	0.75749	0.82864
S_combo[8,7]	0.95228	0.00104	0.95022	0.95431
S_combo[9,7]	0.90269	0.00450	0.89388	0.91089
S_combo[10,7]	0.93054	0.00171	0.92724	0.93381
S_combo[11,7]	0.86869	0.00303	0.86276	0.87461
S_combo[12,7]	0.83007	0.00634	0.81703	0.84253
S_combo[13,7]	0.91523	0.02158	0.86952	0.95239
S_combo[14,7]	0.93552	0.02070	0.88696	0.96748
S_combo[15,7]	0.91755	0.01421	0.88608	0.94233
S_combo[16,7]	0.79618	0.00544	0.78534	0.80654
S_combo[17,7]	0.83038	0.00452	0.82140	0.83905
S_combo[18,7]	0.92633	0.00820	0.90951	0.94095
S_combo[19,7]	0.91994	0.00927	0.90006	0.93637
S_combo[20,7]	0.96090	0.00208	0.95679	0.96503
S_combo[21,7]	0.86782	0.00635	0.85486	0.88018
S_combo[22,7]	0.73611	0.01120	0.71400	0.75851
S_combo[23,7]	0.80623	0.02741	0.75196	0.85880
S_combo[24,7]	0.75724	0.02352	0.70709	0.80053
S_combo[25,7]	0.78903	0.01869	0.75234	0.82489
S_combo[8,8]	0.95007	0.00109	0.94792	0.95220
S_combo[9,8]	0.90014	0.00428	0.89155	0.90814
S_combo[10,8]	0.92795	0.00163	0.92476	0.93116
S_combo[11,8]	0.86285	0.00320	0.85653	0.86910
S_combo[12,8]	0.82635	0.00654	0.81301	0.83909
S_combo[13,8]	0.91326	0.02166	0.86719	0.95058
S_combo[14,8]	0.93391	0.02074	0.88535	0.96620
S_combo[15,8]	0.91378	0.01485	0.88104	0.93964
S_combo[16,8]	0.78745	0.00574	0.77585	0.79854
S_combo[17,8]	0.82295	0.00476	0.81366	0.83227
S_combo[18,8]	0.92296	0.00855	0.90539	0.93824
S_combo[19,8]	0.91628	0.00966	0.89548	0.93344
S_combo[20,8]	0.95905	0.00215	0.95486	0.96328
S_combo[21,8]	0.86189	0.00660	0.84838	0.87471
S_combo[22,8]	0.72528	0.01163	0.70274	0.74845
S_combo[23,8]	0.79908	0.02823	0.74282	0.85336
S_combo[24,8]	0.74714	0.02422	0.69549	0.79143

© 2018 The Authors. Journal compilation © 2018 British Ecological Society, Ecology&Evolution

S_combo[25,8]	0.78004	0.01933	0.74178	0.81718	S_combo[25,10]	0.75359	0.02118	0.71198	0.79377
S_combo[8,9]	0.94709	0.00115	0.94483	0.94932	S_combo[8,11]	0.93929	0.00135	0.93657	0.94189
S_combo[9,9]	0.89670	0.00416	0.88821	0.90465	S_combo[9,11]	0.88775	0.00479	0.87807	0.89691
S_combo[10,9]	0.92444	0.00163	0.92123	0.92756	S_combo[10,11]	0.91531	0.00204	0.91121	0.91910
S_combo[11,9]	0.85499	0.00341	0.84832	0.86160	S_combo[11,11]	0.83468	0.00399	0.82689	0.84256
S_combo[12,9]	0.82131	0.00685	0.80716	0.83436	S_combo[12,11]	0.80824	0.00783	0.79249	0.82294
S_combo[13,9]	0.91060	0.02180	0.86417	0.94833	S_combo[13,11]	0.90367	0.02238	0.85523	0.94250
S_combo[14,9]	0.93173	0.02083	0.88329	0.96462	S_combo[14,11]	0.92604	0.02119	0.87699	0.96014
S_combo[15,9]	0.90869	0.01570	0.87415	0.93606	S_combo[15,11]	0.89547	0.01789	0.85633	0.92633
S_combo[16,9]	0.77573	0.00609	0.76362	0.78729	S_combo[16,11]	0.74582	0.00698	0.73191	0.75915
S_combo[17,9]	0.81295	0.00505	0.80320	0.82278	S_combo[17,11]	0.78730	0.00582	0.77609	0.79850
S_combo[18,9]	0.91839	0.00903	0.89993	0.93465	S_combo[18,11]	0.90651	0.01028	0.88553	0.92500
S_combo[19,9]	0.91133	0.01020	0.88927	0.92947	S_combo[19,11]	0.89846	0.01161	0.87393	0.91920
S_combo[20,9]	0.95654	0.00222	0.95224	0.96092	S_combo[20,11]	0.95001	0.00240	0.94535	0.95473
S_combo[21,9]	0.85390	0.00694	0.83959	0.86730	S_combo[21,11]	0.83328	0.00784	0.81720	0.84844
S_combo[22,9]	0.71082	0.01218	0.68710	0.73453	S_combo[22,11]	0.67424	0.01352	0.64778	0.70064
S_combo[23,9]	0.78946	0.02933	0.72978	0.84531	S_combo[23,11]	0.76478	0.03218	0.69904	0.82558
S_combo[24,9]	0.73364	0.02516	0.68063	0.77999	S_combo[24,11]	0.69938	0.02745	0.64157	0.75007
S_combo[25,9]	0.76801	0.02018	0.72834	0.80654	S_combo[25,11]	0.73730	0.02229	0.69324	0.77995
S_combo[8,10]	0.94346	0.00123	0.94099	0.94583	S_combo[8,12]	0.93466	0.00150	0.93168	0.93758
S_combo[9,10]	0.89253	0.00429	0.88396	0.90073	S_combo[9,12]	0.88243	0.00564	0.87091	0.89336
S_combo[10,10]	0.92019	0.00175	0.91670	0.92340	S_combo[10,12]	0.90989	0.00246	0.90497	0.91460
S_combo[11,10]	0.84550	0.00367	0.83831	0.85260	S_combo[11,12]	0.82275	0.00438	0.81425	0.83133
S_combo[12,10]	0.81521	0.00728	0.80033	0.82892	S_combo[12,12]	0.80051	0.00851	0.78360	0.81661
S_combo[13,10]	0.90737	0.02203	0.86049	0.94592	S_combo[13,12]	0.89954	0.02286	0.85001	0.93916
S_combo[14,10]	0.92908	0.02098	0.88044	0.96244	S_combo[14,12]	0.92265	0.02148	0.87374	0.95753
S_combo[15,10]	0.90253	0.01673	0.86613	0.93142	S_combo[15,12]	0.88765	0.01916	0.84614	0.92051
S_combo[16,10]	0.76170	0.00650	0.74868	0.77416	S_combo[16,12]	0.72846	0.00753	0.71351	0.74272
S_combo[17,10]	0.80094	0.00540	0.79049	0.81143	S_combo[17,12]	0.77232	0.00631	0.76014	0.78428
S_combo[18,10]	0.91285	0.00961	0.89319	0.93010	S_combo[18,12]	0.89947	0.01102	0.87685	0.91946
S_combo[19,10]	0.90533	0.01086	0.88212	0.92470	S_combo[19,12]	0.89084	0.01244	0.86444	0.91296
S_combo[20,10]	0.95350	0.00230	0.94905	0.95802	S_combo[20,12]	0.94611	0.00250	0.94123	0.95102
S_combo[21,10]	0.84426	0.00736	0.82911	0.85844	S_combo[21,12]	0.82117	0.00840	0.80394	0.83742
S_combo[22,10]	0.69359	0.01281	0.66862	0.71843	S_combo[22,12]	0.65323	0.01429	0.62524	0.68089
S_combo[23,10]	0.77790	0.03066	0.71584	0.83552	S_combo[23,12]	0.75039	0.03386	0.68086	0.81384
S_combo[24,10]	0.71753	0.02625	0.66229	0.76584	S_combo[24,12]	0.67963	0.02871	0.61955	0.73311

© 2018 The Authors. Journal compilation © 2018 British Ecological Society, Ecology&Evolution

S_combo[25,12]	0.71950	0.02348	0.67371	0.76455
S_combo[8,13]	0.92960	0.00169	0.92627	0.93294
S_combo[9,13]	0.87665	0.00679	0.86293	0.88950
S_combo[10,13]	0.90398	0.00299	0.89802	0.90974
S_combo[11,13]	0.80988	0.00484	0.80040	0.81942
S_combo[12,13]	0.79213	0.00930	0.77361	0.80958
S_combo[13,13]	0.89505	0.02350	0.84396	0.93604
S_combo[14,13]	0.91895	0.02187	0.86929	0.95488
S_combo[15,13]	0.87916	0.02053	0.83464	0.91456
S_combo[16,13]	0.70991	0.00816	0.69413	0.72544
S_combo[17,13]	0.75625	0.00688	0.74265	0.76923
S_combo[18,13]	0.89182	0.01183	0.86735	0.91310
S_combo[19,13]	0.88257	0.01335	0.85432	0.90634
S_combo[20,13]	0.94187	0.00261	0.93680	0.94698
S_combo[21,13]	0.80810	0.00902	0.78974	0.82545
S_combo[22,13]	0.63098	0.01511	0.60130	0.66057
S_combo[23,13]	0.73494	0.03565	0.66190	0.80151
S_combo[24,13]	0.65865	0.03001	0.59673	0.71461
S_combo[25,13]	0.70051	0.02473	0.65221	0.74757
S_combo[8,14]	0.92417	0.00192	0.92037	0.92795
S_combo[9,14]	0.87045	0.00816	0.85416	0.88542
S_combo[10,14]	0.89765	0.00360	0.89051	0.90478
S_combo[11,14]	0.79621	0.00539	0.78572	0.80706
S_combo[12,14]	0.78318	0.01019	0.76282	0.80235
S_combo[13,14]	0.89022	0.02429	0.83734	0.93302
S_combo[14,14]	0.91498	0.02236	0.86357	0.95206
S_combo[15,14]	0.87009	0.02199	0.82189	0.90799
S_combo[16,14]	0.69041	0.00886	0.67325	0.70751
S_combo[17,14]	0.73926	0.00752	0.72423	0.75346
S_combo[18,14]	0.88363	0.01270	0.85713	0.90656
S_combo[19,14]	0.87373	0.01432	0.84368	0.89915
S_combo[20,14]	0.93730	0.00273	0.93187	0.94265
S_combo[21,14]	0.79423	0.00971	0.77434	0.81247
S_combo[22,14]	0.60780	0.01598	0.57602	0.63860
S_combo[23,14]	0.71862	0.03753	0.64236	0.78879
S_combo[24,14]	0.63672	0.03132	0.57213	0.69599
S_combo[25,14]	0.68056	0.02601	0.62938	0.73033
S_combo[8,15]	0.91841	0.00219	0.91413	0.92274
S_combo[9,15]	0.86387	0.00970	0.84451	0.88183
S_combo[10,15]	0.89093	0.00428	0.88258	0.89950
S_combo[11,15]	0.78184	0.00602	0.77013	0.79398
S_combo[12,15]	0.77373	0.01117	0.75144	0.79469
S_combo[13,15]	0.88510	0.02524	0.82939	0.92930
S_combo[14,15]	0.91075	0.02295	0.85944	0.94904
S_combo[15,15]	0.86050	0.02351	0.80934	0.90117
S_combo[16,15]	0.67016	0.00964	0.65140	0.68894
S_combo[17,15]	0.72153	0.00825	0.70500	0.73697
S_combo[18,15]	0.87496	0.01362	0.84668	0.89964
S_combo[19,15]	0.86438	0.01535	0.83209	0.89158
S_combo[20,15]	0.93245	0.00286	0.92681	0.93810
S_combo[21,15]	0.77966	0.01046	0.75808	0.79952
S_combo[22,15]	0.58397	0.01687	0.55024	0.61671
S_combo[23,15]	0.70160	0.03946	0.62223	0.77613
S_combo[24,15]	0.61411	0.03261	0.54689	0.67552
S_combo[25,15]	0.65988	0.02731	0.60593	0.71221
S_combo[8,16]	0.91233	0.00250	0.90750	0.91732
S_combo[9,16]	0.85696	0.01138	0.83424	0.87812
S_combo[10,16]	0.88387	0.00501	0.87422	0.89395
S_combo[11,16]	0.76690	0.00673	0.75388	0.78039
S_combo[12,16]	0.76384	0.01223	0.73944	0.78672
S_combo[13,16]	0.87970	0.02635	0.82093	0.92582
S_combo[14,16]	0.90629	0.02366	0.85350	0.94569
S_combo[15,16]	0.85045	0.02509	0.79555	0.89377
S_combo[16,16]	0.64933	0.01049	0.62924	0.66976
S_combo[17,16]	0.70319	0.00906	0.68519	0.72028
S_combo[18,16]	0.86586	0.01458	0.83547	0.89239
S_combo[19,16]	0.85457	0.01643	0.81995	0.88383
S_combo[20,16]	0.92732	0.00301	0.92142	0.93322
S_combo[21,16]	0.76450	0.01126	0.74134	0.78571
S_combo[22,16]	0.55973	0.01779	0.52413	0.59405
S_combo[23,16]	0.68400	0.04144	0.59991	0.76203
S_combo[24,16]	0.59101	0.03387	0.52219	0.65451

© 2018 The Authors. Journal compilation © 2018 British Ecological Society, Ecology&Evolution

S_combo[25,16]	0.63863	0.02861	0.58186	0.69350	S_combo[25,18]	0.59509	0.03116	0.53314	0.65463
S_combo[8,17]	0.90597	0.00286	0.90055	0.91167	S_combo[8,19]	0.89248	0.00369	0.88540	0.89988
S_combo[9,17]	0.84974	0.01316	0.82366	0.87444	S_combo[9,19]	0.83448	0.01696	0.80088	0.86643
S_combo[10,17]	0.87648	0.00579	0.86540	0.88813	S_combo[10,19]	0.86085	0.00745	0.84644	0.87581
S_combo[11,17]	0.75145	0.00751	0.73689	0.76637	S_combo[11,19]	0.71937	0.00926	0.70134	0.73797
S_combo[12,17]	0.75354	0.01336	0.72676	0.77844	S_combo[12,19]	0.73195	0.01578	0.69962	0.76121
S_combo[13,17]	0.87406	0.02762	0.81242	0.92175	S_combo[13,19]	0.86209	0.03057	0.79205	0.91354
S_combo[14,17]	0.90162	0.02448	0.84699	0.94216	S_combo[14,19]	0.89169	0.02643	0.83270	0.93565
S_combo[15,17]	0.83999	0.02672	0.78207	0.88639	S_combo[15,19]	0.81800	0.03009	0.75233	0.87039
S_combo[16,17]	0.62808	0.01140	0.60606	0.65005	S_combo[16,19]	0.58483	0.01336	0.55963	0.61061
S_combo[17,17]	0.68436	0.00993	0.66440	0.70285	S_combo[17,19]	0.64568	0.01184	0.62190	0.66778
S_combo[18,17]	0.85637	0.01560	0.82396	0.88476	S_combo[18,19]	0.83638	0.01773	0.80013	0.86844
S_combo[19,17]	0.84435	0.01756	0.80702	0.87561	S_combo[19,19]	0.82285	0.01993	0.77998	0.85874
S_combo[20,17]	0.92196	0.00318	0.91578	0.92810	S_combo[20,19]	0.91055	0.00359	0.90347	0.91748
S_combo[21,17]	0.74885	0.01213	0.72386	0.77163	S_combo[21,19]	0.71635	0.01399	0.68834	0.74222
S_combo[22,17]	0.53526	0.01872	0.49800	0.57142	S_combo[22,19]	0.48637	0.02054	0.44569	0.52591
S_combo[23,17]	0.66594	0.04342	0.57794	0.74826	S_combo[23,19]	0.62886	0.04732	0.53304	0.71805
S_combo[24,17]	0.56761	0.03509	0.49589	0.63275	S_combo[24,19]	0.52055	0.03730	0.44394	0.59094
S_combo[25,17]	0.61699	0.02990	0.55746	0.67467	S_combo[25,19]	0.57304	0.03238	0.50924	0.63480
S_combo[8,18]	0.89935	0.00326	0.89307	0.90591	S_combo[8,20]	0.88538	0.00416	0.87749	0.89371
S_combo[9,18]	0.84224	0.01502	0.81237	0.87030	S_combo[9,20]	0.82648	0.01895	0.78910	0.86204
S_combo[10,18]	0.86880	0.00660	0.85606	0.88205	S_combo[10,20]	0.85265	0.00833	0.83656	0.86940
S_combo[11,18]	0.73559	0.00836	0.71938	0.75246	S_combo[11,20]	0.70287	0.01021	0.68301	0.72366
S_combo[12,18]	0.74290	0.01455	0.71343	0.76990	S_combo[12,20]	0.72071	0.01705	0.68523	0.75219
S_combo[13,18]	0.86818	0.02903	0.80239	0.91804	S_combo[13,20]	0.85580	0.03224	0.78188	0.90910
S_combo[14,18]	0.89675	0.02540	0.83971	0.93892	S_combo[14,20]	0.88646	0.02757	0.82508	0.93197
S_combo[15,18]	0.82916	0.02839	0.76751	0.87876	S_combo[15,20]	0.80655	0.03182	0.73675	0.86209
S_combo[16,18]	0.60654	0.01236	0.58277	0.63032	S_combo[16,20]	0.56306	0.01438	0.53589	0.59118
S_combo[17,18]	0.66516	0.01086	0.64337	0.68549	S_combo[17,20]	0.62600	0.01286	0.60007	0.65032
S_combo[18,18]	0.84653	0.01665	0.81222	0.87677	S_combo[18,20]	0.82594	0.01885	0.78712	0.86008
S_combo[19,18]	0.83376	0.01872	0.79363	0.86759	S_combo[19,20]	0.81164	0.02116	0.76645	0.84999
S_combo[20,18]	0.91636	0.00338	0.90983	0.92286	S_combo[20,20]	0.90454	0.00383	0.89684	0.91186
S_combo[21,18]	0.73278	0.01304	0.70628	0.75717	S_combo[21,20]	0.69965	0.01498	0.66977	0.72743
S_combo[22,18]	0.51076	0.01964	0.47161	0.54889	S_combo[22,20]	0.46222	0.02140	0.41955	0.50391
S_combo[23,18]	0.64753	0.04539	0.55620	0.73350	S_combo[23,20]	0.61002	0.04921	0.51051	0.70245
S_combo[24,18]	0.54408	0.03623	0.46989	0.61152	S_combo[24,20]	0.49715	0.03828	0.41877	0.56996

© 2018 The Authors. Journal compilation © 2018 British Ecological Society, Ecology&Evolution

S_combo[25,20]	0.55097	0.03355	0.48462	0.61493	S_combo[25,22]	0.50713	0.03569	0.43743	0.57502
S_combo[8,21]	0.87808	0.00467	0.86918	0.88739	S_combo[8,23]	0.86289	0.00577	0.85172	0.87424
S_combo[9,21]	0.81826	0.02099	0.77720	0.85787	S_combo[9,23]	0.80124	0.02519	0.75230	0.84917
S_combo[10,21]	0.84422	0.00924	0.82636	0.86281	S_combo[10,23]	0.82675	0.01113	0.80529	0.84898
S_combo[11,21]	0.68614	0.01121	0.66432	0.70881	S_combo[11,23]	0.65221	0.01330	0.62630	0.67870
S_combo[12,21]	0.70924	0.01835	0.67108	0.74309	S_combo[12,23]	0.68568	0.02101	0.64153	0.72420
S_combo[13,21]	0.84933	0.03401	0.77154	0.90580	S_combo[13,23]	0.83588	0.03785	0.74793	0.89887
S_combo[14,21]	0.88107	0.02879	0.81580	0.92887	S_combo[14,23]	0.86983	0.03151	0.79843	0.92130
S_combo[15,21]	0.79483	0.03356	0.72130	0.85371	S_combo[15,23]	0.77073	0.03707	0.68926	0.83591
S_combo[16,21]	0.54131	0.01541	0.51219	0.57168	S_combo[16,23]	0.49825	0.01746	0.46549	0.53300
S_combo[17,21]	0.60621	0.01390	0.57835	0.63300	S_combo[17,23]	0.56658	0.01604	0.53474	0.59764
S_combo[18,21]	0.81524	0.02000	0.77367	0.85139	S_combo[18,23]	0.79316	0.02236	0.74643	0.83381
S_combo[19,21]	0.80017	0.02242	0.75230	0.84072	S_combo[19,23]	0.77655	0.02500	0.72381	0.82188
S_combo[20,21]	0.89835	0.00410	0.89013	0.90609	S_combo[20,23]	0.88544	0.00472	0.87610	0.89450
S_combo[21,21]	0.68272	0.01600	0.65082	0.71230	S_combo[21,23]	0.64841	0.01811	0.61243	0.68167
S_combo[22,21]	0.43843	0.02223	0.39402	0.48155	S_combo[22,23]	0.39228	0.02370	0.34560	0.43876
S_combo[23,21]	0.59107	0.05104	0.48824	0.68697	S_combo[23,23]	0.55314	0.05444	0.44455	0.65650
S_combo[24,21]	0.47399	0.03916	0.39460	0.54826	S_combo[24,23]	0.42874	0.04061	0.34749	0.50602
S_combo[25,21]	0.52897	0.03466	0.46079	0.59488	S_combo[25,23]	0.48551	0.03665	0.41441	0.55524
S_combo[8,22]	0.87057	0.00520	0.86057	0.88092	S_combo[8,24]	0.85503	0.00637	0.84274	0.86746
S_combo[9,22]	0.80984	0.02308	0.76500	0.85359	S_combo[9,24]	0.79248	0.02733	0.73941	0.84449
S_combo[10,22]	0.83559	0.01017	0.81601	0.85600	S_combo[10,24]	0.81774	0.01210	0.79428	0.84187
S_combo[11,22]	0.66924	0.01224	0.64556	0.69392	S_combo[11,24]	0.63511	0.01438	0.60708	0.66383
S_combo[12,22]	0.69755	0.01967	0.65639	0.73399	S_combo[12,24]	0.67366	0.02235	0.62660	0.71422
S_combo[13,22]	0.84268	0.03589	0.75971	0.90246	S_combo[13,24]	0.82893	0.03989	0.73720	0.89558
S_combo[14,22]	0.87552	0.03011	0.80720	0.92513	S_combo[14,24]	0.86401	0.03298	0.78928	0.91731
S_combo[15,22]	0.78288	0.03531	0.70548	0.84500	S_combo[15,24]	0.75841	0.03883	0.67346	0.82669
S_combo[16,22]	0.51969	0.01644	0.48856	0.55248	S_combo[16,24]	0.47708	0.01845	0.44246	0.51366
S_combo[17,22]	0.58638	0.01497	0.55655	0.61529	S_combo[17,24]	0.54685	0.01711	0.51318	0.58022
S_combo[18,22]	0.80431	0.02117	0.76016	0.84279	S_combo[18,24]	0.78184	0.02356	0.73246	0.82492
S_combo[19,22]	0.78847	0.02370	0.73785	0.83131	S_combo[19,24]	0.76446	0.02631	0.70915	0.81224
S_combo[20,22]	0.89197	0.00440	0.88321	0.90033	S_combo[20,24]	0.87874	0.00507	0.86851	0.88846
S_combo[21,22]	0.66563	0.01705	0.63145	0.69682	S_combo[21,24]	0.63113	0.01918	0.59288	0.66665
S_combo[22,22]	0.41509	0.02299	0.36918	0.45993	S_combo[22,24]	0.37009	0.02432	0.32245	0.41785
S_combo[23,22]	0.57209	0.05278	0.46666	0.67209	S_combo[23,24]	0.53427	0.05599	0.42297	0.64052
S_combo[24,22]	0.45116	0.03994	0.37043	0.52700	S_combo[24,24]	0.40679	0.04116	0.32497	0.48542

© 2018 The Authors. Journal compilation © 2018 British Ecological Society, Ecology&Evolution

S_combo[25,24]	0.46420	0.03753	0.39152	0.53659
S_combo[8,25]	0.84701	0.00700	0.83351	0.86057
S_combo[9,25]	0.78356	0.02949	0.72635	0.84002
S_combo[10,25]	0.80857	0.01309	0.78305	0.83460
S_combo[11,25]	0.61796	0.01546	0.58774	0.64880
S_combo[12,25]	0.66149	0.02371	0.61128	0.70433
S_combo[13,25]	0.82185	0.04200	0.72536	0.89257
S_combo[14,25]	0.85805	0.03452	0.77900	0.91447
S_combo[15,25]	0.74593	0.04058	0.65693	0.81752
S_combo[16,25]	0.45622	0.01941	0.41976	0.49471
S_combo[17,25]	0.52727	0.01816	0.49133	0.56260
S_combo[18,25]	0.77034	0.02478	0.71846	0.81595
S_combo[19,25]	0.75220	0.02763	0.69369	0.80255
S_combo[20,25]	0.87191	0.00545	0.86090	0.88242
S_combo[21,25]	0.61381	0.02026	0.57352	0.65117
S_combo[22,25]	0.34855	0.02487	0.30033	0.39777
S_combo[23,25]	0.51554	0.05744	0.40126	0.62477
S_combo[24,25]	0.38539	0.04159	0.30221	0.46536
S_combo[25,25]	0.44324	0.03831	0.36897	0.51785
S_combo[8,26]	0.83885	0.00766	0.82391	0.85362
S_combo[9,26]	0.77450	0.03167	0.71262	0.83528
S_combo[10,26]	0.79924	0.01409	0.77164	0.82711
S_combo[11,26]	0.60082	0.01656	0.56827	0.63404
S_combo[12,26]	0.64922	0.02506	0.59651	0.69453
S_combo[13,26]	0.81463	0.04416	0.71404	0.88901
S_combo[14,26]	0.85198	0.03612	0.76882	0.91165
S_combo[15,26]	0.73332	0.04232	0.64046	0.80832
S_combo[16,26]	0.43573	0.02033	0.39739	0.47605
S_combo[17,26]	0.50786	0.01920	0.46964	0.54521
S_combo[18,26]	0.75871	0.02601	0.70390	0.80626
S_combo[19,26]	0.73981	0.02896	0.67894	0.79303
S_combo[20,26]	0.86493	0.00585	0.85311	0.87629
S_combo[21,26]	0.59651	0.02134	0.55402	0.63596
S_combo[22,26]	0.32773	0.02533	0.27913	0.37827
S_combo[23,26]	0.49699	0.05877	0.38046	0.60947
S_combo[24,26]	0.36458	0.04190	0.28170	0.44622

S_combo[25,26]	0.42270	0.03899	0.34697	0.49906
S_combo[8,27]	0.83055	0.00834	0.81428	0.84662
S_combo[9,27]	0.76532	0.03385	0.69941	0.83053
S_combo[10,27]	0.78978	0.01511	0.76029	0.81936
S_combo[11,27]	0.58372	0.01765	0.54921	0.61912
S_combo[12,27]	0.63687	0.02640	0.58132	0.68444
S_combo[13,27]	0.80730	0.04637	0.70178	0.88632
S_combo[14,27]	0.84580	0.03778	0.75926	0.90837
S_combo[15,27]	0.72060	0.04404	0.62346	0.79900
S_combo[16,27]	0.41565	0.02120	0.37532	0.45763
S_combo[17,27]	0.48868	0.02021	0.44854	0.52821
S_combo[18,27]	0.74694	0.02725	0.68974	0.79692
S_combo[19,27]	0.72730	0.03029	0.66422	0.78306
S_combo[20,27]	0.85782	0.00628	0.84515	0.86998
S_combo[21,27]	0.57926	0.02240	0.53473	0.62059
S_combo[22,27]	0.30766	0.02570	0.25845	0.35921
S_combo[23,27]	0.47866	0.05998	0.35975	0.59370
S_combo[24,27]	0.34440	0.04209	0.26123	0.42729
S_combo[25,27]	0.40261	0.03958	0.32570	0.48059
S_combo[8,28]	0.82212	0.00904	0.80435	0.83957
S_combo[9,28]	0.75602	0.03604	0.68627	0.82587
S_combo[10,28]	0.78020	0.01614	0.74890	0.81182
S_combo[11,28]	0.56668	0.01873	0.52970	0.60408
S_combo[12,28]	0.62444	0.02774	0.56602	0.67425
S_combo[13,28]	0.79987	0.04862	0.68947	0.88235
S_combo[14,28]	0.83950	0.03948	0.74862	0.90453
S_combo[15,28]	0.70779	0.04573	0.60641	0.78971
S_combo[16,28]	0.39603	0.02201	0.35405	0.43942
S_combo[17,28]	0.46977	0.02118	0.42760	0.51126
S_combo[18,28]	0.73507	0.02849	0.67576	0.78735
S_combo[19,28]	0.71469	0.03161	0.64946	0.77300
S_combo[20,28]	0.85060	0.00674	0.83695	0.86366
S_combo[21,28]	0.56208	0.02345	0.51559	0.60540
S_combo[22,28]	0.28836	0.02598	0.23850	0.34039
S_combo[23,28]	0.46059	0.06106	0.34032	0.57811
S_combo[24,28]	0.32488	0.04216	0.24185	0.40877

© 2018 The Authors. Journal compilation © 2018 British Ecological Society, Ecology&Evolution

S_combo[25,28]	0.38301	0.04007	0.30528	0.46241
S_combo[8,29]	0.81357	0.00977	0.79427	0.83234
S_combo[9,29]	0.74662	0.03823	0.67287	0.82093
S_combo[10,29]	0.77050	0.01718	0.73718	0.80410
S_combo[11,29]	0.54975	0.01980	0.51051	0.58940
S_combo[12,29]	0.61197	0.02906	0.55080	0.66408
S_combo[13,29]	0.79233	0.05090	0.67818	0.87869
S_combo[14,29]	0.83311	0.04123	0.73700	0.90063
S_combo[15,29]	0.69491	0.04741	0.59002	0.77998
S_combo[16,29]	0.37690	0.02276	0.33301	0.42193
S_combo[17,29]	0.45117	0.02211	0.40722	0.49423
S_combo[18,29]	0.72310	0.02973	0.66137	0.77777
S_combo[19,29]	0.70200	0.03293	0.63367	0.76263
S_combo[20,29]	0.84326	0.00722	0.82867	0.85723
S_combo[21,29]	0.54502	0.02448	0.49682	0.59034
S_combo[22,29]	0.26986	0.02617	0.21950	0.32227
S_combo[23,29]	0.44281	0.06201	0.32109	0.56285
S_combo[24,29]	0.30605	0.04211	0.22342	0.39052
S_combo[25,29]	0.36394	0.04045	0.28610	0.44453
S_combo[8,30]	0.80492	0.01052	0.78412	0.82514
S_combo[9,30]	0.73713	0.04042	0.65951	0.81586
S_combo[10,30]	0.76070	0.01822	0.72527	0.79635
S_combo[11,30]	0.53295	0.02085	0.49136	0.57482
S_combo[12,30]	0.59946	0.03036	0.53522	0.65370
S_combo[13,30]	0.78470	0.05321	0.66637	0.87503
S_combo[14,30]	0.82663	0.04302	0.72598	0.89741
S_combo[15,30]	0.68199	0.04905	0.57381	0.77015
S_combo[16,30]	0.35829	0.02344	0.31295	0.40482
S_combo[17,30]	0.43289	0.02299	0.38738	0.47772
S_combo[18,30]	0.71106	0.03097	0.64694	0.76773
S_combo[19,30]	0.68926	0.03424	0.61803	0.75198
S_combo[20,30]	0.83581	0.00772	0.82026	0.85063
S_combo[21,30]	0.52810	0.02549	0.47783	0.57527
S_combo[22,30]	0.25216	0.02626	0.20155	0.30483
S_combo[23,30]	0.42536	0.06284	0.30252	0.54771
S_combo[24,30]	0.28793	0.04195	0.20601	0.37253
S_combo[25,30]	0.34542	0.04073	0.26745	0.42671
S_combo[8,31]	0.79616	0.01129	0.77368	0.81786
S_combo[9,31]	0.72756	0.04260	0.64575	0.81075
S_combo[10,31]	0.75081	0.01927	0.71318	0.78851
S_combo[11,31]	0.51631	0.02187	0.47282	0.56013
S_combo[12,31]	0.58695	0.03164	0.52021	0.64361
S_combo[13,31]	0.77698	0.05553	0.65358	0.87143
S_combo[14,31]	0.82006	0.04484	0.71493	0.89384
S_combo[15,31]	0.66903	0.05066	0.55765	0.76055
S_combo[16,31]	0.34021	0.02405	0.29369	0.38812
S_combo[17,31]	0.41498	0.02382	0.36798	0.46137
S_combo[18,31]	0.69896	0.03220	0.63227	0.75814
S_combo[19,31]	0.67647	0.03554	0.60305	0.74253
S_combo[20,31]	0.82826	0.00824	0.81166	0.84397
S_combo[21,31]	0.51134	0.02646	0.45919	0.56039
S_combo[22,31]	0.23529	0.02626	0.18467	0.28836
S_combo[23,31]	0.40824	0.06353	0.28436	0.53264
S_combo[24,31]	0.27053	0.04168	0.18975	0.35539
S_combo[25,31]	0.32747	0.04090	0.24985	0.40920
S_combo[8,32]	0.78732	0.01207	0.76309	0.81049
S_combo[9,32]	0.71792	0.04476	0.63187	0.80557
S_combo[10,32]	0.74084	0.02032	0.70085	0.78040
S_combo[11,32]	0.49985	0.02286	0.45455	0.54555
S_combo[12,32]	0.57444	0.03289	0.50505	0.63327
S_combo[13,32]	0.76919	0.05787	0.64162	0.86747
S_combo[14,32]	0.81340	0.04668	0.70410	0.89047
S_combo[15,32]	0.65605	0.05223	0.54120	0.75025
S_combo[16,32]	0.32270	0.02458	0.27507	0.37166
S_combo[17,32]	0.39745	0.02459	0.34924	0.44543
S_combo[18,32]	0.68681	0.03342	0.61765	0.74843
S_combo[19,32]	0.66366	0.03682	0.58774	0.73178
S_combo[20,32]	0.82062	0.00879	0.80279	0.83728
S_combo[21,32]	0.49478	0.02740	0.44108	0.54587
S_combo[22,32]	0.21922	0.02617	0.16899	0.27247
S_combo[23,32]	0.39150	0.06409	0.26688	0.51770
S_combo[24,32]	0.25386	0.04131	0.17473	0.33821

© 2018 The Authors. Journal compilation © 2018 British Ecological Society, Ecology&Evolution

S_combo[25,32]	0.31012	0.04097	0.23332	0.39259	S_combo[25,34]	0.27723	0.04082	0.20132	0.36006
S_combo[8,33]	0.77839	0.01287	0.75260	0.80271	S_combo[8,35]	0.76031	0.01451	0.73135	0.78773
S_combo[9,33]	0.70822	0.04691	0.61735	0.80030	S_combo[9,35]	0.68869	0.05115	0.59017	0.78951
S_combo[10,33]	0.73080	0.02138	0.68889	0.77219	S_combo[10,35]	0.71055	0.02348	0.66486	0.75605
S_combo[11,33]	0.48359	0.02382	0.43616	0.53103	S_combo[11,35]	0.45176	0.02563	0.40124	0.50260
S_combo[12,33]	0.56195	0.03412	0.49000	0.62313	S_combo[12,35]	0.53709	0.03646	0.46082	0.60254
S_combo[13,33]	0.76133	0.06022	0.62806	0.86404	S_combo[13,35]	0.74543	0.06491	0.60473	0.85644
S_combo[14,33]	0.80668	0.04855	0.69440	0.88640	S_combo[14,35]	0.79301	0.05235	0.67181	0.87996
S_combo[15,33]	0.64307	0.05376	0.52487	0.74008	S_combo[15,35]	0.61717	0.05668	0.49371	0.72000
S_combo[16,33]	0.30576	0.02504	0.25717	0.35532	S_combo[16,35]	0.27364	0.02572	0.22427	0.32452
S_combo[17,33]	0.38033	0.02531	0.33077	0.42992	S_combo[17,35]	0.34737	0.02654	0.29582	0.39957
S_combo[18,33]	0.67463	0.03463	0.60259	0.73847	S_combo[18,35]	0.65023	0.03701	0.57349	0.71871
S_combo[19,33]	0.65083	0.03808	0.57245	0.72125	S_combo[19,35]	0.62520	0.04054	0.54220	0.70115
S_combo[20,33]	0.81289	0.00935	0.79391	0.83053	S_combo[20,35]	0.79720	0.01053	0.77606	0.81720
S_combo[21,33]	0.47843	0.02830	0.42338	0.53135	S_combo[21,35]	0.44646	0.02996	0.38820	0.50271
S_combo[22,33]	0.20397	0.02600	0.15428	0.25716	S_combo[22,35]	0.17585	0.02541	0.12789	0.22820
S_combo[23,33]	0.37514	0.06452	0.25011	0.50289	S_combo[23,35]	0.34364	0.06499	0.21871	0.47339
S_combo[24,33]	0.23792	0.04084	0.16029	0.32121	S_combo[24,35]	0.20823	0.03962	0.13474	0.28986
S_combo[25,33]	0.29337	0.04095	0.21728	0.37606	S_combo[25,35]	0.26171	0.04061	0.18625	0.34458
S_combo[8,34]	0.76939	0.01368	0.74197	0.79540	S_combo[8,36]	0.75118	0.01535	0.72053	0.78012
S_combo[9,34]	0.69848	0.04905	0.60395	0.79500	S_combo[9,36]	0.67887	0.05324	0.57610	0.78397
S_combo[10,34]	0.72070	0.02243	0.67690	0.76427	S_combo[10,36]	0.70036	0.02453	0.65274	0.74763
S_combo[11,34]	0.46755	0.02474	0.41861	0.51664	S_combo[11,36]	0.43622	0.02646	0.38439	0.48888
S_combo[12,34]	0.54949	0.03531	0.47544	0.61279	S_combo[12,36]	0.52474	0.03758	0.44632	0.59221
S_combo[13,34]	0.75341	0.06257	0.61652	0.86071	S_combo[13,36]	0.73740	0.06726	0.59030	0.85205
S_combo[14,34]	0.79988	0.05044	0.68358	0.88320	S_combo[14,36]	0.78608	0.05427	0.66104	0.87638
S_combo[15,34]	0.63011	0.05524	0.50894	0.73018	S_combo[15,36]	0.60427	0.05807	0.47858	0.71003
S_combo[16,34]	0.28940	0.02542	0.24058	0.33968	S_combo[16,36]	0.25847	0.02595	0.20901	0.30982
S_combo[17,34]	0.36363	0.02595	0.31272	0.41459	S_combo[17,36]	0.33156	0.02705	0.27871	0.38511
S_combo[18,34]	0.66244	0.03583	0.58769	0.72862	S_combo[18,36]	0.63804	0.03817	0.55979	0.70879
S_combo[19,34]	0.63801	0.03932	0.55715	0.71143	S_combo[19,36]	0.61242	0.04172	0.52756	0.69053
S_combo[20,34]	0.80509	0.00994	0.78485	0.82389	S_combo[20,36]	0.78925	0.01115	0.76701	0.81038
S_combo[21,34]	0.46232	0.02915	0.40572	0.51690	S_combo[21,36]	0.43086	0.03072	0.37113	0.48880
S_combo[22,34]	0.18952	0.02575	0.14057	0.24211	S_combo[22,36]	0.16295	0.02501	0.11608	0.21480
S_combo[23,34]	0.35918	0.06482	0.23422	0.48809	S_combo[23,36]	0.32852	0.06505	0.20475	0.45923
S_combo[24,34]	0.22271	0.04027	0.14709	0.30493	S_combo[24,36]	0.19446	0.03888	0.12323	0.27529

© 2018 The Authors. Journal compilation © 2018 British Ecological Society, Ecology&Evolution

S_combo[25,36]	0.24681	0.04030	0.17201	0.32969
S_numerator[26,2]	0.56768	0.05407	0.46002	0.66906
S_numerator[27,2]	0.40617	0.04854	0.31190	0.50128
S_numerator[28,2]	0.20414	0.02741	0.15361	0.25902
S_numerator[32,2]	0.43584	0.05510	0.32700	0.54118
S_numerator[42,2]	0.41261	0.04782	0.31895	0.50590
S_numerator[43,2]	0.22183	0.03881	0.14861	0.30096
S_numerator[44,2]	0.37511	0.02497	0.32685	0.42261
S_numerator[45,2]	0.37511	0.02497	0.32685	0.42261
S_numerator[46,2]	0.37511	0.02497	0.32685	0.42261
S_numerator[47,2]	0.37511	0.02497	0.32685	0.42261
S_numerator[48,2]	0.37511	0.02497	0.32685	0.42261
S_numerator[49,2]	0.37511	0.02497	0.32685	0.42261
S_numerator[50,2]	0.37511	0.02497	0.32685	0.42261
S_numerator[51,2]	0.37511	0.02497	0.32685	0.42261
S_numerator[52,2]	0.37511	0.02497	0.32685	0.42261
S_numerator[53,2]	0.37511	0.02497	0.32685	0.42261
S_numerator[54,2]	0.37511	0.02497	0.32685	0.42261
S_numerator[55,2]	0.37511	0.02497	0.32685	0.42261
S_numerator[56,2]	0.37511	0.02497	0.32685	0.42261
S_numerator[57,2]	0.19169	0.03232	0.13213	0.25850
S_numerator[58,2]	0.19169	0.03232	0.13213	0.25850
S_numerator[59,2]	0.19169	0.03232	0.13213	0.25850
S_numerator[60,2]	0.19169	0.03232	0.13213	0.25850
S_numerator[61,2]	0.19169	0.03232	0.13213	0.25850
S_numerator[62,2]	0.19169	0.03232	0.13213	0.25850
S_numerator[63,2]	0.19169	0.03232	0.13213	0.25850
S_numerator[64,2]	0.19169	0.03232	0.13213	0.25850
S_numerator[65,2]	0.19169	0.03232	0.13213	0.25850
S_numerator[66,2]	0.19169	0.03232	0.13213	0.25850
S_numerator[67,2]	0.19169	0.03232	0.13213	0.25850
S_numerator[68,2]	0.19169	0.03232	0.13213	0.25850
S_numerator[69,2]	0.19169	0.03232	0.13213	0.25850
S_numerator[26,3]	0.50605	0.05592	0.39572	0.61251
S_numerator[27,3]	0.34020	0.04732	0.24912	0.43280
S_numerator[28,3]	0.15029	0.02328	0.10831	0.19764
S_numerator[32,3]	0.37262	0.05542	0.26533	0.48055
S_numerator[36,3]	0.39866	0.06796	0.26303	0.51607
S_numerator[42,3]	0.34966	0.04755	0.25807	0.44408
S_numerator[43,3]	0.16760	0.03437	0.10430	0.23913
S_numerator[44,3]	0.31193	0.02382	0.26598	0.35815
S_numerator[45,3]	0.31193	0.02382	0.26598	0.35815
S_numerator[46,3]	0.31193	0.02382	0.26598	0.35815
S_numerator[47,3]	0.31193	0.02382	0.26598	0.35815
S_numerator[48,3]	0.31193	0.02382	0.26598	0.35815
S_numerator[49,3]	0.31193	0.02382	0.26598	0.35815
S_numerator[50,3]	0.31193	0.02382	0.26598	0.35815
S_numerator[51,3]	0.31193	0.02382	0.26598	0.35815
S_numerator[52,3]	0.31193	0.02382	0.26598	0.35815
S_numerator[53,3]	0.31193	0.02382	0.26598	0.35815
S_numerator[54,3]	0.31193	0.02382	0.26598	0.35815
S_numerator[55,3]	0.31193	0.02382	0.26598	0.35815
S_numerator[56,3]	0.31193	0.02382	0.26598	0.35815
S_numerator[57,3]	0.14087	0.02779	0.09098	0.19816
S_numerator[58,3]	0.14087	0.02779	0.09098	0.19816
S_numerator[59,3]	0.14087	0.02779	0.09098	0.19816
S_numerator[60,3]	0.14087	0.02779	0.09098	0.19816
S_numerator[61,3]	0.14087	0.02779	0.09098	0.19816
S_numerator[62,3]	0.14087	0.02779	0.09098	0.19816
S_numerator[63,3]	0.14087	0.02779	0.09098	0.19816
S_numerator[64,3]	0.14087	0.02779	0.09098	0.19816
S_numerator[65,3]	0.14087	0.02779	0.09098	0.19816
S_numerator[66,3]	0.14087	0.02779	0.09098	0.19816
S_numerator[67,3]	0.14087	0.02779	0.09098	0.19816
S_numerator[68,3]	0.14087	0.02779	0.09098	0.19816
S_numerator[69,3]	0.14087	0.02779	0.09098	0.19816
S_numerator[26,4]	0.45665	0.05654	0.34656	0.56521
S_numerator[27,4]	0.29057	0.04538	0.20373	0.38124
S_numerator[28,4]	0.11454	0.01980	0.07917	0.15486
S_numerator[32,4]	0.32444	0.05462	0.22040	0.43235
S_numerator[33,4]	0.32653	0.05502	0.22214	0.43555
S_numerator[34,4]	0.67909	0.09650	0.46064	0.84154

© 2018 The Authors. Journal compilation © 2018 British Ecological Society, Ecology&Evolution

S_numerator[35,4]	0.59831	0.12473	0.33173	0.81373
S_numerator[36,4]	0.32793	0.05702	0.21472	0.43007
S_numerator[41,4]	0.76063	0.06343	0.62034	0.86618
S_numerator[42,4]	0.30214	0.04640	0.21436	0.39501
S_numerator[43,4]	0.13090	0.03031	0.07702	0.19472
S_numerator[44,4]	0.26499	0.02247	0.22204	0.30833
S_numerator[45,4]	0.26499	0.02247	0.22204	0.30833
S_numerator[46,4]	0.26499	0.02247	0.22204	0.30833
S_numerator[47,4]	0.26499	0.02247	0.22204	0.30833
S_numerator[48,4]	0.26499	0.02247	0.22204	0.30833
S_numerator[49,4]	0.26499	0.02247	0.22204	0.30833
S_numerator[50,4]	0.26499	0.02247	0.22204	0.30833
S_numerator[51,4]	0.26499	0.02247	0.22204	0.30833
S_numerator[52,4]	0.26499	0.02247	0.22204	0.30833
S_numerator[53,4]	0.26499	0.02247	0.22204	0.30833
S_numerator[54,4]	0.26499	0.02247	0.22204	0.30833
S_numerator[55,4]	0.26499	0.02247	0.22204	0.30833
S_numerator[56,4]	0.26499	0.02247	0.22204	0.30833
S_numerator[57,4]	0.10735	0.02388	0.06540	0.15710
S_numerator[58,4]	0.10735	0.02388	0.06540	0.15710
S_numerator[59,4]	0.10735	0.02388	0.06540	0.15710
S_numerator[60,4]	0.10735	0.02388	0.06540	0.15710
S_numerator[61,4]	0.10735	0.02388	0.06540	0.15710
S_numerator[62,4]	0.10735	0.02388	0.06540	0.15710
S_numerator[63,4]	0.10735	0.02388	0.06540	0.15710
S_numerator[64,4]	0.10735	0.02388	0.06540	0.15710
S_numerator[65,4]	0.10735	0.02388	0.06540	0.15710
S_numerator[66,4]	0.10735	0.02388	0.06540	0.15710
S_numerator[67,4]	0.10735	0.02388	0.06540	0.15710
S_numerator[68,4]	0.10735	0.02388	0.06540	0.15710
S_numerator[69,4]	0.10735	0.02388	0.06540	0.15710
S_numerator[26,5]	0.41398	0.05650	0.30525	0.52291
S_numerator[27,5]	0.25003	0.04311	0.16886	0.33757
S_numerator[28,5]	0.08843	0.01681	0.05888	0.12318
S_numerator[29,5]	0.42910	0.06107	0.31186	0.54877
S_numerator[30,5]	0.25916	0.04586	0.17431	0.35061
S_numerator[31,5]	0.09166	0.01781	0.06023	0.12882
S_numerator[33,5]	0.28630	0.05357	0.18567	0.39415
S_numerator[34,5]	0.64946	0.10231	0.42152	0.82431
S_numerator[35,5]	0.56471	0.12997	0.29246	0.79415
S_numerator[36,5]	0.27130	0.04790	0.17646	0.35984
S_numerator[37,5]	0.31885	0.06985	0.18405	0.44234
S_numerator[38,5]	0.44244	0.10064	0.25363	0.62838
S_numerator[39,5]	0.44244	0.10064	0.25363	0.62838
S_numerator[41,5]	0.73646	0.06821	0.58626	0.85151
S_numerator[42,5]	0.26302	0.04477	0.17870	0.35427
S_numerator[43,5]	0.10354	0.02659	0.05701	0.16116
S_numerator[44,5]	0.22693	0.02104	0.18671	0.26759
S_numerator[45,5]	0.22693	0.02104	0.18671	0.26759
S_numerator[46,5]	0.22693	0.02104	0.18671	0.26759
S_numerator[47,5]	0.22693	0.02104	0.18671	0.26759
S_numerator[48,5]	0.22693	0.02104	0.18671	0.26759
S_numerator[49,5]	0.22693	0.02104	0.18671	0.26759
S_numerator[50,5]	0.22693	0.02104	0.18671	0.26759
S_numerator[51,5]	0.22693	0.02104	0.18671	0.26759
S_numerator[52,5]	0.22693	0.02104	0.18671	0.26759
S_numerator[53,5]	0.22693	0.02104	0.18671	0.26759
S_numerator[54,5]	0.22693	0.02104	0.18671	0.26759
S_numerator[55,5]	0.22693	0.02104	0.18671	0.26759
S_numerator[56,5]	0.22693	0.02104	0.18671	0.26759
S_numerator[57,5]	0.08295	0.02045	0.04778	0.12670
S_numerator[58,5]	0.08295	0.02045	0.04778	0.12670
S_numerator[59,5]	0.08295	0.02045	0.04778	0.12670
S_numerator[60,5]	0.08295	0.02045	0.04778	0.12670
S_numerator[61,5]	0.08295	0.02045	0.04778	0.12670
S_numerator[62,5]	0.08295	0.02045	0.04778	0.12670
S_numerator[63,5]	0.08295	0.02045	0.04778	0.12670
S_numerator[64,5]	0.08295	0.02045	0.04778	0.12670
S_numerator[65,5]	0.08295	0.02045	0.04778	0.12670
S_numerator[66,5]	0.08295	0.02045	0.04778	0.12670
S_numerator[67,5]	0.08295	0.02045	0.04778	0.12670
S_numerator[68,5]	0.08295	0.02045	0.04778	0.12670

© 2018 The Authors. Journal compilation © 2018 British Ecological Society, Ecology&Evolution

S_numerator[69,5]	0.08295	0.02045	0.04778	0.12670	S_numerator[66,6]	0.06420	0.01737	0.03529	0.10186
S_numerator[29,6]	0.38921	0.06009	0.27496	0.50746	S_numerator[67,6]	0.06420	0.01737	0.03529	0.10186
S_numerator[30,6]	0.22319	0.04302	0.14391	0.31075	S_numerator[68,6]	0.06420	0.01737	0.03529	0.10186
S_numerator[31,6]	0.07087	0.01496	0.04489	0.10267	S_numerator[69,6]	0.06420	0.01737	0.03529	0.10186
S_numerator[33,6]	0.25126	0.05167	0.15562	0.35665	S_numerator[29,7]	0.35228	0.05883	0.24127	0.46928
S_numerator[34,6]	0.62141	0.10733	0.38589	0.80758	S_numerator[30,7]	0.19156	0.04006	0.11861	0.27498
S_numerator[35,6]	0.53342	0.13410	0.25716	0.77433	S_numerator[31,7]	0.05445	0.01247	0.03302	0.08120
S_numerator[36,6]	0.22458	0.04034	0.14531	0.30183	S_numerator[33,7]	0.21983	0.04940	0.13003	0.32191
S_numerator[37,6]	0.26389	0.05834	0.15213	0.37018	S_numerator[34,7]	0.59404	0.11177	0.35116	0.79091
S_numerator[38,6]	0.38012	0.08771	0.21663	0.54557	S_numerator[35,7]	0.50337	0.13735	0.22464	0.75512
S_numerator[39,6]	0.38012	0.08771	0.21663	0.54557	S_numerator[36,7]	0.18537	0.03407	0.11899	0.25233
S_numerator[41,6]	0.71322	0.07256	0.55321	0.83637	S_numerator[37,7]	0.21780	0.04881	0.12520	0.31033
S_numerator[42,6]	0.22916	0.04281	0.14976	0.31708	S_numerator[38,7]	0.32598	0.07672	0.18391	0.47321
S_numerator[43,6]	0.08204	0.02314	0.04236	0.13302	S_numerator[39,7]	0.32598	0.07672	0.18391	0.47321
S_numerator[44,6]	0.19448	0.01958	0.15701	0.23231	S_numerator[41,7]	0.69020	0.07663	0.52157	0.82108
S_numerator[45,6]	0.19448	0.01958	0.15701	0.23231	S_numerator[42,7]	0.19898	0.04056	0.12479	0.28309
S_numerator[46,6]	0.19448	0.01958	0.15701	0.23231	S_numerator[43,7]	0.06463	0.01992	0.03129	0.10901
S_numerator[47,6]	0.19448	0.01958	0.15701	0.23231	S_numerator[44,7]	0.16599	0.01809	0.13170	0.20166
S_numerator[48,6]	0.19448	0.01958	0.15701	0.23231	S_numerator[45,7]	0.16599	0.01809	0.13170	0.20166
S_numerator[49,6]	0.19448	0.01958	0.15701	0.23231	S_numerator[46,7]	0.16599	0.01809	0.13170	0.20166
S_numerator[50,6]	0.19448	0.01958	0.15701	0.23231	S_numerator[47,7]	0.16599	0.01809	0.13170	0.20166
S_numerator[51,6]	0.19448	0.01958	0.15701	0.23231	S_numerator[48,7]	0.16599	0.01809	0.13170	0.20166
S_numerator[52,6]	0.19448	0.01958	0.15701	0.23231	S_numerator[49,7]	0.16599	0.01809	0.13170	0.20166
S_numerator[53,6]	0.19448	0.01958	0.15701	0.23231	S_numerator[50,7]	0.16599	0.01809	0.13170	0.20166
S_numerator[54,6]	0.19448	0.01958	0.15701	0.23231	S_numerator[51,7]	0.16599	0.01809	0.13170	0.20166
S_numerator[55,6]	0.19448	0.01958	0.15701	0.23231	S_numerator[52,7]	0.16599	0.01809	0.13170	0.20166
S_numerator[56,6]	0.19448	0.01958	0.15701	0.23231	S_numerator[53,7]	0.16599	0.01809	0.13170	0.20166
S_numerator[57,6]	0.06420	0.01737	0.03529	0.10186	S_numerator[54,7]	0.16599	0.01809	0.13170	0.20166
S_numerator[58,6]	0.06420	0.01737	0.03529	0.10186	S_numerator[55,7]	0.16599	0.01809	0.13170	0.20166
S_numerator[59,6]	0.06420	0.01737	0.03529	0.10186	S_numerator[56,7]	0.16599	0.01809	0.13170	0.20166
S_numerator[60,6]	0.06420	0.01737	0.03529	0.10186	S_numerator[57,7]	0.04938	0.01460	0.02561	0.08188
S_numerator[61,6]	0.06420	0.01737	0.03529	0.10186	S_numerator[58,7]	0.04938	0.01460	0.02561	0.08188
S_numerator[62,6]	0.06420	0.01737	0.03529	0.10186	S_numerator[59,7]	0.04938	0.01460	0.02561	0.08188
S_numerator[63,6]	0.06420	0.01737	0.03529	0.10186	S_numerator[60,7]	0.04938	0.01460	0.02561	0.08188
S_numerator[64,6]	0.06420	0.01737	0.03529	0.10186	S_numerator[61,7]	0.04938	0.01460	0.02561	0.08188
S_numerator[65,6]	0.06420	0.01737	0.03529	0.10186	S_numerator[62,7]	0.04938	0.01460	0.02561	0.08188

© 2018 The Authors. Journal compilation © 2018 British Ecological Society, Ecology&Evolution

S_numerator[63,7]	0.04938	0.01460	0.02561	0.08188
S_numerator[64,7]	0.04938	0.01460	0.02561	0.08188
S_numerator[65,7]	0.04938	0.01460	0.02561	0.08188
S_numerator[66,7]	0.04938	0.01460	0.02561	0.08188
S_numerator[67,7]	0.04938	0.01460	0.02561	0.08188
S_numerator[68,7]	0.04938	0.01460	0.02561	0.08188
S_numerator[69,7]	0.04938	0.01460	0.02561	0.08188
S_numerator[29,8]	0.31757	0.05731	0.20985	0.43150
S_numerator[30,8]	0.16335	0.03701	0.09675	0.24217
S_numerator[31,8]	0.04134	0.01026	0.02406	0.06365
S_numerator[33,8]	0.19120	0.04681	0.10774	0.28908
S_numerator[34,8]	0.56682	0.11571	0.31808	0.77335
S_numerator[35,8]	0.47400	0.13985	0.19511	0.73487
S_numerator[36,8]	0.15220	0.02886	0.09689	0.20951
S_numerator[37,8]	0.17881	0.04090	0.10135	0.25788
S_numerator[38,8]	0.27858	0.06737	0.15505	0.41044
S_numerator[39,8]	0.27858	0.06737	0.15505	0.41044
S_numerator[41,8]	0.66696	0.08051	0.48953	0.80538
S_numerator[42,8]	0.17167	0.03807	0.10349	0.25171
S_numerator[43,8]	0.05038	0.01692	0.02276	0.08857
S_numerator[44,8]	0.14061	0.01657	0.10975	0.17368
S_numerator[45,8]	0.14061	0.01657	0.10975	0.17368
S_numerator[46,8]	0.14061	0.01657	0.10975	0.17368
S_numerator[47,8]	0.14061	0.01657	0.10975	0.17368
S_numerator[48,8]	0.14061	0.01657	0.10975	0.17368
S_numerator[49,8]	0.14061	0.01657	0.10975	0.17368
S_numerator[50,8]	0.14061	0.01657	0.10975	0.17368
S_numerator[51,8]	0.14061	0.01657	0.10975	0.17368
S_numerator[52,8]	0.14061	0.01657	0.10975	0.17368
S_numerator[53,8]	0.14061	0.01657	0.10975	0.17368
S_numerator[54,8]	0.14061	0.01657	0.10975	0.17368
S_numerator[55,8]	0.14061	0.01657	0.10975	0.17368
S_numerator[56,8]	0.14061	0.01657	0.10975	0.17368
S_numerator[57,8]	0.03753	0.01209	0.01826	0.06464
S_numerator[58,8]	0.03753	0.01209	0.01826	0.06464
S_numerator[59,8]	0.03753	0.01209	0.01826	0.06464
S_numerator[60,8]	0.03753	0.01209	0.01826	0.06464
S_numerator[61,8]	0.03753	0.01209	0.01826	0.06464
S_numerator[62,8]	0.03753	0.01209	0.01826	0.06464
S_numerator[63,8]	0.03753	0.01209	0.01826	0.06464
S_numerator[64,8]	0.03753	0.01209	0.01826	0.06464
S_numerator[65,8]	0.03753	0.01209	0.01826	0.06464
S_numerator[66,8]	0.03753	0.01209	0.01826	0.06464
S_numerator[67,8]	0.03753	0.01209	0.01826	0.06464
S_numerator[68,8]	0.03753	0.01209	0.01826	0.06464
S_numerator[69,8]	0.03753	0.01209	0.01826	0.06464
S_numerator[29,9]	0.28470	0.05551	0.18143	0.39533
S_numerator[30,9]	0.13806	0.03387	0.07819	0.21119
S_numerator[31,9]	0.03089	0.00831	0.01715	0.04935
S_numerator[33,9]	0.16497	0.04391	0.08837	0.25794
S_numerator[34,9]	0.53947	0.11920	0.28636	0.75491
S_numerator[35,9]	0.44497	0.14162	0.16907	0.71461
S_numerator[36,9]	0.12406	0.02449	0.07771	0.17385
S_numerator[37,9]	0.14576	0.03431	0.08127	0.21322
S_numerator[38,9]	0.23694	0.05935	0.12880	0.35640
S_numerator[39,9]	0.23694	0.05935	0.12880	0.35640
S_numerator[41,9]	0.64324	0.08422	0.45950	0.78906
S_numerator[42,9]	0.14680	0.03536	0.08457	0.22211
S_numerator[43,9]	0.03869	0.01415	0.01613	0.07119
S_numerator[44,9]	0.11790	0.01503	0.09031	0.14825
S_numerator[45,9]	0.11790	0.01503	0.09031	0.14825
S_numerator[46,9]	0.11790	0.01503	0.09031	0.14825
S_numerator[47,9]	0.11790	0.01503	0.09031	0.14825
S_numerator[48,9]	0.11790	0.01503	0.09031	0.14825
S_numerator[49,9]	0.11790	0.01503	0.09031	0.14825
S_numerator[50,9]	0.11790	0.01503	0.09031	0.14825
S_numerator[51,9]	0.11790	0.01503	0.09031	0.14825
S_numerator[52,9]	0.11790	0.01503	0.09031	0.14825
S_numerator[53,9]	0.11790	0.01503	0.09031	0.14825
S_numerator[54,9]	0.11790	0.01503	0.09031	0.14825
S_numerator[55,9]	0.11790	0.01503	0.09031	0.14825
S_numerator[56,9]	0.11790	0.01503	0.09031	0.14825

© 2018 The Authors. Journal compilation © 2018 British Ecological Society, Ecology&Evolution

S_numerator[57,9]	0.02806	0.00984	0.01273	0.05025
S_numerator[58,9]	0.02806	0.00984	0.01273	0.05025
S_numerator[59,9]	0.02806	0.00984	0.01273	0.05025
S_numerator[60,9]	0.02806	0.00984	0.01273	0.05025
S_numerator[61,9]	0.02806	0.00984	0.01273	0.05025
S_numerator[62,9]	0.02806	0.00984	0.01273	0.05025
S_numerator[63,9]	0.02806	0.00984	0.01273	0.05025
S_numerator[64,9]	0.02806	0.00984	0.01273	0.05025
S_numerator[65,9]	0.02806	0.00984	0.01273	0.05025
S_numerator[66,9]	0.02806	0.00984	0.01273	0.05025
S_numerator[67,9]	0.02806	0.00984	0.01273	0.05025
S_numerator[68,9]	0.02806	0.00984	0.01273	0.05025
S_numerator[69,9]	0.02806	0.00984	0.01273	0.05025
S_numerator[29,10]	0.25353	0.05342	0.15521	0.36123
S_numerator[30,10]	0.11543	0.03067	0.06243	0.18159
S_numerator[31,10]	0.02264	0.00661	0.01195	0.03765
S_numerator[33,10]	0.14093	0.04075	0.07143	0.22836
S_numerator[34,10]	0.51184	0.12222	0.25599	0.73718
S_numerator[35,10]	0.41615	0.14266	0.14449	0.69389
S_numerator[36,10]	0.10024	0.02078	0.06139	0.14336
S_numerator[37,10]	0.11778	0.02877	0.06399	0.17565
S_numerator[38,10]	0.20035	0.05242	0.10636	0.30798
S_numerator[39,10]	0.20035	0.05242	0.10636	0.30798
S_numerator[40,10]	0.35765	0.11120	0.16910	0.59808
S_numerator[41,10]	0.61886	0.08776	0.42979	0.77224
S_numerator[42,10]	0.12420	0.03247	0.06794	0.19440
S_numerator[43,10]	0.02919	0.01162	0.01118	0.05648
S_numerator[44,10]	0.09763	0.01348	0.07320	0.12527
S_numerator[45,10]	0.09763	0.01348	0.07320	0.12527
S_numerator[46,10]	0.09763	0.01348	0.07320	0.12527
S_numerator[47,10]	0.09763	0.01348	0.07320	0.12527
S_numerator[48,10]	0.09763	0.01348	0.07320	0.12527
S_numerator[49,10]	0.09763	0.01348	0.07320	0.12527
S_numerator[50,10]	0.09763	0.01348	0.07320	0.12527
S_numerator[51,10]	0.09763	0.01348	0.07320	0.12527
S_numerator[52,10]	0.09763	0.01348	0.07320	0.12527
S_numerator[53,10]	0.09763	0.01348	0.07320	0.12527
S_numerator[54,10]	0.09763	0.01348	0.07320	0.12527
S_numerator[55,10]	0.09763	0.01348	0.07320	0.12527
S_numerator[56,10]	0.09763	0.01348	0.07320	0.12527
S_numerator[57,10]	0.02057	0.00786	0.00870	0.03857
S_numerator[58,10]	0.02057	0.00786	0.00870	0.03857
S_numerator[59,10]	0.02057	0.00786	0.00870	0.03857
S_numerator[60,10]	0.02057	0.00786	0.00870	0.03857
S_numerator[61,10]	0.02057	0.00786	0.00870	0.03857
S_numerator[62,10]	0.02057	0.00786	0.00870	0.03857
S_numerator[63,10]	0.02057	0.00786	0.00870	0.03857
S_numerator[64,10]	0.02057	0.00786	0.00870	0.03857
S_numerator[65,10]	0.02057	0.00786	0.00870	0.03857
S_numerator[66,10]	0.02057	0.00786	0.00870	0.03857
S_numerator[67,10]	0.02057	0.00786	0.00870	0.03857
S_numerator[68,10]	0.02057	0.00786	0.00870	0.03857
S_numerator[69,10]	0.02057	0.00786	0.00870	0.03857
S_numerator[33,11]	0.11904	0.03738	0.05648	0.20033
S_numerator[34,11]	0.48388	0.12473	0.22776	0.71833
S_numerator[35,11]	0.38753	0.14295	0.12182	0.67158
S_numerator[36,11]	0.08018	0.01762	0.04789	0.11724
S_numerator[37,11]	0.09423	0.02409	0.04974	0.14363
S_numerator[38,11]	0.16829	0.04634	0.08638	0.26459
S_numerator[39,11]	0.16829	0.04634	0.08638	0.26459
S_numerator[40,11]	0.31463	0.10255	0.14217	0.53598
S_numerator[41,11]	0.59377	0.09110	0.39862	0.75438
S_numerator[42,11]	0.10378	0.02943	0.05385	0.16834
S_numerator[43,11]	0.02157	0.00936	0.00763	0.04397
S_numerator[44,11]	0.07969	0.01193	0.05822	0.10425
S_numerator[45,11]	0.07969	0.01193	0.05822	0.10425
S_numerator[46,11]	0.07969	0.01193	0.05822	0.10425
S_numerator[47,11]	0.07969	0.01193	0.05822	0.10425
S_numerator[48,11]	0.07969	0.01193	0.05822	0.10425
S_numerator[49,11]	0.07969	0.01193	0.05822	0.10425
S_numerator[50,11]	0.07969	0.01193	0.05822	0.10425
S_numerator[51,11]	0.07969	0.01193	0.05822	0.10425

© 2018 The Authors. Journal compilation © 2018 British Ecological Society, Ecology&Evolution

S_numerator[52,11]	0.07969	0.01193	0.05822	0.10425	S_numerator[51,12]	0.06402	0.01040	0.04554	0.08570
S_numerator[53,11]	0.07969	0.01193	0.05822	0.10425	S_numerator[52,12]	0.06402	0.01040	0.04554	0.08570
S_numerator[54,11]	0.07969	0.01193	0.05822	0.10425	S_numerator[53,12]	0.06402	0.01040	0.04554	0.08570
S_numerator[55,11]	0.07969	0.01193	0.05822	0.10425	S_numerator[54,12]	0.06402	0.01040	0.04554	0.08570
S_numerator[56,11]	0.07969	0.01193	0.05822	0.10425	S_numerator[55,12]	0.06402	0.01040	0.04554	0.08570
S_numerator[57,11]	0.01473	0.00613	0.00577	0.02909	S_numerator[56,12]	0.06402	0.01040	0.04554	0.08570
S_numerator[58,11]	0.01473	0.00613	0.00577	0.02909	S_numerator[57,12]	0.01029	0.00467	0.00367	0.02139
S_numerator[59,11]	0.01473	0.00613	0.00577	0.02909	S_numerator[58,12]	0.01029	0.00467	0.00367	0.02139
S_numerator[60,11]	0.01473	0.00613	0.00577	0.02909	S_numerator[59,12]	0.01029	0.00467	0.00367	0.02139
S_numerator[61,11]	0.01473	0.00613	0.00577	0.02909	S_numerator[60,12]	0.01029	0.00467	0.00367	0.02139
S_numerator[62,11]	0.01473	0.00613	0.00577	0.02909	S_numerator[61,12]	0.01029	0.00467	0.00367	0.02139
S_numerator[63,11]	0.01473	0.00613	0.00577	0.02909	S_numerator[62,12]	0.01029	0.00467	0.00367	0.02139
S_numerator[64,11]	0.01473	0.00613	0.00577	0.02909	S_numerator[63,12]	0.01029	0.00467	0.00367	0.02139
S_numerator[65,11]	0.01473	0.00613	0.00577	0.02909	S_numerator[64,12]	0.01029	0.00467	0.00367	0.02139
S_numerator[66,11]	0.01473	0.00613	0.00577	0.02909	S_numerator[65,12]	0.01029	0.00467	0.00367	0.02139
S_numerator[67,11]	0.01473	0.00613	0.00577	0.02909	S_numerator[66,12]	0.01029	0.00467	0.00367	0.02139
S_numerator[68,11]	0.01473	0.00613	0.00577	0.02909	S_numerator[67,12]	0.01029	0.00467	0.00367	0.02139
S_numerator[69,11]	0.01473	0.00613	0.00577	0.02909	S_numerator[68,12]	0.01029	0.00467	0.00367	0.02139
S_numerator[33,12]	0.09930	0.03384	0.04390	0.17406	S_numerator[69,12]	0.01029	0.00467	0.00367	0.02139
S_numerator[34,12]	0.45564	0.12668	0.20123	0.69734	S_numerator[33,13]	0.08170	0.03023	0.03344	0.14975
S_numerator[35,12]	0.35918	0.14245	0.10123	0.64842	S_numerator[34,13]	0.42721	0.12804	0.17507	0.67532
S_numerator[36,12]	0.06341	0.01488	0.03657	0.09531	S_numerator[35,13]	0.33121	0.14117	0.08274	0.62444
S_numerator[37,12]	0.07455	0.02011	0.03803	0.11611	S_numerator[36,13]	0.04954	0.01251	0.02728	0.07661
S_numerator[38,12]	0.14030	0.04095	0.06898	0.22623	S_numerator[37,13]	0.05827	0.01671	0.02853	0.09360
S_numerator[39,12]	0.14030	0.04095	0.06898	0.22623	S_numerator[38,13]	0.11603	0.03612	0.05442	0.19286
S_numerator[40,12]	0.27518	0.09475	0.11809	0.47885	S_numerator[39,13]	0.11603	0.03612	0.05442	0.19286
S_numerator[41,12]	0.56794	0.09421	0.36746	0.73547	S_numerator[40,13]	0.23915	0.08754	0.09589	0.42827
S_numerator[42,12]	0.08553	0.02632	0.04169	0.14409	S_numerator[41,13]	0.54143	0.09704	0.33682	0.71566
S_numerator[43,12]	0.01558	0.00737	0.00503	0.03346	S_numerator[42,13]	0.06945	0.02318	0.03177	0.12170
S_numerator[44,12]	0.06402	0.01040	0.04554	0.08570	S_numerator[43,13]	0.01097	0.00566	0.00316	0.02498
S_numerator[45,12]	0.06402	0.01040	0.04554	0.08570	S_numerator[44,13]	0.05053	0.00891	0.03485	0.06929
S_numerator[46,12]	0.06402	0.01040	0.04554	0.08570	S_numerator[45,13]	0.05053	0.00891	0.03485	0.06929
S_numerator[47,12]	0.06402	0.01040	0.04554	0.08570	S_numerator[46,13]	0.05053	0.00891	0.03485	0.06929
S_numerator[48,12]	0.06402	0.01040	0.04554	0.08570	S_numerator[47,13]	0.05053	0.00891	0.03485	0.06929
S_numerator[49,12]	0.06402	0.01040	0.04554	0.08570	S_numerator[48,13]	0.05053	0.00891	0.03485	0.06929
S_numerator[50,12]	0.06402	0.01040	0.04554	0.08570	S_numerator[49,13]	0.05053	0.00891	0.03485	0.06929

© 2018 The Authors. Journal compilation © 2018 British Ecological Society, Ecology&Evolution

S_numerator[50,13]	0.05053	0.00891	0.03485	0.06929	S_numerator[50,14]	0.03914	0.00750	0.02608	0.05515
S_numerator[51,13]	0.05053	0.00891	0.03485	0.06929	S_numerator[51,14]	0.03914	0.00750	0.02608	0.05515
S_numerator[52,13]	0.05053	0.00891	0.03485	0.06929	S_numerator[52,14]	0.03914	0.00750	0.02608	0.05515
S_numerator[53,13]	0.05053	0.00891	0.03485	0.06929	S_numerator[53,14]	0.03914	0.00750	0.02608	0.05515
S_numerator[54,13]	0.05053	0.00891	0.03485	0.06929	S_numerator[54,14]	0.03914	0.00750	0.02608	0.05515
S_numerator[55,13]	0.05053	0.00891	0.03485	0.06929	S_numerator[55,14]	0.03914	0.00750	0.02608	0.05515
S_numerator[56,13]	0.05053	0.00891	0.03485	0.06929	S_numerator[56,14]	0.03914	0.00750	0.02608	0.05515
S_numerator[57,13]	0.00699	0.00346	0.00226	0.01532	S_numerator[57,14]	0.00461	0.00249	0.00134	0.01070
S_numerator[58,13]	0.00699	0.00346	0.00226	0.01532	S_numerator[58,14]	0.00461	0.00249	0.00134	0.01070
S_numerator[59,13]	0.00699	0.00346	0.00226	0.01532	S_numerator[59,14]	0.00461	0.00249	0.00134	0.01070
S_numerator[60,13]	0.00699	0.00346	0.00226	0.01532	S_numerator[60,14]	0.00461	0.00249	0.00134	0.01070
S_numerator[61,13]	0.00699	0.00346	0.00226	0.01532	S_numerator[61,14]	0.00461	0.00249	0.00134	0.01070
S_numerator[62,13]	0.00699	0.00346	0.00226	0.01532	S_numerator[62,14]	0.00461	0.00249	0.00134	0.01070
S_numerator[63,13]	0.00699	0.00346	0.00226	0.01532	S_numerator[63,14]	0.00461	0.00249	0.00134	0.01070
S_numerator[64,13]	0.00699	0.00346	0.00226	0.01532	S_numerator[64,14]	0.00461	0.00249	0.00134	0.01070
S_numerator[65,13]	0.00699	0.00346	0.00226	0.01532	S_numerator[65,14]	0.00461	0.00249	0.00134	0.01070
S_numerator[66,13]	0.00699	0.00346	0.00226	0.01532	S_numerator[66,14]	0.00461	0.00249	0.00134	0.01070
S_numerator[67,13]	0.00699	0.00346	0.00226	0.01532	S_numerator[67,14]	0.00461	0.00249	0.00134	0.01070
S_numerator[68,13]	0.00699	0.00346	0.00226	0.01532	S_numerator[68,14]	0.00461	0.00249	0.00134	0.01070
S_numerator[69,13]	0.00699	0.00346	0.00226	0.01532	S_numerator[69,14]	0.00461	0.00249	0.00134	0.01070
S_numerator[33,14]	0.06624	0.02661	0.02497	0.12707	S_numerator[33,15]	0.05288	0.02306	0.01820	0.10633
S_numerator[34,14]	0.39874	0.12874	0.15048	0.65241	S_numerator[34,15]	0.37039	0.12875	0.12790	0.62864
S_numerator[35,14]	0.30379	0.13908	0.06653	0.59892	S_numerator[35,15]	0.27709	0.13620	0.05259	0.57323
S_numerator[36,14]	0.03819	0.01043	0.01996	0.06099	S_numerator[36,15]	0.02904	0.00862	0.01429	0.04810
S_numerator[38,14]	0.09513	0.03175	0.04203	0.16371	S_numerator[38,15]	0.07730	0.02777	0.03180	0.13836
S_numerator[39,14]	0.09513	0.03175	0.04203	0.16371	S_numerator[39,15]	0.07730	0.02777	0.03180	0.13836
S_numerator[40,14]	0.20643	0.08072	0.07735	0.38446	S_numerator[40,15]	0.17691	0.07417	0.06091	0.34420
S_numerator[41,14]	0.51430	0.09955	0.30696	0.69492	S_numerator[41,15]	0.48668	0.10167	0.27786	0.67354
S_numerator[42,14]	0.05549	0.02010	0.02357	0.10138	S_numerator[42,15]	0.04358	0.01713	0.01707	0.08319
S_numerator[43,14]	0.00753	0.00424	0.00193	0.01820	S_numerator[43,15]	0.00502	0.00310	0.00113	0.01291
S_numerator[44,14]	0.03914	0.00750	0.02608	0.05515	S_numerator[44,15]	0.02971	0.00619	0.01906	0.04304
S_numerator[45,14]	0.03914	0.00750	0.02608	0.05515	S_numerator[45,15]	0.02971	0.00619	0.01906	0.04304
S_numerator[46,14]	0.03914	0.00750	0.02608	0.05515	S_numerator[46,15]	0.02971	0.00619	0.01906	0.04304
S_numerator[47,14]	0.03914	0.00750	0.02608	0.05515	S_numerator[47,15]	0.02971	0.00619	0.01906	0.04304
S_numerator[48,14]	0.03914	0.00750	0.02608	0.05515	S_numerator[48,15]	0.02971	0.00619	0.01906	0.04304
S_numerator[49,14]	0.03914	0.00750	0.02608	0.05515	S_numerator[49,15]	0.02971	0.00619	0.01906	0.04304

© 2018 The Authors. Journal compilation © 2018 British Ecological Society, Ecology&Evolution

S_numerator[50,15]	0.02971	0.00619	0.01906	0.04304
S_numerator[51,15]	0.02971	0.00619	0.01906	0.04304
S_numerator[52,15]	0.02971	0.00619	0.01906	0.04304
S_numerator[53,15]	0.02971	0.00619	0.01906	0.04304
S_numerator[54,15]	0.02971	0.00619	0.01906	0.04304
S_numerator[55,15]	0.02971	0.00619	0.01906	0.04304
S_numerator[56,15]	0.02971	0.00619	0.01906	0.04304
S_numerator[57,15]	0.00294	0.00174	0.00076	0.00729
S_numerator[58,15]	0.00294	0.00174	0.00076	0.00729
S_numerator[59,15]	0.00294	0.00174	0.00076	0.00729
S_numerator[60,15]	0.00294	0.00174	0.00076	0.00729
S_numerator[61,15]	0.00294	0.00174	0.00076	0.00729
S_numerator[62,15]	0.00294	0.00174	0.00076	0.00729
S_numerator[63,15]	0.00294	0.00174	0.00076	0.00729
S_numerator[64,15]	0.00294	0.00174	0.00076	0.00729
S_numerator[65,15]	0.00294	0.00174	0.00076	0.00729
S_numerator[66,15]	0.00294	0.00174	0.00076	0.00729
S_numerator[67,15]	0.00294	0.00174	0.00076	0.00729
S_numerator[68,15]	0.00294	0.00174	0.00076	0.00729
S_numerator[69,15]	0.00294	0.00174	0.00076	0.00729
S_numerator[33,16]	0.04154	0.01966	0.01292	0.08792
S_numerator[34,16]	0.34235	0.12804	0.10706	0.60413
S_numerator[35,16]	0.25130	0.13256	0.04103	0.54695
S_numerator[36,16]	0.02176	0.00705	0.00994	0.03755
S_numerator[38,16]	0.06222	0.02415	0.02364	0.11609
S_numerator[39,16]	0.06222	0.02415	0.02364	0.11609
S_numerator[40,16]	0.15048	0.06782	0.04734	0.30842
S_numerator[41,16]	0.45871	0.10335	0.24949	0.65171
S_numerator[42,16]	0.03362	0.01435	0.01206	0.06730
S_numerator[43,16]	0.00325	0.00220	0.00063	0.00892
S_numerator[44,16]	0.02207	0.00501	0.01360	0.03305
S_numerator[45,16]	0.02207	0.00501	0.01360	0.03305
S_numerator[46,16]	0.02207	0.00501	0.01360	0.03305
S_numerator[47,16]	0.02207	0.00501	0.01360	0.03305
S_numerator[48,16]	0.02207	0.00501	0.01360	0.03305
S_numerator[49,16]	0.02207	0.00501	0.01360	0.03305

S_numerator[50,16]	0.02207	0.00501	0.01360	0.03305
S_numerator[51,16]	0.02207	0.00501	0.01360	0.03305
S_numerator[52,16]	0.02207	0.00501	0.01360	0.03305
S_numerator[53,16]	0.02207	0.00501	0.01360	0.03305
S_numerator[54,16]	0.02207	0.00501	0.01360	0.03305
S_numerator[55,16]	0.02207	0.00501	0.01360	0.03305
S_numerator[56,16]	0.02207	0.00501	0.01360	0.03305
S_numerator[57,16]	0.00182	0.00117	0.00041	0.00480
S_numerator[58,16]	0.00182	0.00117	0.00041	0.00480
S_numerator[59,16]	0.00182	0.00117	0.00041	0.00480
S_numerator[60,16]	0.00182	0.00117	0.00041	0.00480
S_numerator[61,16]	0.00182	0.00117	0.00041	0.00480
S_numerator[62,16]	0.00182	0.00117	0.00041	0.00480
S_numerator[63,16]	0.00182	0.00117	0.00041	0.00480
S_numerator[64,16]	0.00182	0.00117	0.00041	0.00480
S_numerator[65,16]	0.00182	0.00117	0.00041	0.00480
S_numerator[66,16]	0.00182	0.00117	0.00041	0.00480
S_numerator[67,16]	0.00182	0.00117	0.00041	0.00480
S_numerator[68,16]	0.00182	0.00117	0.00041	0.00480
S_numerator[69,16]	0.00182	0.00117	0.00041	0.00480
S_numerator[33,17]	0.03208	0.01648	0.00893	0.07157
S_numerator[34,17]	0.31482	0.12661	0.08843	0.57911
S_numerator[35,17]	0.22658	0.12820	0.03142	0.51931
S_numerator[36,17]	0.01607	0.00569	0.00677	0.02895
S_numerator[38,17]	0.04960	0.02085	0.01725	0.09684
S_numerator[39,17]	0.04960	0.02085	0.01725	0.09684
S_numerator[40,17]	0.12701	0.06165	0.03636	0.27396
S_numerator[41,17]	0.43056	0.10454	0.22240	0.62868
S_numerator[44,17]	0.01604	0.00396	0.00942	0.02486
S_numerator[45,17]	0.01604	0.00396	0.00942	0.02486
S_numerator[46,17]	0.01604	0.00396	0.00942	0.02486
S_numerator[47,17]	0.01604	0.00396	0.00942	0.02486
S_numerator[48,17]	0.01604	0.00396	0.00942	0.02486
S_numerator[49,17]	0.01604	0.00396	0.00942	0.02486
S_numerator[50,17]	0.01604	0.00396	0.00942	0.02486
S_numerator[51,17]	0.01604	0.00396	0.00942	0.02486

© 2018 The Authors. Journal compilation © 2018 British Ecological Society, Ecology&Evolution

S_numerator[52,17]	0.01604	0.00396	0.00942	0.02486
S_numerator[53,17]	0.01604	0.00396	0.00942	0.02486
S_numerator[54,17]	0.01604	0.00396	0.00942	0.02486
S_numerator[55,17]	0.01604	0.00396	0.00942	0.02486
S_numerator[56,17]	0.01604	0.00396	0.00942	0.02486
S_numerator[57,17]	0.00109	0.00077	0.00021	0.00307
S_numerator[58,17]	0.00109	0.00077	0.00021	0.00307
S_numerator[59,17]	0.00109	0.00077	0.00021	0.00307
S_numerator[60,17]	0.00109	0.00077	0.00021	0.00307
S_numerator[61,17]	0.00109	0.00077	0.00021	0.00307
S_numerator[62,17]	0.00109	0.00077	0.00021	0.00307
S_numerator[63,17]	0.00109	0.00077	0.00021	0.00307
S_numerator[64,17]	0.00109	0.00077	0.00021	0.00307
S_numerator[65,17]	0.00109	0.00077	0.00021	0.00307
S_numerator[66,17]	0.00109	0.00077	0.00021	0.00307
S_numerator[67,17]	0.00109	0.00077	0.00021	0.00307
S_numerator[68,17]	0.00109	0.00077	0.00021	0.00307
S_numerator[69,17]	0.00109	0.00077	0.00021	0.00307
S_numerator[33,18]	0.02434	0.01358	0.00601	0.05756
S_numerator[34,18]	0.28800	0.12445	0.07235	0.55341
S_numerator[35,18]	0.20309	0.12320	0.02332	0.49115
S_numerator[36,18]	0.01168	0.00453	0.00449	0.02206
S_numerator[39,18]	0.03915	0.01786	0.01227	0.08017
S_numerator[40,18]	0.10635	0.05568	0.02735	0.24243
S_numerator[41,18]	0.40241	0.10521	0.19560	0.60435
S_numerator[44,18]	0.01138	0.00306	0.00639	0.01821
S_numerator[45,18]	0.01138	0.00306	0.00639	0.01821
S_numerator[46,18]	0.01138	0.00306	0.00639	0.01821
S_numerator[47,18]	0.01138	0.00306	0.00639	0.01821
S_numerator[48,18]	0.01138	0.00306	0.00639	0.01821
S_numerator[49,18]	0.01138	0.00306	0.00639	0.01821
S_numerator[50,18]	0.01138	0.00306	0.00639	0.01821
S_numerator[51,18]	0.01138	0.00306	0.00639	0.01821
S_numerator[52,18]	0.01138	0.00306	0.00639	0.01821
S_numerator[53,18]	0.01138	0.00306	0.00639	0.01821
S_numerator[54,18]	0.01138	0.00306	0.00639	0.01821

S_numerator[55,18]	0.01138	0.00306	0.00639	0.01821
S_numerator[56,18]	0.01138	0.00306	0.00639	0.01821
S_numerator[57,18]	0.00063	0.00049	0.00010	0.00188
S_numerator[58,18]	0.00063	0.00049	0.00010	0.00188
S_numerator[59,18]	0.00063	0.00049	0.00010	0.00188
S_numerator[60,18]	0.00063	0.00049	0.00010	0.00188
S_numerator[61,18]	0.00063	0.00049	0.00010	0.00188
S_numerator[62,18]	0.00063	0.00049	0.00010	0.00188
S_numerator[63,18]	0.00063	0.00049	0.00010	0.00188
S_numerator[64,18]	0.00063	0.00049	0.00010	0.00188
S_numerator[65,18]	0.00063	0.00049	0.00010	0.00188
S_numerator[66,18]	0.00063	0.00049	0.00010	0.00188
S_numerator[67,18]	0.00063	0.00049	0.00010	0.00188
S_numerator[68,18]	0.00063	0.00049	0.00010	0.00188
S_numerator[69,18]	0.00063	0.00049	0.00010	0.00188
S_numerator[33,19]	0.01814	0.01099	0.00394	0.04561
S_numerator[34,19]	0.26206	0.12159	0.05844	0.52716
S_numerator[35,19]	0.18096	0.11764	0.01706	0.46302
S_numerator[36,19]	0.00835	0.00356	0.00288	0.01663
S_numerator[39,19]	0.03059	0.01518	0.00855	0.06622
S_numerator[40,19]	0.08834	0.04992	0.02003	0.21264
S_numerator[41,19]	0.37444	0.10532	0.17050	0.57937
S_numerator[44,19]	0.00789	0.00231	0.00416	0.01314
S_numerator[45,19]	0.00789	0.00231	0.00416	0.01314
S_numerator[46,19]	0.00789	0.00231	0.00416	0.01314
S_numerator[47,19]	0.00789	0.00231	0.00416	0.01314
S_numerator[48,19]	0.00789	0.00231	0.00416	0.01314
S_numerator[49,19]	0.00789	0.00231	0.00416	0.01314
S_numerator[50,19]	0.00789	0.00231	0.00416	0.01314
S_numerator[51,19]	0.00789	0.00231	0.00416	0.01314
S_numerator[52,19]	0.00789	0.00231	0.00416	0.01314
S_numerator[53,19]	0.00789	0.00231	0.00416	0.01314
S_numerator[54,19]	0.00789	0.00231	0.00416	0.01314
S_numerator[55,19]	0.00789	0.00231	0.00416	0.01314
S_numerator[56,19]	0.00789	0.00231	0.00416	0.01314
S_numerator[57,19]	0.00035	0.00030	0.00005	0.00112

© 2018 The Authors. Journal compilation © 2018 British Ecological Society, Ecology&Evolution

S_numerator[58,19]	0.00035	0.00030	0.00005	0.00112
S_numerator[59,19]	0.00035	0.00030	0.00005	0.00112
S_numerator[60,19]	0.00035	0.00030	0.00005	0.00112
S_numerator[61,19]	0.00035	0.00030	0.00005	0.00112
S_numerator[62,19]	0.00035	0.00030	0.00005	0.00112
S_numerator[63,19]	0.00035	0.00030	0.00005	0.00112
S_numerator[64,19]	0.00035	0.00030	0.00005	0.00112
S_numerator[65,19]	0.00035	0.00030	0.00005	0.00112
S_numerator[66,19]	0.00035	0.00030	0.00005	0.00112
S_numerator[67,19]	0.00035	0.00030	0.00005	0.00112
S_numerator[68,19]	0.00035	0.00030	0.00005	0.00112
S_numerator[69,19]	0.00035	0.00030	0.00005	0.00112
S_numerator[33,20]	0.01328	0.00873	0.00250	0.03560
S_numerator[34,20]	0.23718	0.11807	0.04652	0.50047
S_numerator[35,20]	0.16028	0.11162	0.01227	0.43527
S_numerator[36,20]	0.00588	0.00275	0.00180	0.01233
S_numerator[39,20]	0.02366	0.01278	0.00585	0.05414
S_numerator[40,20]	0.07278	0.04442	0.01445	0.18663
S_numerator[41,20]	0.34683	0.10486	0.14741	0.55409
S_numerator[44,20]	0.00533	0.00170	0.00265	0.00925
S_numerator[45,20]	0.00533	0.00170	0.00265	0.00925
S_numerator[46,20]	0.00533	0.00170	0.00265	0.00925
S_numerator[47,20]	0.00533	0.00170	0.00265	0.00925
S_numerator[48,20]	0.00533	0.00170	0.00265	0.00925
S_numerator[49,20]	0.00533	0.00170	0.00265	0.00925
S_numerator[50,20]	0.00533	0.00170	0.00265	0.00925
S_numerator[51,20]	0.00533	0.00170	0.00265	0.00925
S_numerator[52,20]	0.00533	0.00170	0.00265	0.00925
S_numerator[53,20]	0.00533	0.00170	0.00265	0.00925
S_numerator[54,20]	0.00533	0.00170	0.00265	0.00925
S_numerator[55,20]	0.00533	0.00170	0.00265	0.00925
S_numerator[56,20]	0.00533	0.00170	0.00265	0.00925
S_numerator[57,20]	0.00019	0.00017	0.00002	0.00065
S_numerator[58,20]	0.00019	0.00017	0.00002	0.00065
S_numerator[59,20]	0.00019	0.00017	0.00002	0.00065
S_numerator[60,20]	0.00019	0.00017	0.00002	0.00065
S_numerator[61,20]	0.00019	0.00017	0.00002	0.00065
S_numerator[62,20]	0.00019	0.00017	0.00002	0.00065
S_numerator[63,20]	0.00019	0.00017	0.00002	0.00065
S_numerator[64,20]	0.00019	0.00017	0.00002	0.00065
S_numerator[65,20]	0.00019	0.00017	0.00002	0.00065
S_numerator[66,20]	0.00019	0.00017	0.00002	0.00065
S_numerator[67,20]	0.00019	0.00017	0.00002	0.00065
S_numerator[68,20]	0.00019	0.00017	0.00002	0.00065
S_numerator[69,20]	0.00019	0.00017	0.00002	0.00065
S_numerator[33,21]	0.00953	0.00681	0.00153	0.02729
S_numerator[34,21]	0.21351	0.11394	0.03640	0.47324
S_numerator[35,21]	0.14114	0.10523	0.00859	0.40749
S_numerator[36,21]	0.00407	0.00210	0.00109	0.00902
S_numerator[39,21]	0.01812	0.01066	0.00390	0.04392
S_numerator[40,21]	0.05947	0.03922	0.01022	0.16243
S_numerator[41,21]	0.31977	0.10381	0.12627	0.52907
S_numerator[44,21]	0.00351	0.00122	0.00163	0.00637
S_numerator[45,21]	0.00351	0.00122	0.00163	0.00637
S_numerator[46,21]	0.00351	0.00122	0.00163	0.00637
S_numerator[47,21]	0.00351	0.00122	0.00163	0.00637
S_numerator[48,21]	0.00351	0.00122	0.00163	0.00637
S_numerator[49,21]	0.00351	0.00122	0.00163	0.00637
S_numerator[50,21]	0.00351	0.00122	0.00163	0.00637
S_numerator[51,21]	0.00351	0.00122	0.00163	0.00637
S_numerator[52,21]	0.00351	0.00122	0.00163	0.00637
S_numerator[53,21]	0.00351	0.00122	0.00163	0.00637
S_numerator[54,21]	0.00351	0.00122	0.00163	0.00637
S_numerator[55,21]	0.00351	0.00122	0.00163	0.00637
S_numerator[56,21]	0.00351	0.00122	0.00163	0.00637
S_numerator[57,21]	0.00010	0.00010	0.00001	0.00036
S_numerator[58,21]	0.00010	0.00010	0.00001	0.00036
S_numerator[59,21]	0.00010	0.00010	0.00001	0.00036
S_numerator[60,21]	0.00010	0.00010	0.00001	0.00036
S_numerator[61,21]	0.00010	0.00010	0.00001	0.00036
S_numerator[62,21]	0.00010	0.00010	0.00001	0.00036
S_numerator[63,21]	0.00010	0.00010	0.00001	0.00036

© 2018 The Authors. Journal compilation © 2018 British Ecological Society, Ecology&Evolution

S_numerator[64,21]	0.00010	0.00010	0.00001	0.00036	S_numerator[67,22]	0.00005	0.00005	0.00000	0.00019
S_numerator[65,21]	0.00010	0.00010	0.00001	0.00036	S_numerator[68,22]	0.00005	0.00005	0.00000	0.00019
S_numerator[66,21]	0.00010	0.00010	0.00001	0.00036	S_numerator[69,22]	0.00005	0.00005	0.00000	0.00019
S_numerator[67,21]	0.00010	0.00010	0.00001	0.00036	S_numerator[33,23]	0.00464	0.00391	0.00053	0.01519
S_numerator[68,21]	0.00010	0.00010	0.00001	0.00036	S_numerator[34,23]	0.17022	0.10418	0.02089	0.41882
S_numerator[69,21]	0.00010	0.00010	0.00001	0.00036	S_numerator[35,23]	0.10755	0.09180	0.00389	0.35204
S_numerator[33,22]	0.00672	0.00521	0.00091	0.02065	S_numerator[39,23]	0.01032	0.00722	0.00163	0.02844
S_numerator[34,22]	0.19116	0.10929	0.02765	0.44620	S_numerator[40,23]	0.03874	0.02987	0.00481	0.12002
S_numerator[35,22]	0.12356	0.09859	0.00588	0.37986	S_numerator[41,23]	0.26798	0.10000	0.08986	0.47723
S_numerator[36,22]	0.00277	0.00157	0.00064	0.00650	S_numerator[44,23]	0.00140	0.00058	0.00056	0.00282
S_numerator[39,22]	0.01374	0.00881	0.00255	0.03551	S_numerator[45,23]	0.00140	0.00058	0.00056	0.00282
S_numerator[40,22]	0.04819	0.03436	0.00709	0.14012	S_numerator[46,23]	0.00140	0.00058	0.00056	0.00282
S_numerator[41,22]	0.29344	0.10219	0.10719	0.50330	S_numerator[47,23]	0.00140	0.00058	0.00056	0.00282
S_numerator[44,22]	0.00225	0.00085	0.00097	0.00429	S_numerator[48,23]	0.00140	0.00058	0.00056	0.00282
S_numerator[45,22]	0.00225	0.00085	0.00097	0.00429	S_numerator[49,23]	0.00140	0.00058	0.00056	0.00282
S_numerator[46,22]	0.00225	0.00085	0.00097	0.00429	S_numerator[50,23]	0.00140	0.00058	0.00056	0.00282
S_numerator[47,22]	0.00225	0.00085	0.00097	0.00429	S_numerator[51,23]	0.00140	0.00058	0.00056	0.00282
S_numerator[48,22]	0.00225	0.00085	0.00097	0.00429	S_numerator[52,23]	0.00140	0.00058	0.00056	0.00282
S_numerator[49,22]	0.00225	0.00085	0.00097	0.00429	S_numerator[53,23]	0.00140	0.00058	0.00056	0.00282
S_numerator[50,22]	0.00225	0.00085	0.00097	0.00429	S_numerator[54,23]	0.00140	0.00058	0.00056	0.00282
S_numerator[51,22]	0.00225	0.00085	0.00097	0.00429	S_numerator[55,23]	0.00140	0.00058	0.00056	0.00282
S_numerator[52,22]	0.00225	0.00085	0.00097	0.00429	S_numerator[56,23]	0.00140	0.00058	0.00056	0.00282
S_numerator[53,22]	0.00225	0.00085	0.00097	0.00429	S_numerator[57,23]	0.00002	0.00003	0.00000	0.00010
S_numerator[54,22]	0.00225	0.00085	0.00097	0.00429	S_numerator[58,23]	0.00002	0.00003	0.00000	0.00010
S_numerator[55,22]	0.00225	0.00085	0.00097	0.00429	S_numerator[59,23]	0.00002	0.00003	0.00000	0.00010
S_numerator[56,22]	0.00225	0.00085	0.00097	0.00429	S_numerator[60,23]	0.00002	0.00003	0.00000	0.00010
S_numerator[57,22]	0.00005	0.00005	0.00000	0.00019	S_numerator[61,23]	0.00002	0.00003	0.00000	0.00010
S_numerator[58,22]	0.00005	0.00005	0.00000	0.00019	S_numerator[62,23]	0.00002	0.00003	0.00000	0.00010
S_numerator[59,22]	0.00005	0.00005	0.00000	0.00019	S_numerator[63,23]	0.00002	0.00003	0.00000	0.00010
S_numerator[60,22]	0.00005	0.00005	0.00000	0.00019	S_numerator[64,23]	0.00002	0.00003	0.00000	0.00010
S_numerator[61,22]	0.00005	0.00005	0.00000	0.00019	S_numerator[65,23]	0.00002	0.00003	0.00000	0.00010
S_numerator[62,22]	0.00005	0.00005	0.00000	0.00019	S_numerator[66,23]	0.00002	0.00003	0.00000	0.00010
S_numerator[63,22]	0.00005	0.00005	0.00000	0.00019	S_numerator[67,23]	0.00002	0.00003	0.00000	0.00010
S_numerator[64,22]	0.00005	0.00005	0.00000	0.00019	S_numerator[68,23]	0.00002	0.00003	0.00000	0.00010
S_numerator[65,22]	0.00005	0.00005	0.00000	0.00019	S_numerator[69,23]	0.00002	0.00003	0.00000	0.00010
S_numerator[66,22]	0.00005	0.00005	0.00000	0.00019	S_numerator[33,24]	0.00314	0.00288	0.00029	0.01102

© 2018 The Authors. Journal compilation © 2018 British Ecological Society, Ecology&Evolution

S_numerator[34,24]	0.15076	0.09870	0.01550	0.39160
S_numerator[35,24]	0.09309	0.08496	0.00254	0.32498
S_numerator[39,24]	0.00768	0.00585	0.00101	0.02266
S_numerator[40,24]	0.03089	0.02576	0.00315	0.10203
S_numerator[41,24]	0.24355	0.09729	0.07431	0.45046
S_numerator[44,24]	0.00085	0.00038	0.00031	0.00179
S_numerator[45,24]	0.00085	0.00038	0.00031	0.00179
S_numerator[46,24]	0.00085	0.00038	0.00031	0.00179
S_numerator[47,24]	0.00085	0.00038	0.00031	0.00179
S_numerator[48,24]	0.00085	0.00038	0.00031	0.00179
S_numerator[49,24]	0.00085	0.00038	0.00031	0.00179
S_numerator[50,24]	0.00085	0.00038	0.00031	0.00179
S_numerator[51,24]	0.00085	0.00038	0.00031	0.00179
S_numerator[52,24]	0.00085	0.00038	0.00031	0.00179
S_numerator[53,24]	0.00085	0.00038	0.00031	0.00179
S_numerator[54,24]	0.00085	0.00038	0.00031	0.00179
S_numerator[55,24]	0.00085	0.00038	0.00031	0.00179
S_numerator[56,24]	0.00085	0.00038	0.00031	0.00179
S_numerator[57,24]	0.00001	0.00001	0.00000	0.00005
S_numerator[58,24]	0.00001	0.00001	0.00000	0.00005
S_numerator[59,24]	0.00001	0.00001	0.00000	0.00005
S_numerator[60,24]	0.00001	0.00001	0.00000	0.00005
S_numerator[61,24]	0.00001	0.00001	0.00000	0.00005
S_numerator[62,24]	0.00001	0.00001	0.00000	0.00005
S_numerator[63,24]	0.00001	0.00001	0.00000	0.00005
S_numerator[64,24]	0.00001	0.00001	0.00000	0.00005
S_numerator[65,24]	0.00001	0.00001	0.00000	0.00005
S_numerator[66,24]	0.00001	0.00001	0.00000	0.00005
S_numerator[67,24]	0.00001	0.00001	0.00000	0.00005
S_numerator[68,24]	0.00001	0.00001	0.00000	0.00005
S_numerator[69,24]	0.00001	0.00001	0.00000	0.00005
S_numerator[33,25]	0.00209	0.00209	0.00016	0.00786
S_numerator[34,25]	0.13281	0.09295	0.01130	0.36469
S_numerator[35,25]	0.08013	0.07818	0.00160	0.29878
S_numerator[40,25]	0.02444	0.02206	0.00205	0.08606
S_numerator[41,25]	0.22027	0.09410	0.06073	0.42434
S_numerator[44,25]	0.00050	0.00025	0.00017	0.00112
S_numerator[45,25]	0.00050	0.00025	0.00017	0.00112
S_numerator[46,25]	0.00050	0.00025	0.00017	0.00112
S_numerator[47,25]	0.00050	0.00025	0.00017	0.00112
S_numerator[48,25]	0.00050	0.00025	0.00017	0.00112
S_numerator[49,25]	0.00050	0.00025	0.00017	0.00112
S_numerator[50,25]	0.00050	0.00025	0.00017	0.00112
S_numerator[51,25]	0.00050	0.00025	0.00017	0.00112
S_numerator[52,25]	0.00050	0.00025	0.00017	0.00112
S_numerator[53,25]	0.00050	0.00025	0.00017	0.00112
S_numerator[54,25]	0.00050	0.00025	0.00017	0.00112
S_numerator[55,25]	0.00050	0.00025	0.00017	0.00112
S_numerator[56,25]	0.00050	0.00025	0.00017	0.00112
S_numerator[57,25]	0.00000	0.00001	0.00000	0.00002
S_numerator[58,25]	0.00000	0.00001	0.00000	0.00002
S_numerator[59,25]	0.00000	0.00001	0.00000	0.00002
S_numerator[60,25]	0.00000	0.00001	0.00000	0.00002
S_numerator[61,25]	0.00000	0.00001	0.00000	0.00002
S_numerator[62,25]	0.00000	0.00001	0.00000	0.00002
S_numerator[63,25]	0.00000	0.00001	0.00000	0.00002
S_numerator[64,25]	0.00000	0.00001	0.00000	0.00002
S_numerator[65,25]	0.00000	0.00001	0.00000	0.00002
S_numerator[66,25]	0.00000	0.00001	0.00000	0.00002
S_numerator[67,25]	0.00000	0.00001	0.00000	0.00002
S_numerator[68,25]	0.00000	0.00001	0.00000	0.00002
S_numerator[69,25]	0.00000	0.00001	0.00000	0.00002
S_numerator[33,26]	0.00136	0.00148	0.00008	0.00549
S_numerator[34,26]	0.11639	0.08702	0.00809	0.33827
S_numerator[35,26]	0.06860	0.07152	0.00099	0.27346
S_numerator[40,26]	0.01918	0.01875	0.00128	0.07166
S_numerator[41,26]	0.19823	0.09047	0.04926	0.39821
S_numerator[44,26]	0.00029	0.00015	0.00008	0.00067
S_numerator[45,26]	0.00029	0.00015	0.00008	0.00067
S_numerator[46,26]	0.00029	0.00015	0.00008	0.00067
S_numerator[47,26]	0.00029	0.00015	0.00008	0.00067
S_numerator[48,26]	0.00029	0.00015	0.00008	0.00067

© 2018 The Authors. Journal compilation © 2018 British Ecological Society, Ecology&Evolution

S_numerator[49,26]	0.00029	0.00015	0.00008	0.00067	S_numerator[54,27]	0.00016	0.00009	0.00004	0.00040
S_numerator[50,26]	0.00029	0.00015	0.00008	0.00067	S_numerator[55,27]	0.00016	0.00009	0.00004	0.00040
S_numerator[51,26]	0.00029	0.00015	0.00008	0.00067	S_numerator[56,27]	0.00016	0.00009	0.00004	0.00040
S_numerator[52,26]	0.00029	0.00015	0.00008	0.00067	S_numerator[57,27]	0.00000	0.00000	0.00000	0.00000
S_numerator[53,26]	0.00029	0.00015	0.00008	0.00067	S_numerator[58,27]	0.00000	0.00000	0.00000	0.00000
S_numerator[54,26]	0.00029	0.00015	0.00008	0.00067	S_numerator[59,27]	0.00000	0.00000	0.00000	0.00000
S_numerator[55,26]	0.00029	0.00015	0.00008	0.00067	S_numerator[60,27]	0.00000	0.00000	0.00000	0.00000
S_numerator[56,26]	0.00029	0.00015	0.00008	0.00067	S_numerator[61,27]	0.00000	0.00000	0.00000	0.00000
S_numerator[57,26]	0.00000	0.00000	0.00000	0.00001	S_numerator[62,27]	0.00000	0.00000	0.00000	0.00000
S_numerator[58,26]	0.00000	0.00000	0.00000	0.00001	S_numerator[63,27]	0.00000	0.00000	0.00000	0.00000
S_numerator[59,26]	0.00000	0.00000	0.00000	0.00001	S_numerator[64,27]	0.00000	0.00000	0.00000	0.00000
S_numerator[60,26]	0.00000	0.00000	0.00000	0.00001	S_numerator[65,27]	0.00000	0.00000	0.00000	0.00000
S_numerator[61,26]	0.00000	0.00000	0.00000	0.00001	S_numerator[66,27]	0.00000	0.00000	0.00000	0.00000
S_numerator[62,26]	0.00000	0.00000	0.00000	0.00001	S_numerator[67,27]	0.00000	0.00000	0.00000	0.00000
S_numerator[63,26]	0.00000	0.00000	0.00000	0.00001	S_numerator[68,27]	0.00000	0.00000	0.00000	0.00000
S_numerator[64,26]	0.00000	0.00000	0.00000	0.00001	S_numerator[69,27]	0.00000	0.00000	0.00000	0.00000
S_numerator[65,26]	0.00000	0.00000	0.00000	0.00001	S_numerator[33,28]	0.00054	0.00071	0.00002	0.00252
S_numerator[66,26]	0.00000	0.00000	0.00000	0.00001	S_numerator[34,28]	0.08800	0.07496	0.00389	0.28832
S_numerator[67,26]	0.00000	0.00000	0.00000	0.00001	S_numerator[35,28]	0.04951	0.05890	0.00035	0.22569
S_numerator[68,26]	0.00000	0.00000	0.00000	0.00001	S_numerator[40,28]	0.01157	0.01327	0.00048	0.04919
S_numerator[69,26]	0.00000	0.00000	0.00000	0.00001	S_numerator[41,28]	0.15818	0.08216	0.03163	0.34664
S_numerator[33,27]	0.00087	0.00103	0.00004	0.00376	S_numerator[44,28]	0.00009	0.00005	0.00002	0.00023
S_numerator[34,27]	0.10146	0.08100	0.00568	0.31317	S_numerator[45,28]	0.00009	0.00005	0.00002	0.00023
S_numerator[35,27]	0.05843	0.06508	0.00060	0.24880	S_numerator[46,28]	0.00009	0.00005	0.00002	0.00023
S_numerator[40,27]	0.01495	0.01583	0.00080	0.05958	S_numerator[47,28]	0.00009	0.00005	0.00002	0.00023
S_numerator[41,27]	0.17752	0.08647	0.03956	0.37246	S_numerator[48,28]	0.00009	0.00005	0.00002	0.00023
S_numerator[44,27]	0.00016	0.00009	0.00004	0.00040	S_numerator[49,28]	0.00009	0.00005	0.00002	0.00023
S_numerator[45,27]	0.00016	0.00009	0.00004	0.00040	S_numerator[50,28]	0.00009	0.00005	0.00002	0.00023
S_numerator[46,27]	0.00016	0.00009	0.00004	0.00040	S_numerator[51,28]	0.00009	0.00005	0.00002	0.00023
S_numerator[47,27]	0.00016	0.00009	0.00004	0.00040	S_numerator[52,28]	0.00009	0.00005	0.00002	0.00023
S_numerator[48,27]	0.00016	0.00009	0.00004	0.00040	S_numerator[53,28]	0.00009	0.00005	0.00002	0.00023
S_numerator[49,27]	0.00016	0.00009	0.00004	0.00040	S_numerator[54,28]	0.00009	0.00005	0.00002	0.00023
S_numerator[50,27]	0.00016	0.00009	0.00004	0.00040	S_numerator[55,28]	0.00009	0.00005	0.00002	0.00023
S_numerator[51,27]	0.00016	0.00009	0.00004	0.00040	S_numerator[56,28]	0.00009	0.00005	0.00002	0.00023
S_numerator[52,27]	0.00016	0.00009	0.00004	0.00040	S_numerator[57,28]	0.00000	0.00000	0.00000	0.00000
S_numerator[53,27]	0.00016	0.00009	0.00004	0.00040	S_numerator[58,28]	0.00000	0.00000	0.00000	0.00000

© 2018 The Authors. Journal compilation © 2018 British Ecological Society, Ecology&Evolution

S_numerator[59,28]	0.00000	0.00000	0.00000	0.00000	a	0.05406	0.00101	0.05212	0.05606
S_numerator[60,28]	0.00000	0.00000	0.00000	0.00000	b	1.60905	0.07409	1.47677	1.77621
S_numerator[61,28]	0.00000	0.00000	0.00000	0.00000	beta.data[1]	-0.53769	0.12075	-0.76520	-0.29202
S_numerator[62,28]	0.00000	0.00000	0.00000	0.00000	beta.data[2]	0.07214	0.14510	-0.19426	0.38356
S_numerator[63,28]	0.00000	0.00000	0.00000	0.00000	beta.data[3]	-0.33205	0.08674	-0.51706	-0.16433
S_numerator[64,28]	0.00000	0.00000	0.00000	0.00000	beta.data[4]	-0.44124	0.10074	-0.64860	-0.24631
S_numerator[65,28]	0.00000	0.00000	0.00000	0.00000	beta.data[5]	-0.40271	0.12139	-0.65948	-0.17366
S_numerator[66,28]	0.00000	0.00000	0.00000	0.00000	beta.data[6]	-0.40569	0.22763	-0.95635	-0.08000
S_numerator[67,28]	0.00000	0.00000	0.00000	0.00000	beta.data[7]	-0.72046	0.36574	-1.45830	-0.05255
S_numerator[68,28]	0.00000	0.00000	0.00000	0.00000	beta.data[8]	-0.39198	0.05805	-0.52891	-0.29596
S_numerator[69,28]	0.00000	0.00000	0.00000	0.00000	beta.data[9]	-0.23805	0.29712	-0.92729	0.22228
S_numerator[33,29]	0.00033	0.00048	0.00001	0.00166	beta.data[10]	-0.21432	0.11645	-0.47707	-0.02259
S_numerator[34,29]	0.07594	0.06900	0.00261	0.26376	beta.data[11]	0.67577	0.05760	0.54213	0.77093
S_numerator[35,29]	0.04175	0.05303	0.00020	0.20337	beta.data[12]	0.26461	0.13562	0.01858	0.55499
S_numerator[40,29]	0.00889	0.01107	0.00028	0.04013	beta.data[13]	-0.55017	0.43502	-1.50871	0.16850
S_numerator[33,30]	0.00020	0.00031	0.00000	0.00107	beta.data[14]	-0.75819	0.39496	-1.66669	-0.09519
S_numerator[34,30]	0.06522	0.06317	0.00172	0.24048	beta.data[15]	0.16811	0.18630	-0.19919	0.52615
S_numerator[35,30]	0.03505	0.04752	0.00011	0.18159	beta.data[16]	1.16808	0.05792	1.03489	1.26651
S_numerator[33,31]	0.00012	0.00020	0.00000	0.00068	beta.data[17]	0.96398	0.05813	0.83030	1.06169
S_numerator[34,31]	0.05574	0.05754	0.00112	0.21837	beta.data[18]	0.05778	0.13039	-0.19469	0.29882
S_numerator[35,31]	0.02928	0.04238	0.00006	0.16144	beta.data[19]	0.14523	0.13291	-0.11364	0.40767
S_numerator[33,32]	0.00007	0.00013	0.00000	0.00041	beta.data[20]	-0.57799	0.07361	-0.73866	-0.44689
S_numerator[34,32]	0.04741	0.05214	0.00071	0.19719	beta.data[21]	0.69217	0.07552	0.52987	0.83306
S_numerator[35,32]	0.02436	0.03762	0.00003	0.14235	beta.data[22]	1.46346	0.07130	1.30791	1.59366
S_numerator[33,33]	0.00004	0.00008	0.00000	0.00025	beta.data[23]	0.94469	0.19235	0.54001	1.30126
S_numerator[34,33]	0.04015	0.04703	0.00044	0.17753	beta.data[24]	1.36285	0.12207	1.11577	1.59469
S_numerator[35,33]	0.02018	0.03325	0.00002	0.12537	beta.data[25]	1.20344	0.11224	0.98133	1.41520
S_numerator[33,34]	0.00002	0.00005	0.00000	0.00015	beta.data[26]	0.22588	0.18133	-0.14471	0.57077
S_numerator[34,34]	0.03385	0.04222	0.00027	0.15851	beta.data[27]	0.70436	0.14187	0.41220	0.97462
S_numerator[35,34]	0.01665	0.02927	0.00001	0.10923	beta.data[28]	1.28072	0.09359	1.09539	1.45348
S_numerator[33,35]	0.00001	0.00003	0.00000	0.00009	beta.data[29]	0.63139	0.15866	0.32053	0.94227
S_numerator[34,35]	0.02842	0.03773	0.00016	0.14090	beta.data[30]	-0.47405	0.38129	-1.25418	0.26700
S_numerator[35,35]	0.01368	0.02566	0.00000	0.09538	beta.data[31]	-0.18950	0.42761	-1.06891	0.62370
S_numerator[33,36]	0.00001	0.00002	0.00000	0.00005	beta.data[32]	0.47178	0.23507	0.05521	0.93375
S_numerator[34,36]	0.02376	0.03358	0.00009	0.12544	beta.data[33]	0.08886	0.35732	-0.62933	0.72402
S_numerator[35,36]	0.01121	0.02241	0.00000	0.08215	beta.data[34]	-0.06300	0.35660	-0.86014	0.52476

© 2018 The Authors. Journal compilation © 2018 British Ecological Society, Ecology&Evolution

beta.data[35]	-0.81285	0.30917	-1.42084	-0.21338
beta.data[36]	0.69903	0.13615	0.42392	0.96561
beta.data[37]	1.23518	0.12219	0.99101	1.46853
beta.data[38]	0.80230	0.07753	0.64343	0.94576
beta.data[39]	1.32777	0.10956	1.10650	1.53727
beta.data.prec	3.04542	0.97411	1.51601	5.19115
beta.sp[1]	-0.13176	0.22063	-0.57370	0.29656
beta.sp[2]	-0.18991	0.30700	-0.84511	0.38151
beta.sp[3]	0.22517	0.20883	-0.19868	0.62707
beta.sp[4]	0.30497	0.30552	-0.26879	0.92225
beta.sp[5]	0.12543	0.35964	-0.58595	0.82464
beta.sp[6]	-0.29457	0.38954	-1.12741	0.39422
beta.sp[7]	0.30409	0.20637	-0.11887	0.69978
beta.sp[8]	0.86469	0.28950	0.31274	1.42822
beta.sp[9]	0.04681	0.41510	-0.83759	0.79669
beta.sp[10]	0.71287	0.33911	0.06936	1.40973
beta.sp[11]	0.33265	0.39889	-0.40022	1.16290
beta.sp.prec	8.73381	7.54919	1.41057	28.86869
beta.sub[1]	0.03937	0.18453	-0.32813	0.40507
beta.sub[2]	0.33956	0.24507	-0.13018	0.84637
beta.sub.prec	11.68272	9.71660	1.15601	37.64518
c	0.00513	0.00450	0.00018	0.01725
deviance	3708.517	10.82491	3688.875	3731.077
harvest[1]	0.10050	0.00497	0.08759	0.10571
harvest[2]	0.08730	0.01696	0.04286	0.10539
harvest[3]	0.10314	0.00266	0.09621	0.10579
harvest[4]	0.10256	0.00319	0.09395	0.10577
harvest[5]	0.10171	0.00398	0.09106	0.10574
harvest[6]	0.01428	0.01199	0.00041	0.04292
harvest[7]	0.06528	0.01866	0.01996	0.09049
harvest[8]	0.00102	0.00100	0.00003	0.00369
harvest[9]	0.04305	0.01766	0.00860	0.07621
harvest[10]	0.01428	0.00754	0.00154	0.02996
harvest[11]	0.00152	0.00150	0.00003	0.00553
harvest[12]	0.08897	0.01028	0.06319	0.09966
harvest[13]	0.04305	0.02617	0.00219	0.09425
harvest[14]	0.03052	0.02325	0.00101	0.08515
harvest[15]	0.00100	0.00057	0.00005	0.00194
harvest[16]	0.00010	0.00006	0.00001	0.00020
harvest[17]	0.00010	0.00006	0.00001	0.00020
harvest[18]	0.00100	0.00058	0.00005	0.00195
harvest[19]	0.00099	0.00058	0.00006	0.00195
harvest[20]	0.00010	0.00006	0.00001	0.00019
harvest[21]	0.00010	0.00006	0.00000	0.00020
harvest[22]	0.00010	0.00006	0.00001	0.00020
harvest[23]	0.02999	0.01952	0.00164	0.06679
harvest[24]	0.00010	0.00006	0.00001	0.00020
harvest[25]	0.00010	0.00006	0.00000	0.00019
harvest[26]	0.01159	0.00671	0.00058	0.02246
harvest[27]	0.01162	0.00670	0.00053	0.02240
harvest[28]	0.01161	0.00673	0.00055	0.02238
harvest[29]	0.00319	0.00185	0.00017	0.00619
harvest[30]	0.00098	0.00057	0.00005	0.00193
harvest[31]	0.00102	0.00058	0.00005	0.00194
harvest[32]	0.07275	0.02649	0.01018	0.10492
harvest[33]	0.06948	0.02770	0.00874	0.10475
harvest[34]	0.04166	0.02519	0.00212	0.08875
harvest[35]	0.00098	0.00057	0.00005	0.00195
harvest[36]	0.00101	0.00057	0.00005	0.00196
harvest[37]	0.00099	0.00058	0.00004	0.00196
harvest[38]	0.00101	0.00058	0.00005	0.00195
harvest[39]	0.00098	0.00058	0.00004	0.00195

© 2018 The Authors. Journal compilation © 2018 British Ecological Society, Ecology&Evolution
